# Anthropogenic landscapes and vector-borne disease dynamics: Unveiling the complex interplay between Human Footprint and disease transmission in Colombia

**DOI:** 10.1371/journal.pgph.0006801

**Published:** 2026-07-09

**Authors:** Juan D. Gutiérrez, Wendy L. Quintero-García, Yanyu Xiao, F. DeWolfe Miller, Diego F. Cuadros

**Affiliations:** 1 Facultad de Ciencias Médicas y de la Salud, Universidad de Santander, Instituto de Investigación Masira, Bucaramanga, Colombia; 2 Facultad de Ciencias Médicas y de la Salud, Departamento de Posgrado en Enfermedades Infecciosas, Universidad de Santander, Bucaramanga, Colombia; 3 Department of Mathematical Sciences, College of Arts and Sciences, University of Cincinnati, Cincinnati, Ohio, United States of America; 4 Department of Tropical Medicine, Medical Microbiology and Pharmacology, John A. Burns School of Medicine, University of Hawaii at Manoa, Honolulu, Hawaii, United States of America; 5 Digital Epidemiology Laboratory, Digital Futures, University of Cincinnati, Cincinnati, Ohio, United States of America; 6 Department of Biological Sciences, College of Arts and Sciences, University of Cincinnati, Cincinnati, Ohio, United States of America; PLOS: Public Library of Science, UNITED STATES OF AMERICA

## Abstract

Anthropogenic landscape transformations are fundamentally reshaping the epidemiology of vector-borne diseases (VBDs), yet their causal impacts remain poorly quantified across diverse ecological and socio-economic contexts. This study evaluates the causal effect of the Human Footprint (HFP) on the transmission dynamics of malaria, dengue, and visceral leishmaniasis in Colombia. We conducted an ecological analysis using municipal-level retrospective data from Colombia’s National Public Health Surveillance System (SIVIGILA, 2007–2019). Epidemiological records were integrated with environmental and socio-economic indicators, and a Double Machine Learning (DML) framework was applied to estimate the Average Treatment Effect (ATE) and Conditional Average Treatment Effect (CATE) of HFP on excess disease cases. Model robustness was assessed through refutation tests and non-parametric sensitivity analyses for unmeasured confounding. A one-standard-deviation increase in HFP significantly reduced the probability of excess malaria cases by 7.6 percentage points (ATE = -0.076, 95% CI: -0.094 – -0.058), an effect that was more pronounced in socio-economically deprived municipalities and modulated by temperature and precipitation gradients. Conversely, the ATE for dengue and visceral leishmaniasis was not different from zero. Robustness tests suggest the presence of residual bias, but the sensitivity test points to a low plausibility of an unobserved confounder. These findings underscore the necessity of integrating HFP monitoring into public health planning to design context-specific, multi-sectoral interventions that address the evolving landscape of VBD risk in rapidly transforming regions.

## Introduction

In an era of unprecedented urban expansion and environmental transformation, the global landscape of vector-borne diseases (VBDs) is undergoing dramatic reshaping. Understanding the impact of human activities on these diseases has become more crucial than ever. This study examines how the human footprint (HFP), quantified through changes such as deforestation, urbanization, and agricultural intensification, influences the transmission dynamics of malaria, dengue, and visceral leishmaniasis in Colombia. By employing causal inference and machine learning techniques, our research offers insights that challenge traditional approaches to disease prevention and public health planning, paving the way for more effective, context-specific interventions in rapidly changing environments.

In Colombia, sociopolitical evolution over recent decades, marked by urbanization driven by internal displacements, has further influenced the transmission dynamics of these diseases, emphasizing the need for a holistic understanding of their epidemiology [1]. Recognizing the profound influence of anthropogenic changes, from urban sprawl to deforestation, our study seeks to elucidate how these shifts directly impact the transmission and incidence of malaria, dengue, and visceral leishmaniasis in the country.

Human activities have driven unparalleled disturbances in natural ecosystems during the modern era. The concept of the “human footprint” encompasses a wide array of anthropogenic changes, including urbanization, deforestation, agricultural expansion, and infrastructure proliferation, which have left an indelible mark on over 95% of the Earth's terrestrial surface [2,3]. These transformations, while reshaping biodiversity, have also redefined human-environment interactions, making it imperative to understand the broader implications of such profound changes, especially in the context of the dynamic shift between natural and urban landscapes.

VBDs, transmitted by organisms like mosquitoes, ticks, and fleas, have historically posed significant public health challenges [4,5]. The emergence and re-emergence of these diseases, including malaria, dengue, and Zika, are intricately linked to environmental, socio-economic, and climatic determinants [6,7]. The HFP, with its multifaceted influence on land use, has become a key factor in the epidemiology of these diseases. Urbanization and deforestation alter vector habitats, potentially triggering outbreaks of VBDs [8]. Additionally, the ripple effects of global change, manifesting as altered weather patterns, further complicate the population dynamics of vectors, creating conditions that contribute to disease transmission and spatial spread [9].

Among the diseases that have shown heightened sensitivity to environmental shifts are malaria, dengue, and visceral leishmaniasis. Malaria is a febrile disease caused by different species of the *Plasmodium* spp., such as *P. vivax*, *P. falciparum*, and *P. malariae*, all of which are present in Colombia [10]. In this country, the parasite is transmitted by *Anopheles* mosquitoes [11]. Malaria remains endemic in the Americas, and the movement of infected individuals to malaria-free areas, creates a risk for disease re-emergence. Even with low transmission rates, such movement can cause local outbreaks and challenge public health systems [12]. This risk is further exacerbated by climate change, deforestation, and emerging resistance to conventional medicines [13].

Dengue is caused by four serotypes of the dengue virus, transmitted to humans by *Aedes aegypti* and *A*edes *albopictus* mosquitoes. Dengue infection is a major public health concern in tropical countries like Colombia, and its increasing frequency may be related, in part, to urbanization driven by social issues such as violence [14].

Leishmaniasis, caused by various species of the intracellular protozoan *Leishmania* spp. is transmitted primarily by nocturnal sandflies, although some exhibit crepuscular or diurnal activity in shaded environments, and is widely distributed throughout Latin America. Visceral leishmaniasis in Colombia is a zoonotic disease primarily caused by *Leishmania infantum* and transmitted by sandflies of the genus *Lutzomyia,* with *L. longipalpis* and *L. evansi* being the most important vectors [15]. These insects are particularly adapted to peri-urban environments, which provide optimal microclimates for their reproduction and development. Clinically, visceral leishmaniasis manifests as a systemic disease characterized by prolonged fever, hepatosplenomegaly, and pancytopenia [13].

The HFP encompasses both rural and urban human-induced modifications of ecosystems. In this context, it can serve as a common driver for VBDs. We aim to assess the impact of HFP on the incidence of malaria, dengue, and visceral leishmaniasis in Colombia. While, malaria, dengue, and visceral leishmaniasis differ markedly in their ecological and spatial distribution patterns across Colombia, our study explores whether HFP, as a composite measure of human pressure, can serve as a unifying environmental indicator to examine their transmission dynamics in a rapidly transforming landscape. By synergizing epidemiological data from Colombia's National Public Health Surveillance System (SIVIGILA) with environmental indices and socio-economic indicators, we employ an analytical framework that combines causal inference methods and machine learning techniques. This approach allows us to estimate the Average Treatment Effect (ATE) of HFP on disease incidence, as well as to explore the modifying roles of socio-economic and climatic factors on the effect of HFP on VBDs, and uncover disease-specific vulnerability patterns. By focusing on three diseases with distinct ecological niches within Colombia's diverse landscape, we offer insights into how VBDs respond to anthropogenic changes. The findings are expected to influence public health strategies by informing targeted, context-specific interventions that consider both ecological transformations and socio-economic variables, bridging the gap between theoretical knowledge and practical application in public health and environmental management.

## Methods

This research corresponds to an ecological study using municipalities as the unit of analysis and retrospective data from SIVIGILA (The national epidemiological surveillance system) between 2007 and 2019. By integrating epidemiological data with environmental and socio-economic metrics, we examine the association between the HFP and the incidence of VBDs across Colombian territory. Our approach combines historical data analysis with causal inference and machine learning techniques to explore the socioecological dynamics of malaria, dengue, and visceral leishmaniasis.

### Study design and data sources

We used data from SIVIGILA for the period 2007–2019 [16,17]. We included only laboratory-confirmed cases of malaria, dengue, and visceral leishmaniasis. The study integrated epidemiological data on malaria (All *Plasmodium* species were considered jointly), dengue, and visceral leishmaniasis with environmental and socio-economic metrics to examine the relationship between HFP and VBDs incidence across Colombian municipalities. All reported malaria cases, regardless of *Plasmodium* species (*P. falciparum, P. vivax*, or others), were aggregated and analyzed jointly to obtain total annual incidence rates and standardized incidence ratios at the municipal level. Relapse episodes associated with *P. vivax*, which have been recorded in SIVIGILA since 2014 under follow-up classifications, were not included in this analysis. Only new confirmed cases were used to estimate annual malaria incidence and standardized incidence ratios. For visceral leishmaniasis, SIVIGILA reports cases separately by clinical form, distinguishing between cutaneous and visceral leishmaniasis. For this study, we retrieved data exclusively from the visceral leishmaniasis reports, including only cases laboratory-confirmed according to SIVIGILA criteria. We used the infection municipality, as recorded in SIVIGILA, as the spatial localization criterion for disease cases. This approach allows for a more accurate reflection of transmission ecology, particularly in settings where residence or notification location may differ from the site of infection.

The HFP used in this study is based on the methodology developed by Mu et al. (2022) [18]. It integrates eight components that reflect human pressure on ecosystems: built environments, population density, electric infrastructure, croplands, pasturelands, roads, railways, and navigable waterways. Each component is normalized and weighted to generate a composite score ranging from 0 to 50, where 0 indicates pristine, undisturbed environments and 50 reflects areas of intense human modification. HFP data were available at a spatial resolution of 30 arcsec (~1 km^2^) and were averaged to the municipal level for analysis. Although some HFP components (e.g., croplands, pastures) may correlate with historical deforestation, the index reflects persistent anthropogenic pressures, while our forest coverage and deforestation data capture annual, dynamic land-use changes. These distinctions allow us to treat them as temporally and analytically distinct variables.

Environmental data, including the HFP, forest coverage, deforestation, and socio-economic indicators, were obtained from various national and global datasets described in Table 1. Note that socio-economic variables such as urban and rural misery, house deprivation, ethnic population, and population density were taken from the 2018 national census, the most recent available dataset. The variable urban dimension was included from a separate dataset provided by the National Department of Planning. According to the National Department of Statistics, a household is considered to be in a state of misery when its income is insufficient to meet basic needs (monetary poverty) or it experiences deprivation across multiple well-being dimensions (e.g., health, education, housing). In this study, the misery index is expressed as the percentage of households in a municipality classified as being in misery.

**Table 1:** Description of the variables included in the study.

Variable	Definition and Range	Unit of Measurement	Calculation/Source	Time Frame
**Human Footprint (HFP)**	A composite index measuring the cumulative impact of human activities, ranging from 0 (no impact) to 50 (maximum impact)	Index value	Derived from global datasets on land use, infrastructure, and access to roads [52]	Annually
**Excess Dengue Cases**	Binary variable measuring excess cases	Index value	Value = 1 when the SIR of dengue > 1, and 0 for any other value of the SIR	Annually
**Excess Malaria Cases**	Binary variable measuring excess cases	Index value	Value = 1 when the SIR of malaria > 1, and 0 for any other value of the SIR	Annually
**Excess Visceral Leishmaniasis Cases**	Binary variable measuring excess cases	Index value	Value = 1 when the SIR of visceral leishmaniasis > 1, and 0 for any other value of the SIR	Annually
**Forest Coverage**	Area covered by forest within a municipality	Percentage	Satellite imagery [53]	Annually
**Deforestation**	Area of forest cleared within a municipality	Percentage	Satellite imagery [54]	Annually
**Wildfires**	Area of wildfire incidents recorded in a municipality	Percentage	Satellite imagery [55]	Annually
**Illegal Mining**	Area affected by unauthorized mining operations in a municipality	Percentage	Satellite imagery [56]	Unique measure in 2020
**Coca Crops Coverage**	Area under coca cultivation as reported by anti-narcotics agencies	Percentage	National narcotics control agency data [57]	Annually
**Urban Misery**	A household in urban areas lacking two or more of the following characteristics: adequate housing, access to basic services, uncrowded living conditions, school attendance, and low economic dependency	Percentage of homes in misery	Last national census data [58]	Unique measure in 2018
**Rural Misery**	A household in rural areas lacking two or more of the following characteristics: adequate housing, access to basic services, uncrowded living conditions, school attendance, and low economic dependency	Percentage of homes in misery	Last national census data [58]	Unique measure in 2018
**Public Services**	Coverage of households in a municipality with access to public services	Percentage	Last national census data [58]	Unique measure in 2018
**House Deprivation**	A household in a municipality living in dwellings with structural deficiencies, overcrowding, or inadequate services or space	Percentage	Last national census data [58]	Unique measure in 2018
**Population Density**	Number of inhabitants in a municipality per unit of surface area	People/km^2^	Last national census data [58]	Unique measure in 2018
**Ethnic Population**	Number of inhabitants in a municipality who self-identify as part of an ethnic group	Percentage	Last national census data [58]	Unique measure in 2018
**Urban dimension**	A composite index measuring municipal population, rural population, population growth, population density, and belonging to agglomerations	Index value	Derived from national datasets [59]	Unique measure in 2015
**Standardized Incidence Ratio (SIR)**	Compare the observed incidence of the disease in a municipality to the expected incidence in the country. Used to estimate excess cases of each disease.	Ratio	Calculated using observed and expected incidence based on demographic data [60]	Annually
**Temperature**	Climate variable	°C	Climate reanalysis [61]	Annually
**Rainfall**	Climate variable	mm	Climate reanalysis [62]	Annually

### Data processing

We calculated the annual Standardized Incidence Ratio (SIR) for each disease using the R package epitools [19], with age standardization based on the World Health Organization (WHO) age groups [20]. Excess cases were defined as a binary variable (1 when SIR > 1, 0 otherwise). HFP was transformed into standard deviation (SD) units to facilitate the convergence of the estimation model.

### Causal inference and machine learning technique

We implemented the Double Machine Learning (DML) method to isolate the true causal effect of one factor of interest — in our case, the HFP — on an outcome, even when many other variables are simultaneously influencing both. This approach offers two important advantages over conventional statistical methods. First, it does not require the researcher to specify in advance the exact mathematical form of the relationships between confounders, the exposure, and the outcome — the algorithms learn these relationships directly from the data, capturing complex non-linear patterns that simpler methods would miss. Second, despite using these flexible algorithms for the preliminary prediction steps, the final estimate of the causal effect retains the statistical rigor needed to construct valid confidence intervals. All analyses were implemented using the EconML [21] and DoWhy [22] open-source packages in Python.

#### Causal model.

**Fig 1:**
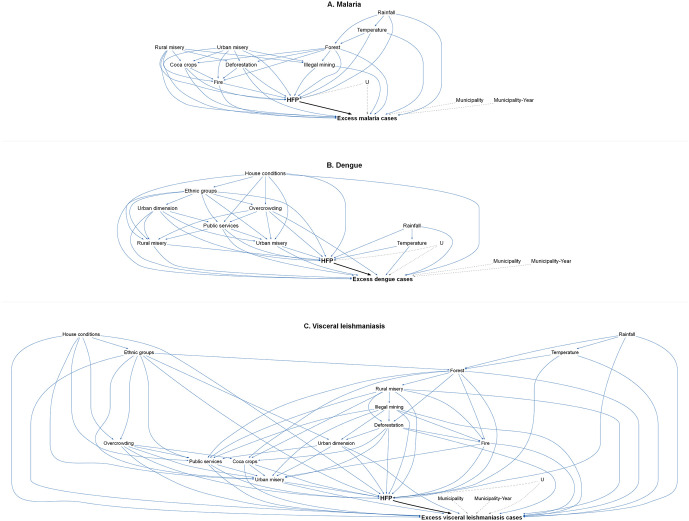
Directed acyclic graph (DAG) for the effect of human footprint (HFP) on excess cases of malaria (A), dengue (B), and visceral leishmaniasis (C). The causal association of interest is represented by the thick black arrow in each DAG and shows the effect of the exposure (HFP) on the outcome (excess cases). Dashed arrows departing from the U node represent unmeasured confounding bias due to unmeasured confounders.

For each disease, we constructed a Directed Acyclic Graph (DAG) to illustrate our existing knowledge regarding the interrelations among variables in the causal model and to clearly outline each causal assumption (Fig 1 and S1 File). In each DAG, the average annual HFP was designated as the exposure variable, while the annual excess cases of malaria, dengue, and visceral leishmaniasis served as the outcome variables.

We posited that the potential confounders differ for diseases primarily transmitted in rural (malaria) versus urban (dengue) environments. For visceral leishmaniasis, which is mainly transmitted in peri-urban environments, we assumed the confounding factors encompass influences from rural and urban contexts.

We introduced in the DAGs a potential confounder “U” to account for unmeasured variables absent from our dataset, addressing the remaining confounding bias in our estimates of HFP's effect on excess disease cases. Additionally, we controlled the spatio-temporal structure of the data (i.e., fixed effects) including the variables Municipality and Municipality-Year.

#### Model selection.

A central requirement of the DML approach is that the machine learning algorithms used to predict both the exposure (HFP) and the disease outcome from the confounders must perform as accurately as possible. If these predictive algorithms are too simple, residual confounding may bias the causal estimate; if they are overly complex, they may absorb a substantial portion of the variation in the exposure–outcome relationship, potentially leading to biased or distorted estimates. For this reason, before obtaining the causal estimates, we conducted a systematic procedure to identify the best-performing predictive configuration for each of the three diseases analyzed.

We used Extreme Gradient Boosting (XGBoost) as the machine learning algorithm for the predictive tasks. XGBoost constructs predictions by combining a number of simple decision trees in sequence, where each tree corrects the errors of the previous one. The behavior of this algorithm is governed by two key structural parameters: the number of trees used and the maximum depth of each tree (which controls how complex each individual correction step can be). Ten candidate configurations were defined by systematically varying these two parameters across plausible ranges: the number of trees was set to 20, 30, 50, 75, or 100, and the maximum tree depth was set to 4, 5, 7, or 8 levels, generating configurations of progressively increasing complexity. All other algorithm settings were kept identical across the ten candidates to ensure that any differences in performance were attributable exclusively to these structural choices (see details in S1 File). To evaluate and compare the ten configurations, we used five folds with cross-validation.

The criterion used to evaluate performance in each evaluation fold was the R-loss (residual loss), a metric specifically designed for causal inference settings. Unlike conventional predictive performance metrics that only measure how accurately an algorithm predicts the outcome, the R-loss simultaneously evaluates the quality of the prediction of both the exposure and the outcome, while also penalizing inaccurate estimation of the causal effect itself. This makes it the appropriate and theoretically justified criterion for selecting the best configuration within the DML framework. For each of the ten candidate configurations, the mean R-loss across the five evaluation folds was computed, along with its standard deviation to characterize estimation variability. The configuration yielding the lowest mean R-loss — indicating the best balance between predictive accuracy and reliable causal effect estimation — was selected as the optimal model for each disease. This entire selection procedure was conducted independently and identically for malaria, dengue, and visceral leishmaniasis.

#### Effect estimation.

Once the causal structure of each disease was formalized and the best model configuration was selected, the DML estimator was fitted to the dataset for each of the three diseases separately. The primary result of this estimation was the Average Treatment Effect (ATE), which represents the average change in the probability of a municipality experiencing excess disease cases associated with an increase of one SD of HFP, after removing the influence of all confounding factors included in the model. Alongside the ATE, a 95% confidence interval was computed using a resampling procedure in which the estimation was repeated across multiple random subsamples of the data, allowing uncertainty in the estimated effect to be quantified. A positive ATE indicates that greater human pressure on the environment is associated on average with a higher probability of excess disease cases, while a negative ATE indicates an inverse association.

Beyond estimating an overall average effect, we investigated whether the causal effect of the HFP on each disease was uniform across all municipalities or whether it varied depending on the socioeconomic and climatic context. To explore this, we estimated the Conditional Average Treatment Effect (CATE) — that is, how the causal effect of HFP on excess cases changes as a function of each of four contextual factors: the rural misery index, the urban misery index, rainfall, and temperature.

The estimation of context-specific effects was made possible by including a set of composite variables derived from all possible combinations and powers of the four contextual factors up to the third degree. This allowed the model to detect not only linear but also curved and interaction-driven patterns in how context shapes the causal effect of HFP. A regularization procedure (i.e., Lasso regularization with cross validation) was then applied to this expanded set of variables, automatically discarding those combinations that did not contribute meaningful information, and retaining only those that genuinely explained variation in the causal effect across contexts. This approach ensured that the final representation of context-specific effects was as simple as the data supported, avoiding the risk of over-interpreting random noise as meaningful patterns.

Our estimates regarding the impact of HFP on excess cases can be interpreted as causal, meaning they represent the potential outcome if the exposure were manipulated, and are free from bias under the following assumptions [23,24]: Unconfoundedness: Given all confounding factors, the potential outcomes are independent of the treatment assignment. Consistency: If an individual receives an exposure level, then the observed outcome is equal to the potential outcome under that same exposure level. Positivity: For all combinations of covariates within the study population, there exists a non-zero probability of receiving each exposure level. No Measurement Error: The exposure, outcome, and confounders are measured without significant error that could bias the results. Correct Model Specification: The statistical model is accurately specified, capturing all relevant relationships, including non-linearities and interactions.

#### Refutation analysis.

While the ultimate goal of any epidemiological research is to produce unbiased causal estimates, complete elimination of bias remains challenging in observational studies [23,25,26]. All such studies inherently face potential distortions from multiple sources, including confounding, misclassification, selection effects, and various forms of inference bias. In this study, we have employed several methods to address these biases, including causal machine learning techniques. Note that if our methods to address these biases were completely efficient, all types of bias would be tackled, and no signal of bias could be found in refutation tests. Nevertheless, our estimations may still be influenced by some of these biases. In this regard, we conducted a series of refutation tests to detect the presence of remaining bias in our estimates, even after implementing multiple strategies to mitigate different sources of bias.

To systematically evaluate the refutation of our causal conclusions, we employed four distinct refutation tests. These tests specifically assess whether causal estimates remain stable when subjected to conditions that should either preserve the effect (invariant transformations) or produce null effects (nullifying transformations).

In this context, remaining bias refers to systematic deviations in estimated effects that could compromise causal interpretation despite methodological safeguards. A well-specified causal model should demonstrate consistent estimates under non-threatening perturbations while producing null effects in scenarios designed to eliminate true causal relationships. Our specific refutation tests included:

1. Random common cause insertion: We introduced a synthetic binary variable that artificially influences both treatment and outcome without reflecting the true data-generating process. Significant changes in the original estimate would indicate vulnerability to unobserved confounding.
2. Replace a subset: We replaced 10% of the data with random values and recalculated the causal effect. A robust estimate should be insensitive to this manipulation.
3. Bootstrap resampling: The method uses repeated resampling techniques to characterize sampling variability and evaluate whether the estimated effect is influenced by unique characteristics of the observed sample.
4. Placebo treatment substitution: We replaced the actual treatment variable with a randomly generated binary variable. Any non-zero estimated effect would indicate that the model produces spurious associations.

The first three tests function as synthetic negative controls with invariant transformations, while the latter serves as negative controls with nullifying transformations. All tests were run 50 times. Following Rousselet et al [27], we employed Bootstrap methods to approximate the null distribution for each test condition. The resulting p-values indicate whether perturbed estimates significantly differ from the original (or from zero for placebo test). A p-value below 0.05 suggests the presence of residual bias in our original causal estimates, indicating potential limitations in our bias-mitigation strategies.

#### Assessment of the potential influence of unmeasured confounders.

Even after carefully accounting for all confounding factors included in the DAG, it is impossible in any observational study to guarantee that every relevant factor has been measured and incorporated. There may always exist factors that were not captured in the available data that could simultaneously influence both the level of human pressure on the environment and the occurrence of excess disease cases. If such unmeasured confounders existed and were sufficiently strong, they could distort the estimated causal effect of the HFP, making it appear larger, smaller, or even in the wrong direction.

To address this concern, we conducted a formal sensitivity analysis for each disease whose ATE was found to be different from zero, using the non-parametric partial R^2^ method, implemented within the DoWhy package. This analysis does not attempt to identify what specific unmeasured factors might exist; rather, it asks a more tractable and informative question: how strong would an unmeasured factor need to be in order to undermine the conclusions of the study? The answer is expressed in concrete, interpretable terms by comparing the required strength of any hypothetical unmeasured confounder against the strength of the most influential confounder that was already measured and included in the model.

#### Identifying the benchmark confounder.

As a first step, all measured confounders included in each disease model were ranked according to the degree to which they jointly predicted both the HFP and the disease outcome simultaneously. This ranking was obtained by computing the mutual information score using the package Scikit-learn. The confounder with the highest combined score was selected as the benchmark — that is, the reference standard against which the strength of any potential unmeasured confounder would be compared.

The actual strength of the benchmark confounder was then quantified using a five-fold cross-validation procedure, the portion of the variation in the HFP that could not be explained by all other confounders was computed, and similarly for the disease outcome. The degree to which the benchmark confounder was associated with these unexplained portions served as the reference point for the sensitivity analysis.

The non-parametric partial R^2^ test asks how strong an unmeasured confounder would need to be in order to make the estimated causal effect statistically indistinguishable from zero — that is, to render the finding no longer statistically meaningful at the conventional 95% confidence level. For each disease with ATE different from zero, the multiplier required to overturn the finding was reported alongside the actual strength of the benchmark confounder, allowing to judge whether an unmeasured confounder of such magnitude is ecological or socioeconomically plausible given what is known about each disease in the Colombian context.

### Data transformation and software

As mentioned above, HFP was transformed to standard deviation (SD) units, as were all covariates. Detailed methodologies, including the justification of the DAGs structure, implementation of DML, and interpretation of results, are provided in S1 File. We used Python packages EconML version 0.16 and DoWhy version 0.14 for causal inference and refutation tests.

## Results

**Fig 2:**
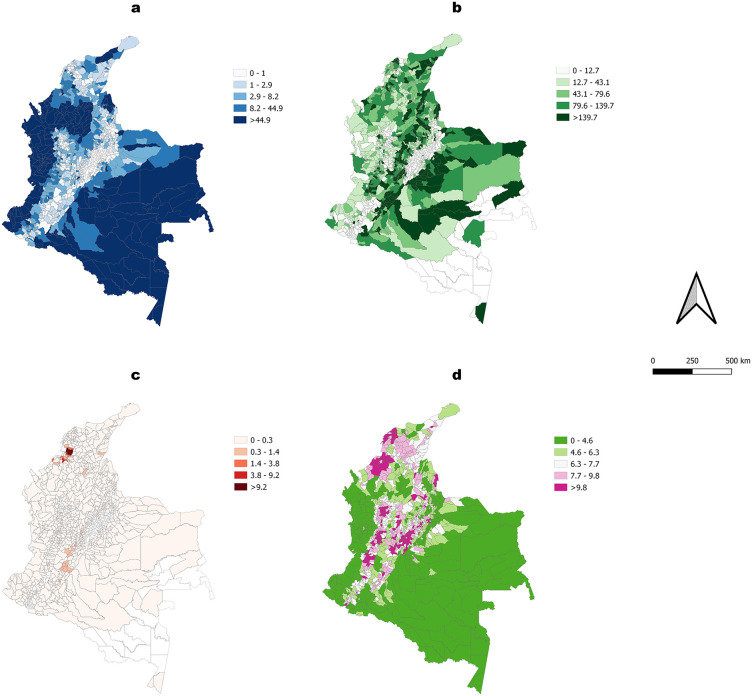
Municipal average standardized incidence ratio (SIR) for malaria (A), dengue (C), and visceral leishmaniasis (C) between 2007 and 2019, along with the average Human Footprint (HFP) for the same period (D). Base layer: Colombia municipal boundaries from geoBoundaries, Colombia ADM2, sourced from Departamento Administrativo Nacional de Estadistica (DANE) and distributed under the Creative Commons Attribution 4.0 International license (CC BY 4.0): https://www.geoboundaries.org/api/current/gbOpen/COL/ADM2/.

We observed distinct patterns in disease incidence across different regions of Colombia. The eastern and western parts of the country exhibited the highest average incidence of malaria. In contrast, the central region concentrated a high number of dengue cases. Meanwhile, the central and northern regions showed the highest incidence of visceral leishmaniasis. This geographical variation emphasizes the diverse distribution of these diseases across Colombia (Fig 2A-2C).

The HFP, indicative of anthropogenic changes, displayed varying spatial intensities across Colombia. The municipality with the lowest average HFP for the study period was Mirití (HFP = 0.22) in the Amazon region, while the municipality with the highest average HFP was Itagüí (HFP = 19.94) in Antioquia. Overall, the regions with the highest HFP values were concentrated in the center and north of the country (Fig 2D).

To identify the optimal specification of the DML model for effect estimation, ten distinct combinations of the number of trees and maximum depths were tested. Among these candidates, Model 1 achieved the lowest R-loss for malaria and dengue. Meanwhile, for visceral leishmaniasis, Model 2 was the best (S2 File). Therefore, we chose these models to estimate both the ATE and CATE.

**Fig 3:**
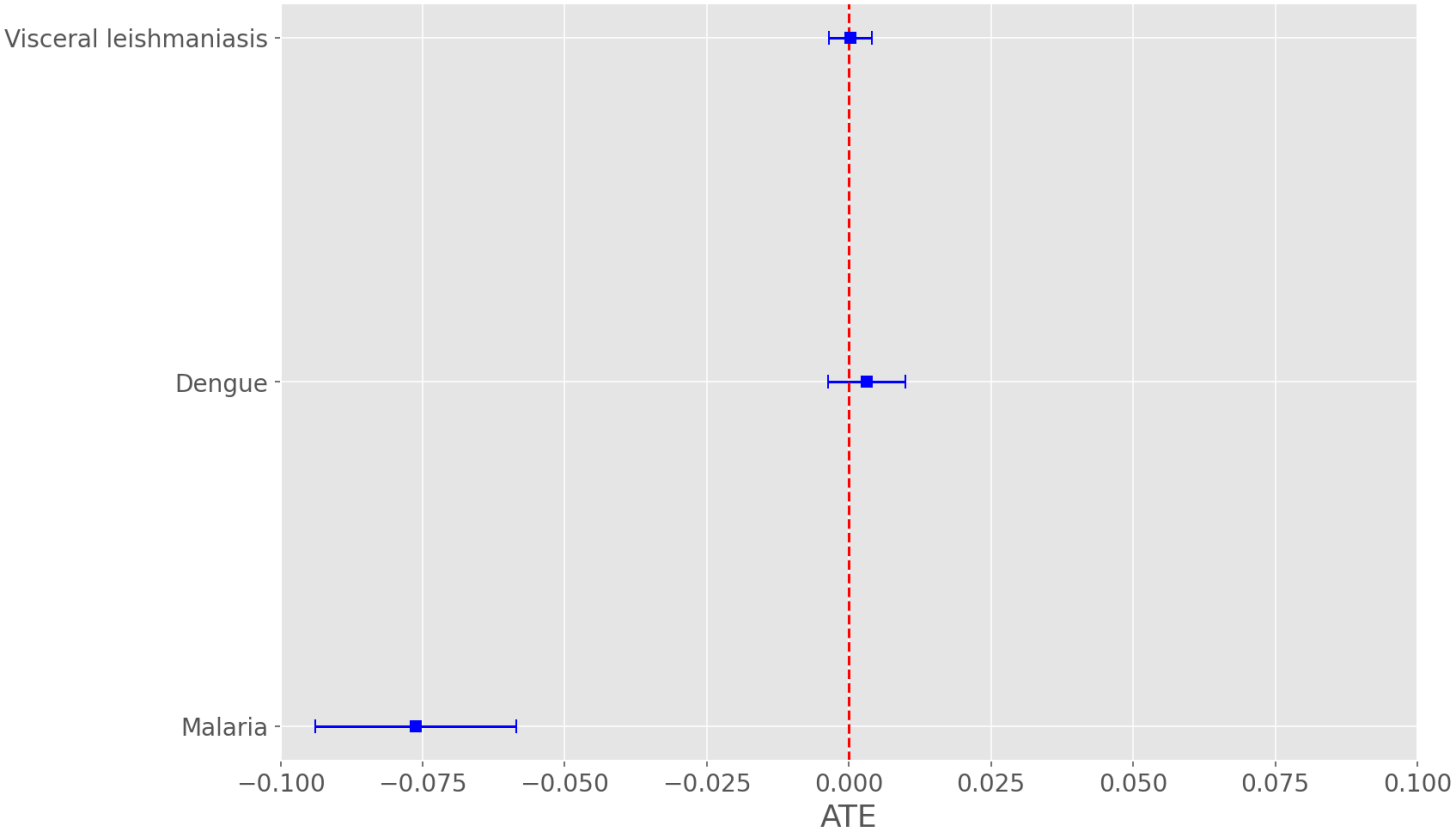
Average treatment effect (ATE) of the effect of human footprint (HFP) on excess cases of malaria, dengue, and visceral leishmaniasis. The effect corresponds to an increase of one standard deviation unit of HFP: malaria, 3.22 HFP points; dengue, 3.23 HFP points; visceral leishmaniasis, 3.24 HFP points. The x-axis depicts the ATE. Blue squares represent point estimates of the ATE, horizontal blue lines represent the 95% confidence interval (95% CI), and the red line represents the null effect (ATE = 0).

Our results revealed different patterns in the relationship between HFP and VBDs excess cases across Colombia. Malaria showed a negative effect; an increase of one SD of HFP (3.23 units) reduced the probability of excess malaria cases by 7.6 percentage points (ATE = -0.076, 95% CI: -0.094 – -0.058). In contrast, the ATE for dengue and visceral leishmaniasis was not different from zero, according to the 95% CI (Fig 3). Extrapolating the ATE of malaria to Colombia's 1,122 municipalities yields an estimated 85 municipalities (1,122 × 0.076 ≈ 85) within the disease's altitudinal range that could experience a reduction in excess cases per one SD increase in HFP.

**Fig 4:**
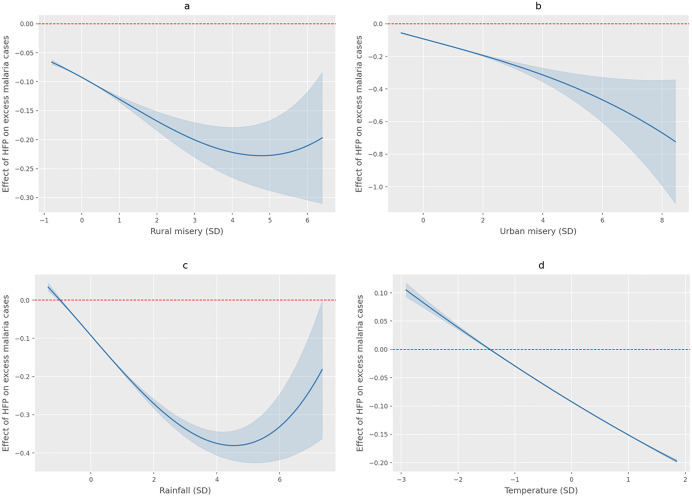
Conditional average treatment effect (CATE) of the effect of human footprint (HFP) on excess malaria cases, conditioned on: A) rural misery, B) urban misery, C) rainfall, and D) temperature. The blue line represents the average effect, the blue shaded area represents the 95% CI, and the dotted red line represents the null effect (CATE = 0). The CATE for each effect modifier is estimated at the average context of the other effect modifiers and within a spatiotemporal average framework.

**Fig 5:**
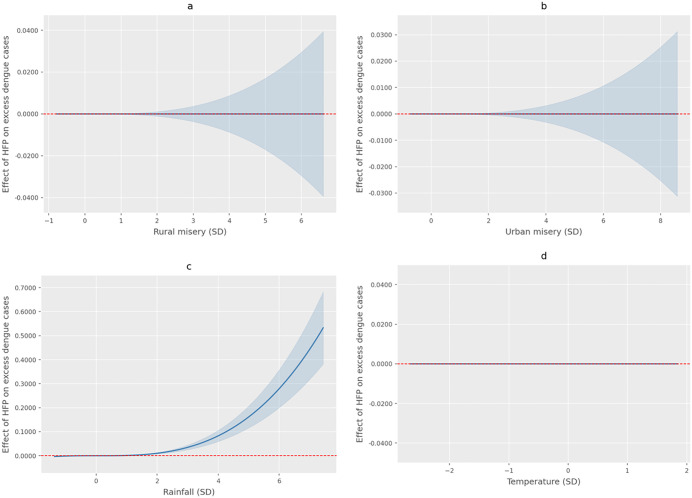
Conditional average treatment effect (CATE) of the effect of human footprint (HFP) on excess dengue cases, conditioned on: A) rural misery, B) urban misery, C) rainfall, and D) temperature. The blue line represents the average effect, the blue shaded area represents the 95% CI, and the dotted red line represents the null effect (CATE = 0). The CATE for each effect modifier is estimated at the average context of the other effect modifiers and within a spatiotemporal average framework.

**Fig 6:**
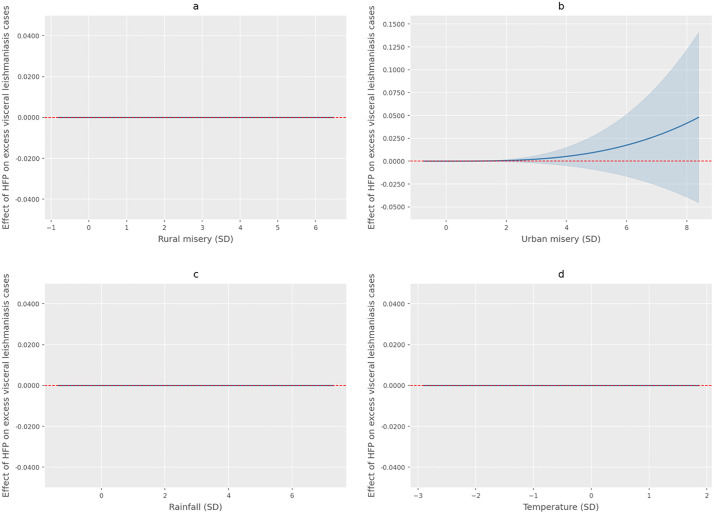
Conditional average treatment effect (CATE) of the effect of human footprint (HFP) on excess visceral leishmaniasis cases, conditioned on: A) rural misery, B) urban misery, C) rainfall, and D) temperature. The blue line represents the average effect, the blue shaded area represents the 95% CI, and the dotted red line represents the null effect (CATE = 0). The CATE for each effect modifier is estimated at the average context of the other effect modifiers and within a spatiotemporal average framework.

Note that in all CATE figures (Figs 4-6), regardless of the effect modifier that appears on the X-axis, what is being plotted is always the same: The strength of the causal effect of HFP on the excess of cases — free from the influence of all confounders — as a function of the context described by that effect modifier, keeping all other aspects of the context at their average value.

Regarding the CATE estimates, we observed that the effect of HFP on excess malaria cases, as a function of rural and urban misery, generally corresponds to a reduction in the probability of excess cases (Fig 4A and 4B). In municipalities with rainfall below 5 SD units (43.32 mm), HFP reduces the probability of excess cases; however, for rainfall values above this threshold, the probability of excess cases increases (Fig 4C). In the case of the effect of HFP conditioned on temperature, there is a trend toward a reduction in the probability of excess malaria cases as temperatures rise (Fig 4D).

The CATE for dengue was generally not significantly different from zero, according to the 95% confidence intervals (Fig 5A, 5B and 5D), except in the case of the CATE of the effect of HFP on excess dengue cases conditioned on rainfall, where higher rainfall values are associated with an increased probability of excess cases (Fig 5C). The CATE for visceral leishmaniasis was not significantly different from zero for all the effect modifiers evaluated (Fig 6A-6D).

Note that in some panels of Figs 5 and 6 (corresponding to the CATE of dengue and visceral leishmaniasis), the CATE is identically zero. This occurs because the model constructs a large set of variables derived from the six effect modifiers—rural and urban misery, rainfall, temperature, municipality, and municipality-year—including all pairwise combinations, as well as squared and cubic terms, yielding approximately 83 composite variables. The regularization procedure (i.e., LassoCV) then fits a model using these variables to estimate the CATE function, subject to a penalization mechanism. Specifically, Lasso shrinks the coefficients of variables that do not contribute sufficiently to explaining variation in the causal effect, potentially setting them exactly to zero. The penalty parameter is selected via cross-validation, choosing the value that optimizes out-of-sample predictive performance.

When the selected penalty is sufficiently large—typically in settings where the data provide little evidence of effect heterogeneity associated with a given modifier—all coefficients may be shrunk to exactly zero, i.e., the algorithm determines that there is not enough signal to differentiate the effect in different subgroups. Note that the ATE for dengue and visceral leishmaniasis is not significantly different from zero, consequently the causal signal is so weak that the regularization mechanism prefers total parsimony (null model) to avoid false discoveries.

Table 2 displays the findings from the four refutation tests conducted across the three diseases examined. In Table 2, the estimated effect reflects the original ATE derived from the main analysis, representing the average change in the probability of excess cases resulting from one SD increase in HFP, and the new effect indicates the re-estimated ATE following the application of each refutation test.

**Table 2:** Refutation tests implemented to evaluate the effect of HFP on the excess of cases of malaria, dengue, and visceral leishmaniasis. Note that an unbiased estimation means that the difference after adding a random common cause, replacing a random subset, and include a bootstrap resampling should be the minimum. Similarly, adding a placebo treatment should produce an estimation close to 0. Statistical significance is shown in the parentheses (p-value), a p-value < 0.05 suggests the presence of remaining bias in the estimate; p-values < 0.05 are in bold.

	Add a random common cause (p-value)	Replace a random subset (p-value)	Bootstrap resampling (p-value)	Add a placebo treatment (p-value)
**Malaria**	Estimated effect = -0.076	Estimated effect = -0.076	Estimated effect = -0.076	Estimated effect = -0.076
	New effect = -0.076 (0.18)	New effect = -0.073 (**<0.01**)	New effect = -0.084 (**<0.01**)	New effect = 0.000 (0.34)
**Dengue**	Estimated effect = 0.003	Estimated effect = 0.003	Estimated effect = 0.003	Estimated effect = 0.003
	New effect = 0.003 (0.24)	New effect = 0.049 (**<0.01**)	New effect = 0.044 (**<0.01**)	New effect = -0.000 (0.49)
**Visceral leishmaniasis**	Estimated effect = 0.0002	Estimated effect = 0.0002	Estimated effect = 0.0002	Estimated effect = 0.0002
	New effect = 0.0002 (0.24)	New effect = 0.0000 (**<0.01**)	New effect = 0.0093 (**<0.01**)	New effect = 0.0000 (0.31)

The p-values provided in parentheses assess whether the refutation test led to a statistically significant alteration in the estimated effect. If a refutation test yields p-values below 0.05 (indicating a significant difference from the original estimate), this points to the presence of residual bias.

The refutation tests of our estimates of the effect of HFP on excess cases of malaria, dengue, and visceral leishmaniasis showed that after replacing a random subset of 10% and bootstrap resampling, the new estimate was statistically different (p-value < 0.05) concerning the original estimation of the ATE, evidencing the presence of remaining bias in our estimates of the effect of HFP on the three diseases (Table 2).

The non-parametric partial R^2^ framework quantifies how strong an unmeasured confounder would need to be to invalidate the statistical significance of the ATE. As a benchmark confounder, we used the rural misery index, identified through mutual information analysis (MI_Score = 0.37) as the single most influential measured confounder simultaneously affecting both HFP (treatment) and excess malaria cases (outcome).

The non-parametric partial R^2^ of rural misery with respect to HFP, after partialling out all remaining covariates, was 0.115, meaning that rural misery explained approximately 11.5% of the residual variability in HFP not already accounted for by other measured variables. Similarly, the partial R^2^ of rural misery with respect to the excess malaria outcome was 0.227, indicating that rural misery accounted for 22.7% of the residual variability in excess malaria risk after conditioning on the remaining covariates.

The robustness value for statistical significance at the α = 0.05 level was RV_α = 0.990. This value represents the minimum joint partial R^2^ that a hypothetical unmeasured confounder would need to simultaneously explain with respect to both HFP and excess malaria cases in order to render the estimated effect statistically non-significant. Expressed as a multiplier relative to the benchmark confounder (rural misery), an unmeasured confounder would need to be 8.58 times stronger than rural misery in its association with HFP (the more demanding condition) and at least 4.36 times stronger in its association with excess malaria cases. Since rural misery is itself one of the most contextually plausible and empirically relevant confounder of both land-use pressure and excess malaria cases in the Colombian context, the existence of an unmeasured confounder with 8.58-fold greater confounding strength is highly implausible from a subject-matter perspective.

Note that the sensitivity analysis to assess the potential influence of unmeasured confounders was conducted exclusively for malaria. For dengue and visceral leishmaniasis, the overall causal effect (i.e., ATE) of the HFP on the excess disease cases was not distinguishable from zero — meaning that, after accounting for all confounding factors included in the model, there was no evidence that human pressure on the environment increased or decreased the probability of excess cases for these two diseases (Fig 3). Under these circumstances, conducting a sensitivity analysis to determine how strong an unmeasured factor would need to be to overturn the findings is not meaningful: there is no estimated effect to overturn. Asking how much external interference would be needed to bring an effect to zero when it is already zero provides no useful information about the robustness of the results. For this reason, the sensitivity analysis was not applicable to dengue and visceral leishmaniasis and was therefore not performed for these diseases.

As expected, when we binarized HFP using the median as the threshold (i.e., malaria: HFP > 6.76 units, dengue: HFP > 6.85 units, visceral leishmaniasis: HFP > 6.80 units), the effect was larger for malaria (ATE = -0.168, 95% CI = -0.190 to -0.147) and dengue (ATE = 0.074, 95% CI = 0.060 to 0.087), but for visceral leishmaniasis, the effect remained not significantly different from zero (ATE = 0.000, 95% CI = -0.001 to 0.001) (S3 File). However, note that the HFP binarized induces the loss of granular information and defeats the purpose of using HFP as a target variable for public health policies of VBDs.

## Discussion

To facilitate interpretation of our findings, we briefly restate the key causal estimands used in this study. The ATE quantifies the overall causal effect of a one SD increase in HFP on the probability of observing excess disease cases across all municipalities, averaged over the entire study population. In our analysis, the ATE provides a single summary measure of how anthropogenic landscape transformation affects VBDs incidence at the national scale, while accounting for potential confounders through the DML framework. In contrast, the CATE captures how this causal effect varies across different levels of specific contextual factors—in our case, rural misery, urban misery, rainfall, and temperature. By estimating the CATE, we were able to identify subpopulations and environmental contexts where the impact of HFP on disease transmission is amplified or attenuated, revealing critical effect modification patterns that would remain hidden in the average effect (i.e., ATE) alone. This distinction between ATE and CATE is essential for translating our findings into context-specific public health interventions, as it allows policymakers to identify municipalities where anthropogenic pressures pose the greatest disease risk, given their particular socioeconomic and climatic characteristics.

Our ATE results revealed varying relationships between HFP and excess cases of VBDs in Colombia. Malaria showed the strongest association: a one-SD increase in HFP reduced the probability of excess malaria cases by 7.6 percentage points, and seems concurring with the mainly sylvatic and rural transmission cycle of malaria in Colombia [28]. Conversely, for dengue and visceral leishmaniasis, the ATE did not differ from zero.

The observed pattern in panels A and B of Fig 4, in which the effect of HFP on excess malaria cases becomes progressively weaker as rural and urban misery increases, can be explained through the role of infrastructure in disease control. It is important to emphasize that this pattern does not imply that higher misery reduces malaria; the direct relationship between misery and disease burden was already absorbed as confounding during the causal estimation procedure. What panels A and B of Fig 4 reveal is that the same marginal increase in HFP produces a larger reduction in the probability of excess malaria cases when it occurs in a municipality characterized by higher levels of deprivation. This phenomenon is consistent with the fact that the disease disproportionately burdens the poorest populations, who are simultaneously the least likely to access malaria prevention and treatment services [12].

In municipalities with high rural or urban misery in Colombia, particularly those located in frontier agricultural zones, areas of armed conflict, and regions with limited state presence, the HFP baseline is typically very low, meaning that any measurable increment in the HFP probably reflects the arrival of the first formal elements of infrastructure: roads that enable supply chains for antimalarial medicines, electricity that sustains cold chain storage, and population density thresholds that make formal health service delivery economically viable. Compared with rural residents, urban and peri-urban communities have greater access to health workers, and wider access to healthcare facilities and services. In contexts where this infrastructure was previously absent, the marginal reduction in excess malaria cases of each additional SD unit of HFP is inherently greater than in municipalities where healthcare infrastructure already exists.

As rainfall increases to moderate levels (−1.3 to approximately 4 SD units, corresponding to 0.5 to ~37 mm per month), the availability of natural surface water bodies expands, malaria vector populations grow, and the baseline malaria burden, against which public health strategies associated with increases in HFP can operate, rises accordingly. In this precipitation range, the HFP is more likely to reflect formal urbanization, road connectivity, and access to health services. Within this regime, the combination of a substantial malaria burden, providing a large potential for case reduction, and a HFP composition dominated by health-care elements produces the most pronounced reduction in the probability of excess cases, explaining the progressively more negative CATE curve across this segment.

Several methodological and ecological considerations deserve explicit acknowledgement when interpreting the CATE pattern observed in panel C of Fig 4, particularly the apparent reversal toward a positive effect of HFP on excess malaria cases at rainfall values above approximately 5 SD units. First, from a statistical standpoint, the estimated CATE in that high-rainfall region should be interpreted with considerable caution. Inspection of the joint distribution of rainfall and HFP (S4 File) reveals that municipalities with extreme precipitation levels are represented by very few observations and exhibit a markedly reduced overlap in HFP values — concentrating in two polarized clusters rather than covering the full range of HFP values observed at moderate rainfall. This implies a partial violation of the positivity assumption, which requires a non-negligible probability of observing all treatment values across all covariate strata [29], means that the DML estimator is producing CATE estimates in a data-sparse region where the third-degree polynomial approximation of the effect function probably is extrapolating beyond its empirical support. The dramatic widening of the 95% confidence interval at the upper tail of the rainfall distribution in panel C of Fig 4 visually confirms this instability. Similarly, this extrapolation is compatible with the residual bias confirmed by the refutation tests which is expected to be most pronounced at the extremes of the effect modifier distribution, where both empirical support and estimation precision are lowest [22,30].

Second, the apparent sign reversal is probably further explained by a qualitative shift in the internal composition of the HFP index across the rainfall gradient. In Colombian municipalities with the highest precipitation levels, predominantly remote areas of the Pacific coast, the Chocó department, and the Amazonian piedmont [31], the HFP increments are driven primarily by illegal artisanal mining, frontier agriculture, and informal settlements lacking sanitary services, rather than by the formal urbanization, road connectivity, and health infrastructure that dominate the HFP signal in moderate-rainfall municipalities. Because the HFP is a composite index, the same numerical increment carries a fundamentally different causal meaning depending on which of its eight constituent components is driving it in a given climatic context; in extreme-rainfall zones, those driving components are precisely the ones known to generate artificial *Anopheles* breeding sites resistant to larval flushing [32,33], potentially counteracting the presence of health services that HFP otherwise represents. Note that this fact points to a possible violation of the assumption of consistency through the existence of multiple versions of the treatment [23,24].

Together, these two considerations suggest that the positive CATE segment observed at very high rainfall values should not be interpreted as a robust causal finding indicating that greater human pressure increases malaria risk in hyperhumid municipalities; rather, it reflects a region of genuine statistical and ecological uncertainty that future studies should address before any policy inference is drawn for those specific geographic and climatic contexts.

Probably an ecologically grounded interpretation of the pattern observed in panel D of Fig 4 rests not on a direct thermal modulation of HFP effectiveness, but on a fundamental shift in the eco-epidemiological regime of malaria across Colombia's temperature-altitude gradient. The strictly linear pattern observed points to a progressive compositional shift in what HFP epidemiologically represents across the altitudinal gradient.

Below thermal optimum, autochthonous malaria transmission becomes constrained or physiologically impossible: the Andean highland municipalities that characterize the negative temperature SD range are among the most formally urbanized in Colombia, exhibiting the highest HFP values nationally, driven by dense built environments, electric infrastructure, road networks, and high population density. In these thermally marginal settings, we hypothesize that malaria cases are probably predominantly of imported origin, introduced by infected travelers and internally displaced individuals arriving from lowland endemic regions [34]. In this epidemiological context, the road connectivity and population density components of HFP facilitate the human mobility and connectivity that underlie case importation, exposing large non-immune populations to *Plasmodium* and generating excess standardized incidence relative to expected baselines, thereby producing the positive CATE observed at the cold end of the gradient. Siraj et al. [35], confirmed that thermally marginal highland municipalities remain at persistent importation risk because temperature determines the receptivity threshold that governs whether an introduced parasite can or cannot initiate local transmission cycles of malaria in Colombia.

At warm positive temperature SD values, by contrast, corresponding to the lowland endemic municipalities of Caribbean region, increments in HFP capture formal urbanization processes: improved housing construction, proximity to health facilities enabling prompt diagnosis and antimalarial treatment, access to sanitation infrastructure, and overall improvements in living conditions that reduce human-vector contact. Feged-Rivadeneira et al. provided direct empirical support for this mechanism in Colombia, demonstrating with national surveillance data that *P. vivax* incidence declined significantly in major transmission hotspots that experienced moderate urbanization rates [36].

At the highest temperatures, mosquito mortality increases more rapidly, leading to a compressed infective lifespan and reduced vectorial capacity [37]. Similarly, early *Plasmodium* infection stages are known to be sensitive to high temperatures exceeding 30 °C, reducing the proportion of mosquitoes that successfully develop infectious sporozoites [38]. Consequently, the CATE of HFP converges toward a more negative effect at the upper thermal extreme, not only because of human infrastructure but also because the climate itself is already constraining transmission.

The pattern observed in panel C of Fig 5 is ecologically coherent and reflects a fundamental biological distinction between the vector of dengue and those of malaria and visceral leishmaniasis. Aedes aegypti breeds almost exclusively in artificial containers of standing water found in and around human dwellings [39]. This ecological specialization in man-made habitats has two critical consequences for the interpretation of this finding. Experimental studies demonstrated that immature stages of Ae. aegypti resist the flushing effect of rain substantially better than other vectors, with fourth-instar larvae being entirely unaffected by flushing under longer rain intervals, a resistance attributable to the physical protection afforded by enclosed or semi-enclosed domestic containers whose geometry prevents the turbulent displacement that characterizes open natural water bodies [39].

Additionally, rainfall acts as the key enabling factor that converts the structural characteristics of the urban environment captured by HFP into active vector breeding sites. The risk of dengue transmission increases with rapid, unplanned, and unregulated urban development, poor water storage practices, and unsatisfactory sanitary conditions [40]. However, at very low rainfall, this structural abundance of potential breeding sites seems epidemiologically inert, containers remain empty or dry and do not support larval development. As rainfall increases beyond approximately 2 SD units above the mean, containers could progressively fill and sustain standing water for periods sufficient to complete the aquatic development cycle of *Ae. Aegypti*, favoring the increase of breeding sites and dengue case. The effect of each additional unit of HFP on excess dengue cases therefore becomes progressively stronger as rainfall increases, because more containers are simultaneously filled and available for vector breeding. This pattern is further compounded by the disruption of piped water services that characterizes many Colombian municipalities, which leads to water storage practices [41].

An important consideration is the distinction between visceral and cutaneous leishmaniasis in Colombia’s surveillance system. Since SIVIGILA reports these two clinical forms separately, the present analysis was restricted to confirmed visceral leishmaniasis cases, which minimizes misclassification bias. Nonetheless, the different epidemiological dynamics of visceral and cutaneous leishmaniasis may influence their relationship with hydroclimatic and forest parameters. For instance, visceral leishmaniasis tends to occur in low-altitude, and peri-urban zoonotic transmission cycles, often linked to canine reservoirs, whereas cutaneous leishmaniasis is more frequently associated with sylvatic cycles involving other mammalian hosts [42,43]. Recognizing these differences is important for the interpretation of our findings, as environmental variables may differentially affect each clinical form. Thus, our results should be understood within the specific eco-epidemiological context of visceral leishmaniasis.

In addition, compared with the study conducted by Skinner et al. [2], in which they explored a broader spectrum of diseases in relation to HFP, our research narrows its focus, concentrating on malaria, dengue, and visceral leishmaniasis, three diseases of regional significance with divergent ecologies. Unlike Skinner, we apply a causal machine learning framework and incorporate socioeconomic and environmental factors, allowing us to identify not only associations but also potential causal relationships between human-induced environmental changes and disease incidence. This precision-driven methodology allowed us to elucidate distinct patterns and intricacies associated with these diseases, enhancing our understanding of their interactions with anthropogenic environmental modifications.

### Limitations of the study

This study has several limitations worth noting. SIVIGILA serves as the backbone for our epidemiological data collection, covering a wide array of health data reported by medical professionals from across the country [17]. Despite updates and advancements, challenges remain. Underreporting persists in rural and hard-to-reach areas, where limited connectivity, shortages of trained personnel, and logistical barriers can compromise reporting [44]. These limitations are important to acknowledge, as they may introduce bias or underestimation in disease burden estimates. Additionally, the indirect method to estimate the SIR, while robust, it cannot mitigate all factors associated to the information bias in an ecological study based on secondary information. Particularly in rural or heterogeneous environments where health surveillance and data reporting might be less consistent.

Moreover, the spatial resolution of the HFP data may not capture all possible micro-environmental changes that significantly affect the habitats of vectors. Furthermore, the complexity of the transmission cycles of malaria and visceral leishmaniasis, involving various hosts and environmental interactions, might dilute or obscure the direct impact of HFP. Addressing these limitations requires more granular data and possibly the incorporation of additional ecological and climatic variables to refine our understanding of how human-induced environmental changes affect the incidence of these complex VBDs.

A methodological limitation of this study relates to the statistical assumptions underlying the DML implementation. The DML framework, assumes that observations are independent and identically distributed (i.i.d.) [45], a condition that underpins the cross-fitting procedure and the asymptotic validity of the resulting confidence intervals. In the present study, however, the unit of observation is the Colombian municipality measured repeatedly across thirteen consecutive years (2007–2019), generating a panel dataset in which observations belonging to the same municipality share unobserved cluster-level that induce within-municipality correlation over time. This dependency structure constitutes a violation of the i.i.d. assumption embedded in the standard EconML implementation, because the cross-fitting folds are drawn at random without regard to the municipality grouping, allowing correlated residuals from the same cluster to appear simultaneously in training and evaluation sets [46].

Ignoring this clustering structure leads to a systematic underestimation of the true sampling variance of the causal effect estimator and, consequently, to confidence intervals that are artificially narrow and Type I error rates that exceed the nominal level [47]. Although the inclusion of municipality and municipality-year fixed effects as effect modifiers partially absorbs mean-level heterogeneity across clusters, these fixed effects do not correct the variance-covariance matrix of the causal effect estimator itself, which remains affected by within-municipality residual correlation [48].

Another methodological limitation of the present study concerns the operational definition of the outcome variable. Excess disease cases were defined as a binary variable based on whether the age-adjusted SIR exceeded 1. However, because the three vector-borne diseases examined here exhibit markedly different incidence levels and highly heterogeneous spatial distributions across Colombian municipalities, a non-trivial number of units may have small expected case counts. In such settings, a very small observed cases can produce a numerically large SIR that superficially appears to indicate excess disease burden while remaining fully consistent with random Poisson variation around the null hypothesis of SIR = 1. This phenomenon implies that the crude SIR is an unstable estimator in low-incidence areas, and that binarizing it without accounting for its statistical uncertainty may introduce systematic misclassification of the outcome which in turn could bias the causal effect estimates derived from the DML framework.

Future studies should address this limitation by adopting the Bayesian hierarchical spatial model proposed by Besag, York, and Mollié [49], fitted through Integrated Nested Laplace Approximations (INLA). This framework models observed case counts as Poisson-distributed with a log-relative risk decomposed into a spatially structured component, governed by an intrinsic conditional autoregressive process that borrows statistical strength from neighboring municipalities, and an unstructured random effect capturing residual heterogeneity. The resulting posterior distribution of the relative risk for each municipality enables a statistically principled and uncertainty-aware redefinition of excess cases: a municipality would be classified as exhibiting excess disease burden only when the lower bound of the 95% posterior credible interval for its smoothed SIR exceeds 1, thereby ensuring that the binary outcome is assigned exclusively to units where the evidence for elevated risk is sufficiently strong to rule out random variation [50].

### Broader implications for public health policy and disease management

Understanding the influence of HFP on the dynamics and trajectories of VBDs can pave the way for more targeted and effective interventions, especially in regions undergoing rapid urban transformation. Our research underscores the imperative for a holistic approach to disease control, an approach that transcends biological considerations and examines into the socio-ecological contexts that underpin disease transmissions [51].

An integrated public health strategy that combines disease surveillance, vector control, environmental, and socio-economic development is crucial. Such an approach necessitates cross-sectoral collaboration between health, urban planning, and social services to ensure a coordinated and effective response. Our findings suggest that monitoring human footprint dynamics could improve the geographic targeting of interventions and resource distribution, refining public health strategies to better align with localized ecological and socio-economic conditions [2].

Furthermore, the variation in disease response to HFP highlights the necessity for differential policy approaches. Accordingly, this study provides empirical evidence that highlights the specific ways in which human footprint dynamics differentially affect malaria, dengue, and visceral leishmaniasis. By quantifying these effects at the municipal level, we aim to refine the spatial and contextual focus of existing interventions. Integrated strategies should focus on preserving natural habitats to maintain the ecological balance, thereby controlling the vector populations. This could involve managing deforestation, illegal mining, and agricultural expansion in ways that minimize the disruption of vector habitats. Concurrently, community engagement and education about the risks and prevention of these diseases are crucial in areas where traditional knowledge and practices can significantly influence disease outcomes.

## Conclusions

The results of our study cannot be considered fully causal and free of bias, given the findings of the refutation tests; however, they provide insights into the complex dynamics between human-induced environmental changes, as captured by the HFP, and the transmission patterns of VBDs, particularly malaria, dengue, and visceral leishmaniasis. In our main ATE analysis, HFP showed an effect of reduction of the probability of excess cases of malaria, indicating that increases in HFP, reflective of urbanization, infrastructure development, and other anthropogenic changes, correlate with an average reduction in malaria cases. This trend underscores the sensitivity of malaria transmission to urban environmental changes, likely due to the mainly rural-adapted nature of its primary vector. In contrast, no effects were observed in the incidence of dengue and visceral leishmaniasis, in relation to an increase of one SD of the HFP.

Looking forward, our findings pave the way for several key directions in research and policy. Future studies should aim to deepen the understanding of how micro-environmental changes within urban and rural landscapes affect disease vectors and pathogens. This involves incorporating more granular data on ecological and climatic variables to refine causal models of disease incidence. For policy, there is a pressing need to develop integrated disease management strategies that address the socio-economic and environmental determinants of VBDs. These strategies should be tailored to the specific needs of urban and rural communities, ensuring that interventions are both effective and sustainable.

## Supporting information

S1 File Supplementary methods. Data sources, DAG justification tables, model selection details, machine learning implementation, robustness analysis, and unmeasured-confounding sensitivity analysis.
(DOCX)

S2 File Model selection results. R-loss results for the hyperparameter optimization strategy used to select the XGBoost nuisance-model configurations for malaria, dengue, and visceral leishmaniasis.
(TXT)

S3 File Average treatment effects using binarized HFP. ATE estimates for the effect of HFP on excess cases after binarizing HFP at the disease-specific median threshold.
(DOCX)

S4 File Overlap assessment for rainfall and HFP in malaria. Joint distribution of rainfall and HFP showing limited empirical support at extreme precipitation values and the resulting positivity concern.
(DOCX)
