# Supplementary material for: Anthropogenic landscapes and vector-borne disease dynamics: Unveiling the complex interplay between Human Footprint and disease transmission in Colombia
Source: PLOS Glob Public Health. 2026 Jul 9;6(7):e0006801. doi: 10.1371/journal.pgph.0006801 (PMC13349180; doi:10.1371/journal.pgph.0006801)
Supplement: S1 File — Data sources, DAG justification tables, model selection details, machine learning implementation, robustness analysis, and unmeasured-confounding sensitivity analysis. (DOCX) [file pgph.0006801.s001.docx]

**Supplement 1**

Juan D. Gutiérrez, Wendy L. Quintero-García, Yanyu Xiao, F. DeWolfe Miller, Diego F. Cuadros

1. **SUPPLEMETARY METHODS**
   1. ***Data sources***

The primary focus of our study was Colombia, a country characterized by its diverse ecosystems and varying land-use pressures. The unique socio-economic contexts of this country have significantly influenced its urbanization patterns, making it an ideal setting to study the impact of human footprint on vector-borne diseases, particularly for diseases adapted to urban (dengue), rural (malaria), and peri-urban (visceral leishmaniasis) environments.

- 1. ***Epidemiological data***

We obtained annual reported cases of malaria, dengue, and visceral leishmaniasis from SIVIGILA. SIVIGILA is designed to monitor, analyze, and generate timely responses to public health risks and events across Colombia, providing a critical foundation for public health decision-making and policy formulation. This system gathers comprehensive data from various health entities, including hospitals, clinics, and laboratories nationwide, ensuring broad coverage of health events and conditions of public concern. The dataset from SIVIGILA includes detailed information on notifiable diseases, encompassing a range of infectious diseases such as malaria, dengue, and visceral leishmaniasis, the focus of our study. For each reported case, the dataset provides demographic information, clinical data, and outcomes, which are essential for understanding disease patterns and their implications. Additionally, the system organizes data by epidemiological weeks, allowing for the analysis of disease trends and the timing of outbreaks with precision.

Health professionals across Colombia are required to report cases of notifiable diseases promptly, according to the severity of the disease and its potential impact on public health. This mandatory reporting ensures that the data is as comprehensive and current as possible, enabling real-time surveillance and rapid public health responses. Furthermore, SIVIGILA employs advanced tools for data analysis, including geographic information systems (GIS), which are crucial for mapping disease distribution and understanding the spatial dimensions of disease transmission.

The fields considered at SIVIGILA in our query were: Infection municipality, Date, Age, and Sex. The filters applied were: Cases confirmed by laboratory analysis, Cases dated between 2007 and 2019, Cases that occurred in rural areas (Dispersed rural areas, and rural population centers), and municipal capitals.

For malaria, data includes instances confirmed via laboratory tests identifying the *Plasmodium* parasite. Dengue data collection encompasses laboratory confirmations, detailing the severity of the disease and patient outcomes. Similarly, leishmaniasis data procurement involves recording clinical diagnoses substantiated by lab tests for *Leishmania* parasites. The data encompassed a total of 828,268 reported cases of malaria, 365,663 cases of dengue, and 294 cases of visceral leishmaniasis spanning from 2007 to 2019 at the municipality level. To avoid allochthonous cases in the analysis, we excluded cases reported in municipalities above 1,600 meters above sea level for malaria, 2,300 meters for dengue, and 1,700 meters for visceral leishmaniasis, as these are the altitudinal thresholds for the three diseases included in the study. We also excluded cases missing municipality or date information.

We estimated the annual standardized incidence ratio (SIR) for each disease using the R package epitools. The incidence was age-standardized by the indirect method, according to the age groups of WHO and the population estimations of the National Department of Statistics. We estimated the excess cases for each disease as a binary variable, with a value of 1 when the SIR > 1, and 0 for any other value of the SIR.

- 1. ***Environmental and socio-economic data***

To understand the broader context of disease transmission, we sourced environmental and socio-economic data from various datasets. A key component of this was the global record of the annual HFP index. The HFP provided a multidimensional perspective, capturing the cumulative impact of human activities, including local environments, energy and transportation infrastructure, agricultural lands, and human population density. We obtained raster layers of the global record of the annual terrestrial HFP dataset for the period from 2007 to 2019, with a spatial resolution of 30 arcsec. The HFP includes eight variables (built environments, population density, electric infrastructure, croplands, pasture lands, roads, railways, and navigable waterways) to estimate the human pressure on ecosystem function and integrity, on a continuous scale from 0 to 50, where 0 corresponds to a pristine ecosystem. For each municipality, the annual average HFP was estimated by spatial matching among the raster files and the polygon of the municipalities, using the R package raster.

We obtained annual raster layers of forest coverage (NASA product MCD12Q1), deforestation, wildfires (NASA product MCD64A1), and illegal mining for the period 2007–2019. We estimated the annual percentage of forest coverage, deforestation, wildfires, and illegal mining for each municipality through spatial matching as mentioned above. The percentages of rural and urban misery, coverage of public services, overcrowding, ethnic population, and house condition deprivation of each municipality were downloaded from the Terridata repository in the national census of 2018. The annual percentage of area with coca crops in each municipality was obtained from the repository of open data of the Colombian government. The urban dimension of each municipality was obtained from the National Department of Planning and corresponds to a quantitative estimation of social and economic activities in the Colombian municipalities.

- 1. ***Directed acyclic graph***

A central challenge in studying the relationship between human activity and infectious disease is that the factors that drive human pressure on the environment — such as poverty, deforestation, illegal mining, or urbanization — are often the same factors that independently increase the risk of disease. This creates a situation in which a simple comparison of municipalities with high versus low HFP will not yield a reliable answer about whether human pressure itself is truly causing more disease cases, because any observed association may reflect the influence of these shared underlying factors rather than a genuine causal relationship.

In a causal inference framework using the Backdoor Criterion to identify and block backdoor paths, the goal is to estimate the effect of an exposure variable (HFP) on an outcome variable (excess cases), while systematically accounting for potential confounders (e.g., forest coverage, deforestation, socio-economic variables) that might bias the observed relationship, and simultaneously avoiding inducing other forms of bias (e.g., collider bias). Unlike conventional regression analysis, which primarily focuses on identifying associations between variables, causal inference explicitly targets the underlying mechanisms that drive these relationships. This involves the use of techniques such as counterfactual reasoning to isolate the true causal effect of the exposure by adjusting for valid confounding factors [26]. In essence, while regression methods remain valuable for prediction, the causal inference approach is designed to uncover cause-and-effect relationships by carefully considering and mitigating sources of bias.

We developed a directed acyclic graph (DAG) for each disease to show our prior knowledge about the relationship between the variables in the causal model and to explicitly present each causal assumption. In each DAG, we included the average annual HFP as the exposure variable and the annual excess cases of malaria, dengue, and visceral leishmaniasis as the outcome variable, respectively.

We assumed that the set of potential confounders is different for diseases whose transmission cycle is mainly rural or urban, as is the case for malaria and dengue, respectively. Similarly, we assumed that for a disease with peri-urban cycle, as is the case of visceral leishmaniasis, the potential confounders are the sum of rural and urban confounders. Lastly, we included a potential confounder "U" in each DAG to represent the unmeasured variables that are not included in our dataset, corresponding to the remain confounding bias in our estimations of the effect of HFP on the excess cases of each disease.

The next tables provide the justification for the chosen structure and variables of each DAG.

Table 1. Justification of the malaria DAG structure.

| **Association** | **Causal justification** | **Reference** |
| --- | --- | --- |
| Rainfall → Temperature | Precipitation influences local Temperature through changes in humidity, cloud cover, and energy balance | [1] |
| Rainfall → Forest | Precipitation determines primary productivity and forest cover | [2] |
| Temperature → Forest | Temperature regulates plant growth, evapotranspiration, and biome distribution | [3] |
| Rainfall → HFP | Climatic conditions determine human habitability and land use patterns | [4] |
| Forest → HFP | Forest availability determines the level of human intervention and land use |  |
| Deforest → HFP | Deforestation increases infrastructure development, road construction, and human activity |  |
| Rural misery → HFP | Rural poverty conditions intensive land use and resource exploitation |  |
| Temperature → HFP | Temperature influences population density and human activities | [5] |
| Urban misery → HFP | Urban poverty influences human expansion and settlement patterns |  |
| Rainfall → excess | Rainfall increases mosquito breeding sites and malaria transmission | [6] |
| Temperature → excess | Temperature controls parasite and vector development | [7] |
| Forest → excess | Forest cover defines vector habitats and human exposure | [8] |
| Forest → Deforest | Deforestation occurs in previously forested areas | [9] |
| Rural misery → Deforest | Rural poverty drives natural resource use and agricultural expansion | [10] |
| Urban misery → Deforest | Urban demand for natural resources contributes to deforestation | [11] |
| Rural misery → Fire | Rural poverty promotes agricultural practices involving the use of fire |  |
| Deforest → excess | Deforestation modifies vector habitats and increases malaria risk | [12] |
| Forest → Coca | Illicit crops are primarily established in forested areas | [13] |
| Coca → Fire | Illicit agricultural expansion involves vegetation burning |  |
| Rural misery → Coca | Rural poverty is a structural determinant of illicit crop cultivation | [14] |
| Coca → HFP | Illicit crops involve infrastructure development, settlements, and human disturbance |  |
| Urban misery → Coca | Socioeconomic factors and urban demand influence production | [15] |
| Coca → excess | Expansion of illicit crops is associated with malaria in Amazon regions | [16] |
| Forest → Mining | Mining activities expand primarily in forested regions | [17] |
| Mining → HFP | Mining generates human settlements and landscape alteration |  |
| Rural misery → Mining | Rural poverty drives artisanal mining activities | [18] |
| Mining → excess | Mining creates mosquito breeding sites and increases malaria risk | [19] |
| Deforest → Fire | Deforestation increases the risk of fires | [20] |
| Urban misery → Fire | Population pressure increases fire occurrence | [21] |
| Fire → HFP | Fires alter land use patterns and human activity |  |
| Forest → Fire | Forests provide fuel loads for fires |  |
| Fire → excess | Fires modify vector habitats and human exposure | [22] |
| Rural misery → excess | Poverty increases vulnerability and exposure to malaria | [23] |
| Urban misery → excess | Urban poverty is associated with increased malaria risk |  |
| Municipality/Municipality-Year → excess | Fixed effects adjustment | [24] |

Table 2. Justification of the dengue DAG structure.

| **Association** | **Causal justification** | **Reference** |
| --- | --- | --- |
| Rainfall → Temperature | Precipitation influences local humidity and thermal balance | [1] |
| Temperature → HFP | Temperature influences habitability and population density | [5] |
| Rainfall → excess | Rainfall increases breeding sites for *Aedes aegypti* | [25] |
| Temperature → excess | Temperature controls viral and vector development | [26] |
| House conditions → Ethnic | Residential segregation is associated with socioeconomic and ethnic characteristics | [27] |
| Ethnic → HFP | Historically marginalized ethnic groups show different levels of territorial intervention |  |
| Ethnic → Public services | Minority groups have reduced access to basic Public services |  |
| Ethnic → excess | Structural inequality generates higher dengue risk | [28] |
| Rural misery → excess | Poverty increases exposure and reduces vector control capacity |  |
| Urban dimension→ excess | Urbanization facilitates dengue transmission | [29] |
| Urban dimension → Public services | Urban expansion determines infrastructure and service availability | [30] |
| House conditions → Public services | Housing characteristics determine access to basic Public services |  |
| House conditions → Overcrowding | Housing quality influences House conditionshold density |  |
| Overcrowding → Public services | High population density affects service availability |  |
| Ethnic → Overcrowding | Sociospatial segregation increases overcrowding |  |
| Urban dimension→ Rural misery / Urban misery | Unplanned urbanization generates inequality | [31] |
| Ethnic → Urban dimension | Ethnic distribution is associated with patterns of urbanization |  |
| Urban misery → HFP | Urban poverty increases population density and vulnerability |  |
| Overcrowding → excess | Overcrowding increases exposure to mosquito vectors | [32] |
| House conditions → excess | Poor housing conditions favor domestic mosquito breeding sites |  |
| Urban misery → excess | Urban poverty is a key determinant of dengue risk |  |
| Public services → excess | Lack of safe water and sanitation promotes mosquito breeding sites |  |
| Overcrowding → Rural misery / Urban misery | Overcrowding is an indicator of poverty | [33] |
| Public services → Rural misery / Urban misery | Lack of Public services is a direct indicator of poverty |  |
| Ethnic → Rural misery / Urban misery | Ethnic inequality is associated with structural poverty |  |
| House conditions → Rural misery / Urban misery | Housing conditions are structural indicators of poverty |  |
| Public services → HFP | Access to infrastructure reflects human territorial intervention | [4] |
| Rural misery → HFP | Poverty conditions human settlement patterns |  |
| Overcrowding → HFP | Population density is a component of the human footprint |  |
| Rainfall → HFP | Climate determines settlement patterns and land use |  |
| House conditions → HFP | Housing conditions reflect the level of human intervention in the territory |  |
| Urban dimension → HFP | Urbanization increases the human footprint |  |
| Municipality/Municipality-Year → excess | Fixed effects adjustment | [24] |

Table 3. Justification of the visceral leishmaniasis DAG structure.

| **Association** | **Causal justification** | **References** |
| --- | --- | --- |
| Temperature → Forest | Temperature regulates vegetation productivity and distribution | [3] |
| Rainfall → Temperature | Precipitation influences local thermal dynamics and microclimates that determine vector ecology | [1] |
| Rainfall → HFP | Climate variability conditions land use and human activity | [34] |
| Temperature → HFP | Thermal conditions affect human activities and territorial expansion |  |
| Forest → excess | Modification of forest habitats increases human–vector contact |  |
| Deforest → HFP | Deforestation is a direct manifestation of the human footprint |  |
| Fire → HFP | Forest fires are a consequence of human activities |  |
| Fire → excess | Fires modify vector hábitats |  |
| Mining → HFP | Mining is an intensive form of land modification |  |
| Mining → excess | Extractive activities increase exposure to vectors |  |
| Mining → Deforest | Mining is an important driver of deforestation |  |
| Rural misery → Public services | Poverty limits access to basic Public services |  |
| Urban dimension → Public services | Urbanization determines service availability |  |
| Rainfall → excess | Rainfall modifies vector abundance and survival | [35] |
| Temperature → excess | Temperature is a direct determinant of vector distribution |  |
| Urban dimension → excess | Urban expansion may increase transmission |  |
| Public services → excess | Lack of sanitation facilitates vector reproduction |  |
| Rainfall → Forest | Precipitation determines vegetation cover and forest density | [2] |
| Forest → HFP | Forest presence conditions human activities and territorial expansion | [36] |
| Deforest → excess | Deforestation alters ecosystems and increases transmission |  |
| Rural misery → HFP | Poverty drives informal occupation of land |  |
| Rural misery → excess | Leishmaniasis is strongly associated with poverty |  |
| Urban misery → HFP | Urban poverty influences informal urban expansion |  |
| Urban misery → excess | Adverse socioeconomic conditions increase infection risk |  |
| House conditions → excess | Poor housing conditions favor transmission |  |
| Overcrowding → excess | Overcrowding increases exposure to vectors |  |
| Coca → HFP | Illicit crops involve land-use transformation | [14] |
| Coca → excess | Agricultural expansion promotes human–vector contact | [36] |
| Deforest → Coca | Deforestation facilitates agricultural expansion | [14] |
| Municipality/Municipality-Year → excess | Fixed effects adjustment | [24] |

- 1. ***Best model selection***

To evaluate and compare the ten configurations, we used a procedure known as cross-validation. This procedure works as follows: the full dataset for each disease was randomly divided into five subsets of approximately equal size. For each candidate configuration, the model was trained on four of the five subsets and then evaluated on the one remaining subset that had not been used for training — a subset the model had never seen before. This process was repeated five times, each time using a different subset as the evaluation set, so that every observation in the dataset served as part of both the training and the evaluation process at some point. The key advantage of this approach is that model performance is always assessed on data that were entirely independent from those used to fit the model, providing a reliable and unbiased estimate of how well each configuration would generalize to new, unseen cases.

To estimate the causal effect of the HFP on excess disease cases, we implemented a Double Machine Learning (DML) framework, which allows for valid inference of the Conditional Average Treatment Effect (CATE) in the presence of high-dimensional confounding. Following the principles of causal identification, we constructed disease-specific DAGs for malaria, dengue, and visceral leishmaniasis using the DoWhy library. Within this structure, we prioritized the backward adjustment path, identifying valid confounders based on their simultaneous association with both the exposure (HFP) and the outcome (excess cases), thereby ensuring that the identified estimand was non-parametrically identifiable.

This method decomposes the problem into two nuisance models: a classifier tasked with predicting the outcome (excess cases), and a regressor tasked with predicting the treatment (HFP). To handle non-linearities and high-order interactions among covariates and heterogeneity features, we utilized a third-degree PolynomialFeaturizer within the final stage of the DML. The nuisance models were implemented using eXtreme Gradient Boosting (XGBoost), which provides the necessary flexibility to capture complex architectural patterns in the data.

Model selection was conducted through a hyperparameter optimization strategy. We evaluated ten candidate configurations for the XGBoost learners, varying the number of estimators (ranging from 20 to 100) and the maximum depth (ranging from 4 to 8). The performance of each configuration was assessed using a 5-fold cross-validation scheme. To ensure the robustness of the CATE estimates and prevent overfitting, we incorporated an internal 3-fold cross-fitting process. The final model selection was determined by minimizing the R-loss (residual loss), a specialized metric that evaluates the accuracy of the causal effect estimation by measuring the squared difference between the outcome residuals and the treatment residuals scaled by the treatment effect.

To guarantee scientific rigor and exact reproducibility, we enforced strict stochastic control across all computational experiments. Global seeds were fixed for all random number generators, and the XGBoost tree_method was set to exact to avoid the variance inherent in histogram-based approximations. Furthermore, computational parallelism was disabled by restricting the execution to a single thread and setting environment variables to limit low-level library threads. This configuration ensures that the reported R-loss values and the resulting optimal hyperparameters are deterministic across different execution environments.

- 1. ***Machine learning implementation***

Identified the best specific architectural parameters for each disease model, the heterogeneity of the treatment effect was modeled using a Lasso-regularized linear estimator combined with a third-degree polynomial expansion. This approach allowed the framework to capture non-linear interactions between the HFP and the heterogeneity features. By employing LassoCV with a targeted grid of alpha values—ranging from 0.0001 to 0.1—we ensured automated feature selection within the expanded polynomial space, maintaining model parsimony and preventing overfitting of the causal estimates.

We utilized a 5-fold external cross-validation to evaluate the generalization of the models across the dataset. Within each fold, a 3-fold internal cross-fitting procedure was executed to strictly decouple the estimation of the nuisance parameters from the treatment effect estimation. This procedure is critical for eliminating the bias that typically arises from using the same data for both model training and formal causal inference.

The Average Treatment Effect (ATE) and the Conditional Average Treatment Effect (CATE) were calculated by simulating the response of excess cases to a one-standard-deviation shift in the HFP. While the ATE provided a population-level mean effect, the CATE allowed for the identification of specific environmental and demographic strata where the effect of the HFP is intensified. The model integrated a comprehensive set of covariates—including rainfall, temperature, rural and urban misery indicators—serving simultaneously as confounders and heterogeneity features to isolate the causal signal.

Finally, we enforced a strict deterministic protocol to guarantee the reproducibility of the results. Global seeds were fixed for all stochastic processes, and the XGBoost tree_method was set to exact to eliminate the variance inherent in approximate histogram methods. Furthermore, to prevent non-deterministic outcomes from parallel execution, we restricted the models to a single thread (nthread=1) and manually limited the thread usage of underlying numerical libraries. These controls ensure that the identified optimal hyperparameters and the resulting causal estimates remain identical across different computational environments.

Note that in our study, the ATE represents the average effect of the HFP on the excess cases of each disease for the entire set of municipalities included in the analysis. The heterogeneous effect refers to the idea that the effect of a particular treatment/exposure can vary across different individuals or subgroups within a population; in our study, it means that the effect of HFP on excess cases of each disease is different for each municipality. Finally, the CATE represents the average effect of the HFP for a specific subgroup defined by a covariate, for example, the percentage of rural misery. In other words, the CATE allows us to estimate exposure effects for different subgroups within the set of municipalities.

- 1. ***Robustness analysis***

We assessed the estimates through four refutation tests to validate the causal association between HFP and the excess cases of each disease included in the study. In our analysis, we employed four key refutation tests to validate the robustness of our causal inferences regarding the impact of the human footprint on vector-borne disease incidence. These tests are crucial for ensuring that our findings are not artifacts of model specifications or peculiarities in the data. The tests implemented were:

- *Adding a random common cause*: This simulates the introduction of an unrelated factor that might influence both the treatment and outcome variables. A valid estimate should remain largely unaffected.
- *Replacing a random subset*: A small portion of the data (10%) was replaced with random values. A robust estimate should be relatively insensitive to this manipulation.
- *Bootstrap resampling:* It involves employing repeated sampling methods to assess the variability in sampling and determine if the estimated effect is affected by the specific traits of the observed sample.
- *Adding a placebo treatment*: This introduces a fictitious treatment group with no actual intervention. A valid estimate should show no difference between the treatment and placebo groups.

These methods aim to simulate various types of errors or perturbations to check if the estimated causal effect is unbiased and to assess the sensitivity and validity of the causal inferences. Note that for an unbiased estimation, after adding a random common cause, replacing a random subset, and a bootstrap resampling the values should be close to the estimated value. The inclusion of a placebo should give a new estimation close to 0. A value of significance (p-value) < 0.05 is considered evidence of the presence of latent bias in the estimate.

For the addition of a random common cause, the subset replacement, and the bootstrap resampling a p-value exceeding 0.05 implies that the re-estimated effect (new effect) is statistically comparable to the original ATE, indicating no evidence of remaining bias. In tests that involve the incorporation of a placebo treatment, the expected causal effect is zero under the null hypothesis. In such scenarios, a new effect close to zero and a p-value above 0.05 suggest that the estimation procedure does not yield spurious associations in the absence of a genuine causal relationship, thereby reinforcing its validity and suggesting the absence of remaining bias.

- 1. ***Effect of an unmeasured confounder***

To assess the vulnerability of the estimated causal effects to omitted confounders, a non-parametric sensitivity analysis was performed based on the partial coefficient of determination R^2^. This framework quantifies the requisite strength of an unobserved confounder—in terms of its association with both the treatment (HFP) and the outcome (excess cases)—to nullify the reported point estimates. By leveraging the residuals from the DML process, this approach extends traditional linear sensitivity metrics to a non-parametric setting, ensuring consistency with the flexible XGBoost architecture used in the primary analysis.

The methodology relies on the calculation of the partial R^2^ of the treatment and the outcome, which measure the proportion of residual variance explained by a hypothetical unobserved factor. To provide a grounded interpretation of these values, the analysis incorporated an observed confounder to serve as a comparative benchmark. This benchmark was selected for each disease model by identifying the observed confounder that demonstrated the highest simultaneous predictive power for both the exposure and the outcome. This selection ensures that the sensitivity threshold is calibrated against the most influential known confounder in the system, providing a conservative and empirically justified scale for the potential effect of hidden confounding bias.

Using this benchmarked scale, we calculated the Robustness Value (RV), a summary metric that defines the minimum strength of association an unobserved confounder must have with both the treatment and the outcome to move the causal estimate to zero. An RV significantly higher than the partial R^2^ of the most relevant observed confounders suggests that the identified causal signal is robust, as an unobserved confounder would need to possess implausibly high explanatory power to invalidate the findings. This procedure allows for a quantification of the unconfoundedness assumption inherent in causal inference.

Furthermore, the sensitivity analysis estimated the bounding effects under varying degrees of hypothetical confounding. By calculating how the point estimate and its associated 95% confidence intervals would shift if an unobserved variable were k-times as strong as the selected benchmark, we mapped the stability of the ATE. This stress-testing ensures that the conclusions regarding the impact of the HFP are not merely artifacts of the specific set of included covariates but remain stable even under violations of the ignorability assumption.

Finally, the results were synthesized into a formal sensitivity profile, reporting the limit at which the causal effect loses statistical significance. This assessment, provides a transparent measure of scientific certainty. By confirming that the RV exceeds the empirical benchmarks established by the most significant observed environmental and socioeconomic confounders, the analysis validates the reliability of the ATE and CATE estimates in the presence of complex, high-dimensional data structures.

**References**

1. Trenberth KE, Dai A, Rasmussen RM, Parsons DB. The Changing Character of Precipitation. Bull Am Meteorol Soc. 2003;84: 1205–1218. doi:10.1175/BAMS-84-9-1205

2. Malhi Y, Wright J. Spatial patterns and recent trends in the climate of tropical rainforest regions. Philos Trans R Soc Lond B Biol Sci. 2004;359: 311–329.

3. Bonan G. Forests and Climate Change: Forcings, Feedbacks, and the Climate Benefits of Forests | Science. Science. 2008;320: 1444–1449.

4. Venter O, Sanderson EW, Magrach A, Allan JR, Beher J, Jones KR, et al. Sixteen years of change in the global terrestrial human footprint and implications for biodiversity conservation. Nat Commun. 2016;7: 12558. doi:10.1038/ncomms12558

5. Jones B, O’Neill BC. Spatially explicit global population scenarios consistent with the Shared Socioeconomic Pathways. Environ Res Lett. 2016;11: 084003. doi:10.1088/1748-9326/11/8/084003

6. Caminade C, Kovats S, Rocklov J, Tompkins AM, Morse AP, Colón-González FJ, et al. Impact of climate change on global malaria distribution. Proc Natl Acad Sci. 2014;111: 3286–3291. doi:10.1073/pnas.1302089111

7. Mordecai EA, Paaijmans KP, Johnson LR, Balzer C, Ben‐Horin T, Moor E de, et al. Optimal temperature for malaria transmission is dramatically lower than previously predicted. Ecol Lett. 2013;16: 22–30. doi:10.1111/ele.12015

8. Guerra CA, Snow RW, Hay SI. A global assessment of closed forests, deforestation and malaria risk. Ann Trop Med Parasitol. 2006;100: 189–204. doi:10.1179/136485906X91512

9. Hansen MC, Potapov PV, Moore R, Hancher M, Turubanova SA, Tyukavina A, et al. High-Resolution Global Maps of 21st-Century Forest Cover Change. Science. 2013;342: 850–853. doi:10.1126/science.1244693

10. Angelsen A, Kaimowitz D. Rethinking the Causes of Deforestation: Lessons from Economic Models. World Bank Res Obs. 1999;14: 73–98. doi:10.1093/wbro/14.1.73

11. Geist HJ, Lambin EF. Proximate Causes and Underlying Driving Forces of Tropical Deforestation: Tropical forests are disappearing as the result of many pressures, both local and regional, acting in various combinations in different geographical locations. BioScience. 2002;52: 143–150. doi:10.1641/0006-3568(2002)052[0143:PCAUDF]2.0.CO;2

12. Vittor AY, Gilman RH, Tielsch J, Glass G, Shields T, Lozano WS, et al. The effect of deforestation on the human-biting rate of Anopheles darlingi, the primary vector of Falciparum malaria in the Peruvian Amazon. Am J Trop Med Hyg. 2006;74: 3–11. doi:10.4269/ajtmh.2006.74.3

13. UN. World Drug Report 2023. In: United Nations : Office on Drugs and Crime [Internet]. 2024 [cited 8 Apr 2026]. Available: //www.unodc.org/unodc/en/data-and-analysis/world-drug-report-2023.html

14. Dávalos LM, Sanchez KM, Armenteras D. Deforestation and Coca Cultivation Rooted in Twentieth-Century Development Projects. BioScience. 2016;66: 974–982. doi:10.1093/biosci/biw118

15. Mejia D, Restrepo P. The economics of the war on illegal drug production and trafficking. J Econ Behav Organ. 2016;126: 255–275. doi:10.1016/j.jebo.2015.11.003

16. Valle D, Clark J. Conservation Efforts May Increase Malaria Burden in the Brazilian Amazon. PloS One. 2013; 1–9. doi:10.1371/journal.pone.0057519

17. Sonter LJ, Ali SH, Watson JEM. Mining and biodiversity: key issues and research needs in conservation science. Proc R Soc B Biol Sci. 2018;285: 20181926. doi:10.1098/rspb.2018.1926

18. Hinton JJ, Veiga MM, Veiga ATC. Clean artisanal gold mining: a utopian approach? J Clean Prod. 2003;11: 99–115. doi:10.1016/S0959-6526(02)00031-8

19. Amaral PST, Garcia KKS, Suárez-Mutis MC, Coelho RR, Galardo AK, Murta F, et al. Malaria in areas under mining activity in the Amazon: A review. Rev Soc Bras Med Trop. 2024;57: e00200. doi:https://doi.org/10.1590/0037-8682-0551-2023

20. Aragão LEOC, Anderson LO, Fonseca MG, Rosan TM, Vedovato LB, Wagner FH, et al. 21st Century drought-related fires counteract the decline of Amazon deforestation carbon emissions. Nat Commun. 2018;9: 536. doi:10.1038/s41467-017-02771-y

21. Bowman DMJS, Balch JK, Artaxo P, Bond WJ, Carlson JM, Cochrane MA, et al. Fire in the Earth System. Science. 2009;324: 481–484. doi:10.1126/science.1163886

22. Hahn MB, Gangnon RE, Barcellos C, Asner GP, Patz JA. Influence of Deforestation, Logging, and Fire on Malaria in the Brazilian Amazon. PLOS ONE. 2014;9: e85725. doi:10.1371/journal.pone.0085725

23. Tusting LS, Willey B, Lucas H, Thompson J, Kafy HT, Smith R, et al. Socioeconomic development as an intervention against malaria: a systematic review and meta-analysis. The Lancet. 2013;382: 963–972. doi:10.1016/S0140-6736(13)60851-X

24. Childs ML, Lyberger K, Harris MJ, Burke M, Mordecai EA. Climate warming is expanding dengue burden in the Americas and Asia. Proc Natl Acad Sci. 2025;122: e2512350122. doi:10.1073/pnas.2512350122

25. Lowe R, Barcellos C, Brasil P, Cruz OG, Honório NA, Kuper H, et al. The Zika Virus Epidemic in Brazil: From Discovery to Future Implications. Int J Environ Res Public Health. 2018;15. Available: https://www.mdpi.com/1660-4601/15/1/96

26. Mordecai EA, Cohen JM, Evans MV, Gudapati P, Johnson LR, Lippi CA, et al. Detecting the impact of temperature on transmission of Zika, dengue, and chikungunya using mechanistic models. PLoS Negl Trop Dis. 2017;11: 1–18. doi:10.1371/journal.pntd.0005568

27. World Bank. Indigenous Latin America in the Twenty-First Century. World Bank; 2015 p. 120. Available: https://documents1.worldbank.org/curated/en/145891467991974540/pdf/Indigenous-Latin-America-in-the-twenty-first-century-the-first-decade.pdf

28. Mulligan K, Dixon J, Joanna Sinn C-L, Elliott SJ. Is dengue a disease of poverty? A systematic review. Pathog Glob Health. 2015;109: 10–18. doi:10.1179/2047773214Y.0000000168

29. Messina JP, Brady OJ, Golding N, Kraemer MUG, Wint GRW, Ray SE, et al. The current and future global distribution and population at risk of dengue. Nat Microbiol. 2019;4: 1508–1515. doi:10.1038/s41564-019-0476-8

30. UN-Habitat, editor. The value of sustainable urbanization. Nairobi, Kenya: UN-Habitat; 2020.

31. Montgomery MR, Hewett PC. Urban poverty and health in developing countries: Household and neighborhood Effects. Demography. 2005;42: 397–425. doi:10.1353/dem.2005.0020

32. WHO. Dengue guidelines, for diagnosis, treatment, prevention and control. In: Dengue guidelines, for diagnosis, treatment, prevention and control [Internet]. 2009 [cited 8 Apr 2026]. Available: https://www.who.int/publications/i/item/9789241547871

33. Alkire S, Santos ME. Measuring Acute Poverty in the Developing World: Robustness and Scope of the Multidimensional Poverty Index. World Dev. 2014;59: 251–274. doi:10.1016/j.worlddev.2014.01.026

34. Valero NNH, Uriarte M. Environmental and socioeconomic risk factors associated with visceral and cutaneous leishmaniasis: a systematic review. Parasitol Res. 2020;119: 365–384. doi:10.1007/s00436-019-06575-5

35. Prestes-Carneiro LE, Daniel LAF, Almeida LC, D’Andrea LZ, Vieira AG, Anjolete IR, et al. Spatiotemporal analysis and environmental risk factors of visceral leishmaniasis in an urban setting in São Paulo State, Brazil. Parasit Vectors. 2019;12: 251. doi:10.1186/s13071-019-3496-6

36. WHO. Leishmaniasis. In: Leishmaniasis [Internet]. 2023 [cited 8 Apr 2026]. Available: https://www.who.int/news-room/fact-sheets/detail/leishmaniasis
