# Supplementary material for: Anthropogenic landscapes and vector-borne disease dynamics: Unveiling the complex interplay between Human Footprint and disease transmission in Colombia
Source: PLOS Glob Public Health. 2026 Jul 9;6(7):e0006801. doi: 10.1371/journal.pgph.0006801 (PMC13349180; doi:10.1371/journal.pgph.0006801)
Supplement: S3 File — ATE estimates for the effect of HFP on excess cases after binarizing HFP at the disease-specific median threshold. (DOCX) [file pgph.0006801.s003.docx]

**Supplement 3**

Average Treatment Effect (ATE) of the effect of Human Footprint (HFP) on excess cases of malaria, dengue, and visceral leishmaniasis, with HFP binarized using the median as the threshold (i.e., malaria: HFP > 6.76 points, dengue: HFP > 6.85 points, visceral leishmaniasis: HFP > 6.80 points). The x-axis depicts the ATE. The blue squares represent the point estimates of the ATE for each disease. The horizontal blue lines correspond to the upper and lower bounds of the associated 95% confidence interval (95% CI). The red line represents the null effect, where the ATE is 0.

**
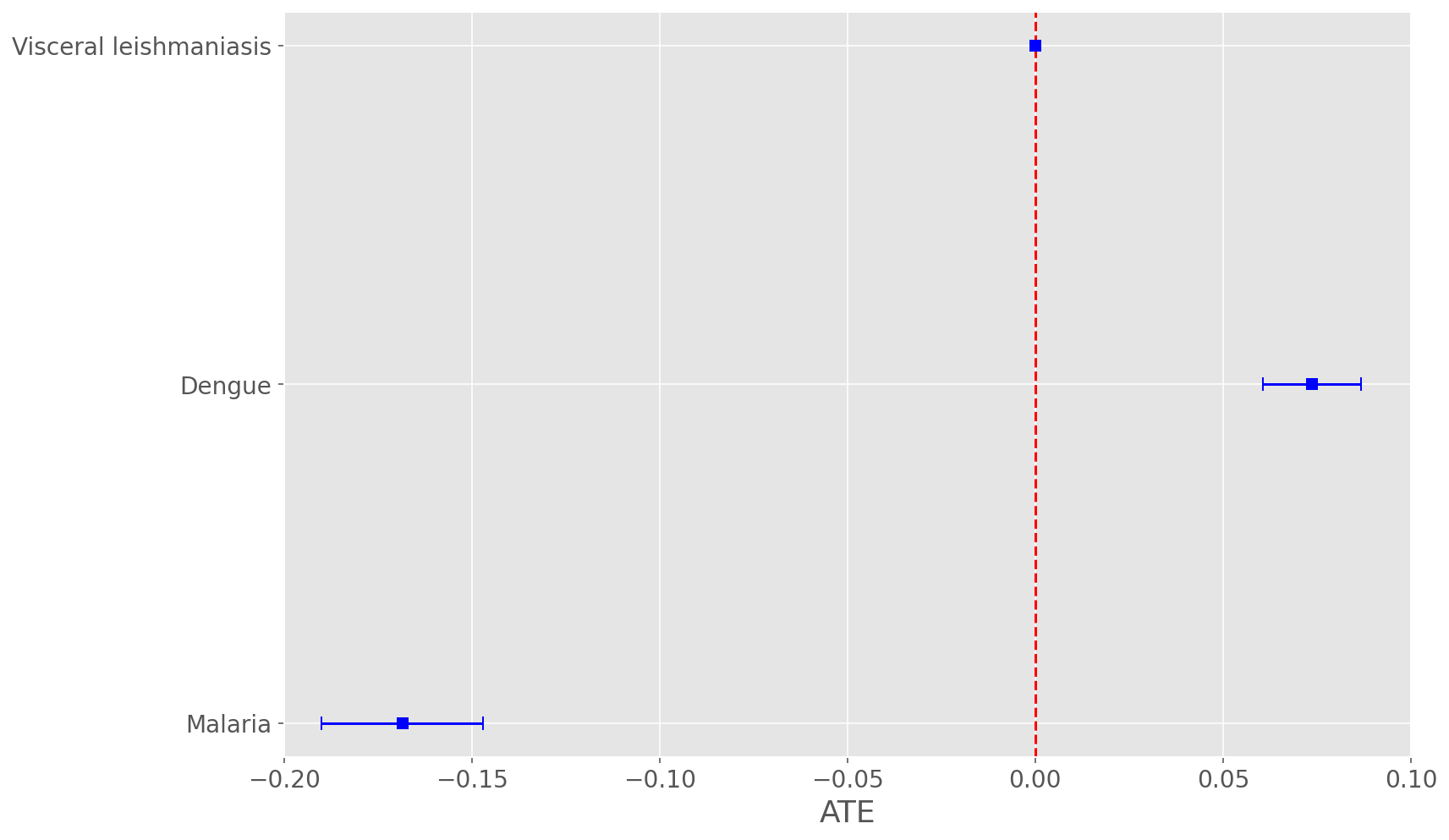
**
