# Supplementary material for: Anthropogenic landscapes and vector-borne disease dynamics: Unveiling the complex interplay between Human Footprint and disease transmission in Colombia
Source: PLOS Glob Public Health. 2026 Jul 9;6(7):e0006801. doi: 10.1371/journal.pgph.0006801 (PMC13349180; doi:10.1371/journal.pgph.0006801)

**Supplement 4**

Juan D. Gutiérrez, Wendy L. Quintero-García, Yanyu Xiao, F. DeWolfe Miller, Diego F. Cuadros

Joint distribution of rainfall and the Human Footprint Index (HFP) for malaria. The figure shows that municipalities exhibiting extreme levels of precipitation are sparsely represented in the sample, resulting in limited empirical support in those regions of the covariate space. This scarcity of observations leads to diminished overlap between exposure groups and, consequently, suggests a partial violation of the positivity (or overlap) assumption in causal inference. Formally, this assumption requires that the probability of receiving each level of the treatment remains strictly bounded away from zero across all strata of the covariate distribution, a condition that appears not to be fully satisfied in the present setting.


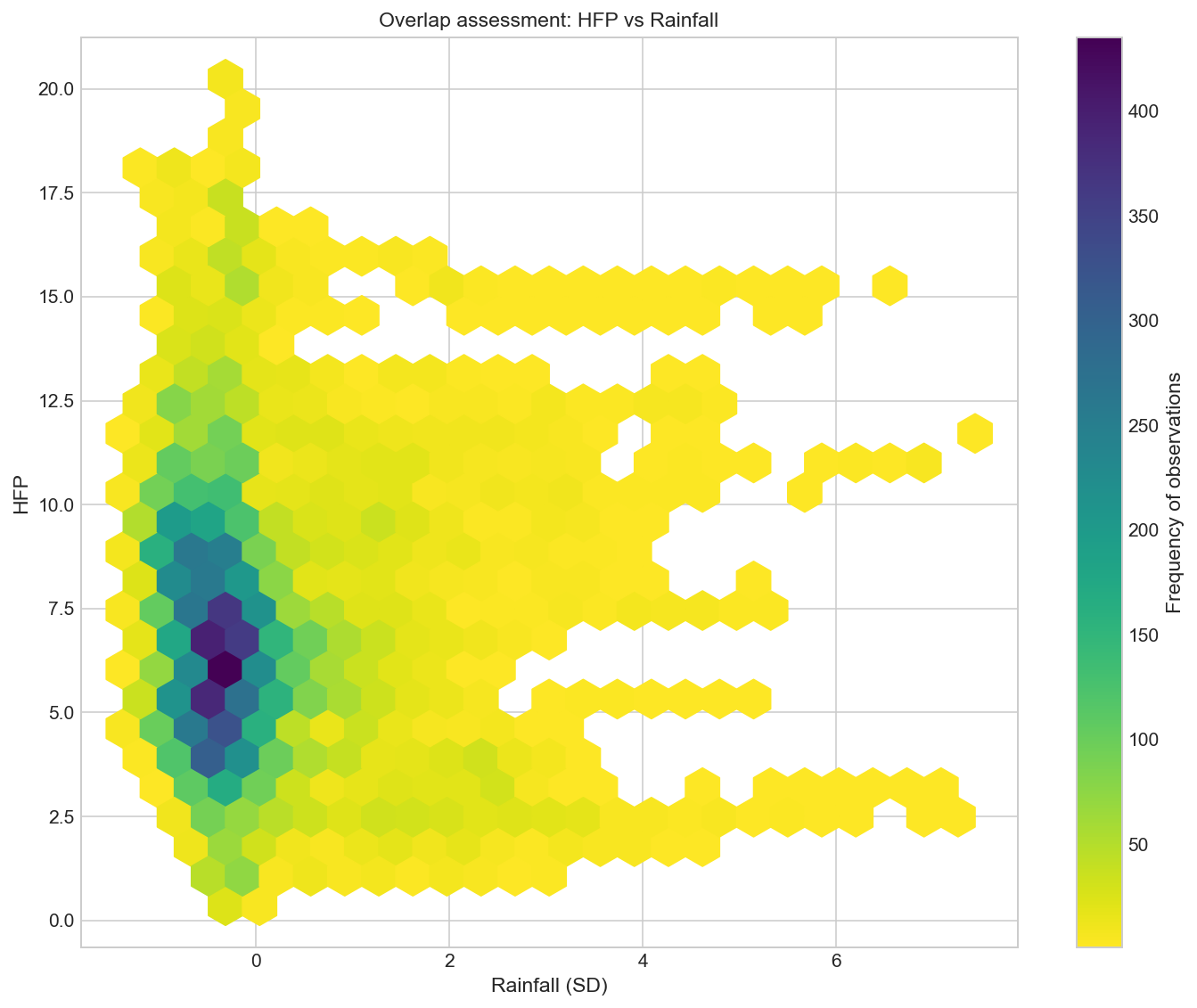

Supplement: S4 File — Joint distribution of rainfall and HFP showing limited empirical support at extreme precipitation values and the resulting positivity concern. (DOCX) [file pgph.0006801.s004.docx]
