# Supplementary material for: Anthropogenic landscapes and vector-borne disease dynamics: Unveiling the complex interplay between Human Footprint and disease transmission in Colombia
Source: PLOS Glob Public Health. 2026 Jul 9;6(7):e0006801. doi: 10.1371/journal.pgph.0006801 (PMC13349180; doi:10.1371/journal.pgph.0006801)
Supplement: Attachment — Submitted filename: Response_Reviewers_041825.pdf [file pgph.0006801.s005.pdf]

Anthropogenic Landscapes and Vector-Borne Disease Dynamics: Unveiling the Complex Interplay between Human Footprint and Disease Transmission in Colombia

Juan D. Gutiérrez, Wendy L. Quintero-García, Yanyu Xiao,
F. DeWolfe Miller, Diego F. Cuadros

RESPONSE TO REVIEWERS' COMMENTS

We appreciate the opportunity to review our work submitted to the journal. We would like to thank the reviewers and the editor for assessing our work and for their valuable feedback and suggestions that have improved our manuscript. Please find below a point-by-point reply that addresses each of the reviewers' comments. We have also incorporated these suggestions in the revised manuscript as noted below.

COMMENTS TO THE AUTHORS

Reviewer #1

The article investigates the impact of the Human footprint (HFP) on vector-disease (malaria, dengue and leishmaniasis) transmission in Colombia, using HFP data (Mu et al., 2022) and annual standardized incidence ratio (SIR) for each disease, computed using the data provided by the epidemiological national surveillance system of Colombia (SIVIGILA), at the municipality level. Several variables (forest coverage, deforestation, illegal mining, urban and rural misery, …) are considered as confounders in the study. For each disease, Directed Acyclic Graphs (DAGs) were used to consider and represent the prior knowledge of causal relationships between HFP, confounders and SIR. Data analysis were performed using a Double machine learning (DML) algorithm. DML was combined with robustness analysis and average and conditional average treatment effects in order to assess the unbiaised impact of the HFP.

Results revealed a significant positive association between HFP and dengue incidence. Conversely, the associations with malaria and visceral leishmaniasis were less pronounced. Results highlight the interplay between environmental and social factors in disease dynamics, especially for dengue disease. The authors conclude on the need for integrated public health interventions that address both ecological and socio-economic dimensions of VBDs, especially in rapidly urbanizing regions.

General considerations

Assessing the capacity of the HFP to predict VBD transmission risk appears relevant and promising. Globally, the article is well structured and clear, with a relatively clear defined methodology. Results are clearly exposed. However, I have some comments about the

actual scope of the article and I consider that some details should be given on the context (disease/vector ecology) and the method (SIVIGILA variables, HFP interpretation and use).

Abstract (and discussion)

- The statement related to the "resilience of malaria and leishmaniasis vectors to human driven habitat modifications" is questionable. For example for malaria, the impacts of such modifications change over time and depend on the intensity and of the permanent or non-permanent character of the modifications: favoring transmission at the time of deforestation and preventing it when urbanization occurs (https://doi.org/10.1073/pnas.0510576103, http://www.malariajournal.com/content/12/1/192)

We would like to sincerely thank Reviewer #1 for their careful reading, constructive feedback, and thoughtful comments, which have helped us strengthen the manuscript. We agree that the ecological response of malaria vectors, particularly Anopheles species, to human-induced environmental changes such as deforestation and urbanization is not unidirectional or universally resilient. As noted by the reviewer, the effect of anthropogenic modifications on malaria transmission varies depending on the intensity, type, and temporal scale of the disturbance. For example, deforestation often creates favorable breeding habitats for *Anopheles darlingi*, while advanced urbanization can lead to the reduction of these habitats.

In response to this comment, we have revised the relevant sentences in both the Abstract and Discussion sections to clarify that the observed lack of significant association in our study reflects a possible buffering effect under the conditions and scale of the HFP used, rather than inherent ecological resilience of the vectors. We now also acknowledge the non-linear and context-dependent nature of malaria transmission in relation to environmental changes. (*abstract and page 11 in the marked document*)

Introduction

- Diseases considered in the study have very different ecology and distribution areas (at least for malaria and dengue) in the study region. This is already well known and should be explicitly mentioned since the beginning. In my opinion, demonstrating these differences can not define the objective of the study, and this can not be considered as the main result of the study as well. However, we can question the fact that the HFP could be a common indicator for these diseases, as it integrates both rural and urban human driven modifications of the ecosystems. Insisting on this objective and the conclusion on the usefulness of the HFP would be more relevant to me.

We thank the reviewer for this insightful comment. We agree that the distinct ecologies and geographic distributions of malaria, dengue, and visceral leishmaniasis in Colombia are well established in the literature and should not, on their own, be framed as the primary contribution of the study. Rather, our central aim was to evaluate whether the HFP index, a

composite measure integrating multiple forms of human pressure across both urban and rural contexts, can serve as a unifying environmental indicator for understanding the dynamics of multiple vector-borne diseases with divergent ecological niches.

In response to the reviewer's suggestion, we have taken the following steps:

- Clarified the framing in the Introduction: We now explicitly state early in the manuscript that while these diseases differ ecologically, our goal is to assess the potential of HFP as a common framework to understand how human-driven ecosystem modifications influence disease transmission.
- Reframed the emphasis in the Discussion and Conclusion: We have revised the language to shift the focus from simply contrasting disease patterns to highlighting the utility and limitations of HFP as a cross-cutting indicator that captures both urban (e.g., dengue) and rural (e.g., malaria, leishmaniasis) anthropogenic impacts. (*pages 3, 12 and 18 in the marked document*)

- More insights have to be given on the vector ecology. For Anopheles, for example, species are not mentioned and the unique bibliographic reference on Anopheles is related to Ghana in Africa. The literature on malaria vectors, especially *An. darlingi*, in South America is rich and should be cited.

We thank the reviewer for highlighting this important gap. The new version of the sentence says: "Malaria, a febrile disease caused by different species of the *Plasmodium* spp., such as *P. vivax*, *P. falciparum*, and *P. malariae*, all of which are present in Colombia. In this country, the parasite is transmitted by *Anopheles* mosquitoes, including *A. darlingi*, *A. nuneztovari*, *A. albimanus*, *A. calderoni*, *A. triannulatus*, and *A. braziliensis*." (*page 3 in the marked document*)

Method

Study design and data sources

- More detailed information should be given on data and variables. The reader should be able to understand and discuss the work without necessarily reading the supplementary materials (which is not the case with the current version of the article).
  Several questions remain unanswered (even after reading the Supplementary file): On malaria data, have plasmodium species been considered jointly or separately?

We appreciate the reviewer's observation and agree that the manuscript should be fully understandable without requiring the reader to consult the supplementary materials. In response to this comment, we have made several updates to the Methods section to provide more detailed and self-contained descriptions of the epidemiological data used. We also modified the sentence to clarify whether parasite species were considered jointly or separately. The new version of the sentence says: "The study integrated

epidemiological data on malaria (All *Plasmodium* species were considered jointly)" (*pages 4 and 5 in the marked document*)

- Have vivax relaps been taken into account, knowing that SIVIGILA system includes a field (nuevo) permitting to detect possible treatment failures and relaps?

We thank the reviewer for raising this important point. We would like to clarify that, beginning in 2014, the SIVIGILA surveillance system in Colombia introduced a specific field to record relapse episodes, particularly for Plasmodium vivax malaria, due to its ability to cause recurrent infections through hypnozoites. These relapses are indeed captured in the system, but they are not classified as new or primary cases. Instead, they are coded as follow-up events and are tied to an earlier notification in the system.

For the purposes of this study, we used only new, confirmed cases of malaria as reported in the SIVIGILA database under the field "caso nuevo." Therefore, relapse events recorded under follow-up categories were not included in the computation of standardized incidence ratios (SIRs) or in the estimation of malaria burden. As a result, our analysis focuses on primary transmission dynamics rather than treatment failures or relapse-related recurrences. (*page 5 in the marked document*)

- What case localization criterion was used (residence, notification or infection municipality)? Infection municipality should be the good choice as the objective is to study the ecology of transmission.

We thank the reviewer for this important observation. We agree that, given the ecological nature of our study and its focus on environmental drivers of disease transmission, the infection municipality represents the most appropriate geographic unit for case attribution. In line with this, we confirm that our analysis used the infection municipality reported in the SIVIGILA system as the localization criterion for all three diseases included in the study.

To improve clarity, we have revised the relevant paragraph in the Methods section to explicitly state this choice. (*page 5 in the marked document*)

- More generally, for reproducibility reason, it would be very useful to add what SIVIGILA fields were considered (at least in the supplementary file).

We have followed the reviewer's suggestion and added to the supplementary file a new paragraph to clarify what SIVIGILA fields were considered. The new paragraph states: "The fields considered at SIVIGILA in our query were: Infection municipality, Date, Age, and Sex. The filters applied were: Cases confirmed by laboratory analysis, Cases dated between 2007 and 2019, Cases that occurred in rural areas (Dispersed rural areas, and rural population centers), and municipal capitals."

- In the same spirit, some details on HFP, given in the supplementary file (and that do not appear in the Table 1), should appear in the main document (at least the 8 variables entering in the HFP computation). This would greatly facilitate the comprehension of the article.

We have followed the reviewer's suggestion and extended the section Study Design and Data Sources and added a paragraph to enumerate the 8 variables used in the HFP estimation and the interpretation of the HFP. The new paragraph states: "Eight variables (built environments, population density, electric infrastructure, croplands, pasturelands, roads, railways, and navigable waterways) are included to estimate the HFP index. The HFP index has a continuous scale from 0 to 50, where 0 corresponds to a pristine ecosystem". (*page 5 in the marked document*)

Data processing

- The last sentence ("Environmental data, ...") should be moved to the previous section (Study design and data sources).

We followed the reviewer's suggestion and moved the sentence to the section Study design and data sources. (*page 5 in the marked document*)

- Machine Learning Techniques and the Implementation of Double Machine Learning. I would have liked more details on the way DAGs were produced. Maybe presenting DAGs before talking about DML (as in the Supplementary file) could help better understand the whole methodology.

We have followed the reviewer's suggestion and renamed the section to Causal Inference and Machine Learning Technique and introduced two paragraphs to give details about causal inference and DAGs.

We mentioned that the potential confounders differ for diseases primarily transmitted in rural (malaria) versus urban (dengue) environments. For visceral leishmaniasis, which exhibits both transmission cycles, we assumed the confounding factors encompass both rural and urban influences. Additionally, we introduced in the DAGs a potential confounder "U" to account for unmeasured variables absent from our dataset, addressing the latent confounding bias in our estimates of HFP's impact on excess disease cases." (*page 6 in the marked document*)

- Considering socioeconomic variables as confounders in the analysis seems pertinent and consistent as they do not enter into the HFP score calculation. However, can we consider that forest coverage and deforestation are indirectly considered in the HFP score through croplands, pastures, roads and railways? If yes, how this can influence data analysis results? (see also comments on Discussion section).

We thank the reviewer for this thoughtful and technically important question. Indeed, some components of the HFP index, such as croplands, pastures, roads, and railways, may reflect land-use changes that are historically associated with forest loss. However, in our study, we carefully considered both the temporal resolution and conceptual distinctions between the cumulative HFP index and dynamic, annually updated variables like forest coverage and deforestation.

To clarify, the HFP index used in our analysis (Mu et al., 2022) integrates multiple human pressures into a cumulative, static score that reflects long-term and persistent anthropogenic modifications. These include transportation networks and major land conversions that often remain unchanged for decades. In contrast, our measures of forest coverage and deforestation are derived from high-resolution, annual satellite data aligned temporally with our disease incidence outcomes. This allows us to capture contemporaneous ecological conditions, such as sudden forest loss, that may not yet be reflected in the cumulative HFP index.

For example, a municipality that experienced deforestation in 2020 will show a reduction in forest coverage and a rise in deforestation that same year, but its HFP value may remain relatively unchanged if infrastructure like roads and croplands had been established years prior. This temporal lag helps justify our inclusion of forest-related variables as independent exposures or confounders, despite partial conceptual overlap with HFP components.

To address this in the manuscript, we revised the Methods and Discussion sections to better explain:

- The temporal distinction between HFP (long-term, cumulative) and forest variables (short-term, annual).
- The analytical rationale for treating these as separate drivers of disease risk.
- The potential ecological implications, such as why *Anopheles* mosquitoes might respond to recent deforestation that is not captured by the HFP score, whereas *Aedes aegypti* responds more to the built environment reflected in the HFP. (*pages 5 and 12 in the marked document*)

Results

- To my knowledge/understanding there is no reference, in the results and discussion sections, to the confounding effects and CATE related to forest coverage and deforestation for malaria and leishmaniasis. Does that mean that there is no confounding effect? This should be stated more clearly.

Thank you for this valuable observation. Forest coverage and deforestation were indeed included as confounders in our DML models for malaria and leishmaniasis. However, because the DML framework is designed to estimate the causal effect of the exposure of interest HFP, these variables were used to adjust the model, not to estimate their individual effects or CATEs.

We did not observe a strong causal effect of HFP on malaria or leishmaniasis after adjusting for forest and socio-economic variables. This suggests that, while forest dynamics may influence transmission, they do not confound the relationship between HFP and these diseases in a way that changes the estimated effect of HFP.

To clarify this distinction, we added a short explanation in the Methods section and revised the paragraph describing how confounders are handled in the DML framework.

Manuscript revisions include:

- Clarifying that forest coverage and deforestation were used as confounders and not analyzed individually.
- Adding a brief explanation in the Methods about the role of confounders in causal inference and how it differs from conventional regression. (*pages 6 and 7 in the marked document*)

We believe these updates make our modeling approach and interpretation more transparent.

Discussion

- It is stated that HFP is built with “urban-focused metrics”. In fact, HFP actually considers both urban and rural human driven modifications. However, it aggregates them in a unique score, and applies higher weights to urban area-related modifications (?). To make it better explain/predict malaria transmission risk, a solution could be to dis-aggregate the score and to only consider rural HFP (subset of the 8 variables of the HFP). Would it be relevant to construct a “rural HFP” (as done in https://hal.science/hal-01425541v1)?

We thank the reviewer for this insightful observation. We agree that the HFP index integrates both urban and rural anthropogenic pressures and that referring to it as “urban-focused” could be misleading. In response, we have revised the wording in the Discussion section to clarify this point.

While disaggregating the HFP into urban and rural components, such as constructing a “rural HFP” using a subset of variables, could indeed offer more targeted insights, this was beyond the scope of our current study. However, we fully agree that such an approach could enhance future research, particularly for diseases like malaria and leishmaniasis, which are more closely linked to rural land-use changes. (*page 13 in the marked document*)

- “[…] agriculture and deforestation, which alter rural landscapes but may not correlate with the urban-focused HFP metrics.”. This does not seem trivial to me i) that agriculture does not correlate with HFP, as croplands and pastures are part of the HFP score; ii) that deforestation does not correlate with HFP, as deforestation

should be correlated with land use changes associated with human settlements and activities.

We thank the reviewer for pointing out this important clarification. We fully agree that croplands and pastures, components of agricultural expansion, as well as deforestation can indeed correlate with the HFP index, given that they reflect land-use changes driven by human activity.

In light of this and in response to the previous related comment, we have revised the paragraph to remove the potentially misleading statement that agriculture and deforestation may not correlate with HFP. The revised text more accurately characterizes the ecological differences in disease transmission dynamics, without implying a weak association between rural land-use changes and HFP. (*page 13 in the marked document*)

- "These differences underscore the importance of tailoring public health interventions to the unique ecological and socio-economic contexts of each disease.": in fact, public health surely already take into account ecological and socio-economic contexts of the diseases. In fact, as previously mentioned above, to my knowledge the differences in the ecology of the considered diseases are already known. The article should better specify in what way (and if possible in what extent) the results of the study could improve such context-specific approach, by shortly describe current public health functioning/actions and the difficulties it encounters in order to efficiently implement them.

We appreciate this thoughtful comment and agree that ecological and socio-economic contexts are already considered in public health strategies for vector-borne diseases in Colombia. However, our study contributes by offering empirical, spatially explicit evidence of how human footprint dynamics modify disease patterns across municipalities. These insights can be used to enhance the precision and responsiveness of current interventions.

To address the reviewer's suggestion, we revised the Discussion to briefly describe the current public health approach in Colombia and highlight how our findings could support more targeted resource allocation and adaptive strategies, particularly in areas experiencing rapid ecological change. (*page 14 in the marked document*)

- "SIVIGILA faces challenges such as potential under-reporting and variability in data quality.". Yes, and this can change over time (due to changes in public policies, professional training, …). Were there significant changes of such biases during the 2007-2019 period?

We thank the reviewer for this important observation. Indeed, the quality and completeness of surveillance data can vary over time due to policy reforms, technological improvements, and workforce training. Between 2007 and 2019, Colombia's SIVIGILA

system underwent several key changes aimed at improving data quality and system performance.

To clarify this, we revised the manuscript to describe how the system evolved during the study period. These updates include: (i) enhancements to notification forms, including the addition of new variables to capture geographic, clinical, and treatment-related details; (ii) improved alignment with international coding standards (e.g., ICD-10); (iii) digitization and integration with other public health platforms; and (iv) strengthened feedback loops to support local decision-making. These advances improved the system's capacity to track diseases like malaria and leishmaniasis, particularly in terms of relapse and follow-up cases.

However, persistent challenges remain, especially in rural or remote areas where electronic notification infrastructure is limited. Underreporting may still occur due to access barriers, asymptomatic cases, and stigma-related hesitancy in reporting. (*page 14 in the marked document*)

- "[...] the variation in disease response to HFP highlights the necessity for differential policy approaches. While dengue requires aggressive urban-focused interventions, diseases like malaria and visceral leishmaniasis call for strategies that address rural and peri-urban settings, where ecological and socio-economic factors play a more significant role in disease transmission.": This is quite obvious to me, and as previously said, public policies should already know that and consider that in its public health interventions.

We appreciate the reviewer's point that differential policy approaches based on disease ecology are already acknowledged in public health planning. In response, we revised the paragraph to shift the emphasis from reiterating general principles to highlighting the specific contributions of our study—namely, the use of empirical, municipality-level evidence on how HFP affects each disease differently. These findings can help sharpen the geographic and contextual targeting of interventions within existing frameworks. (*page 17 in the marked document*)

Reviewer #2:

- This is an interesting paper employing statistical methods in machine learning and causal inference to evaluate the impacts of multi-factorial environmental change (human footprint) on the incidence of vector-borne diseases in Colombia, mining data from public health repositories on malaria, dengue, and visceral leishmaniasis. This combination of methodologies could prove to be a useful framework to evaluating how anthropogenic environmental change influences particular sets of vector-borne and other pathogens. I think the conclusions of the types of human footprint activities that impact visceral leishmaniasis, malarial, and dengue are pretty well characterized already in the literature, therefore the study results are not

surprising. Nonetheless, the authors' methodology provides a transferable framework to explore lesser-known emerging pathogens in future studies.

We sincerely thank the reviewer for their thoughtful and encouraging feedback. We greatly appreciate the recognition of our methodological approach and agree that, while some ecological associations are already known, our contribution lies in offering a replicable and data-driven causal inference framework for evaluating the impacts of complex anthropogenic pressures on disease transmission. We are particularly encouraged by the reviewer's suggestion that this framework may be valuable for studying emerging or lesser-known pathogens, and we fully agree with this perspective. Thank you again for your supportive comments, which help reinforce the broader relevance of our study.

Minor comments:

Introduction:

- I suggest deleting the 'advertisement' words in the manuscript-the manuscript is robust and does not need that, as these methods have been used in ecology and epidemiology. For instance:
  No need to say 'breaks new ground' just mention the objective of the study
  Delete the word 'novel' before 'analytical framework' and also anywhere in the manuscript if repeated.

We have followed the reviewer's suggestion and removed advertisement words, which are also value judgments, and unnecessary in scientific writing.

Methods:

- Delete 'providing a holistic view of how anthropogenic changes impact public health' as this is clear in the approach.

Done

- Replace 'intricate dynamics' with 'socioecological dynamics'

Done

- Please explain in detail what the 'comprehensive ecological analysis' entailed- Can you explain what that means in terms of methodology so it could be replicated if someone decides to replicate this approach? or is the ecological analysis what you explain below- if so please summarize it after 'comprehensive ecological analysis.

We thank the reviewer for pointing out the ambiguity in our original phrasing. To improve clarity and replicability, we removed the expression "comprehensive ecological analysis"

and replaced it with a more precise description of the study design. The revised text now clearly states that the study was conducted at the municipal (ecological) level, using retrospective data from SIVIGILA and other national datasets. (*page 4 in the marked document*)

- Delete 'In this study'

Done

- Table 1 provides a good summary of the variables used in the study. Why did you only use 2018 data for home deprivation, population density, and urban and rural misery. I still am not clear of the index calculated for urban and rural indices -how this is calculated or measured exactly. Is there a misery index range of scales, or just binary (yes-misery no-no misery?).

We thank the reviewer for these thoughtful questions. We have updated the Study Design and Data Sources section to clarify both the temporal availability of the socio-economic data and the definition of the misery indices.

First, we used 2018 data for variables such as home deprivation, urban and rural misery, ethnic composition, and population density because these were obtained from the most recent national census conducted by the National Department of Statistics (DANE). Similarly, the urban dimension variable was only available for the year 2015, as measured by the National Department of Planning.

Second, we clarified that the misery index is not binary. According to DANE, a household is considered in misery if it experiences monetary poverty and/or multidimensional deprivation (e.g., lack of access to health, education, or adequate living conditions). In our analysis, the index represents the percentage of households in misery per municipality, making it a continuous variable ranging from 0 to 100%. (*page 5 in the marked document*)

- Can you explicitly explain here in the methods not just the summary table how you quantified the HFP (human footprint?).

Thank you for this important clarification request. In response, we revised the Methods section to explicitly describe how the Human Footprint (HFP) index was calculated, beyond its summary in the table. (*page 5 in the marked document*)

Discussion:

- The study limitations were very well described.
  Authors state: "While dengue requires aggressive urban-focused interventions, diseases like malaria and visceral leishmaniasis call for strategies that address rural

and peri-urban settings, where ecological and socio-economic factors play a more significant role in disease transmission." Aren't these facts already well-known in vector-borne disease control?

We appreciate the reviewer's point that the general ecological differences between dengue, malaria, and visceral leishmaniasis are well documented and already guide vector-borne disease (VBD) control efforts. Our intention was not to reiterate established knowledge, but to contribute spatially explicit and empirically derived evidence showing how human footprint dynamics, as a composite measure of anthropogenic pressures, differentially influence these diseases within the Colombian context.

In response, we revised the paragraph to shift emphasis toward the study's contribution to refining current strategies and improving targeted resource allocation, rather than stating known differences in intervention approaches. (*page 17 in the marked document*)

Conclusion:

- For the sentence that begins 'in contrast', change 'moderated' to 'moderate'

Done

- Delete 'innovative'

Done
