# Supplementary material for: Anthropogenic landscapes and vector-borne disease dynamics: Unveiling the complex interplay between Human Footprint and disease transmission in Colombia
Source: PLOS Glob Public Health. 2026 Jul 9;6(7):e0006801. doi: 10.1371/journal.pgph.0006801 (PMC13349180; doi:10.1371/journal.pgph.0006801)
Supplement: Attachment — Submitted filename: Response_reviewers_110325.pdf [file pgph.0006801.s007.pdf]

Anthropogenic Landscapes and Vector-Borne Disease Dynamics: Unveiling the Complex Interplay between Human Footprint and Disease Transmission in Colombia

Juan D. Gutiérrez, Wendy L. Quintero-García, Yanyu Xiao,
F. DeWolfe Miller, Diego F. Cuadros

RESPONSE TO REVIEWERS' COMMENTS

We appreciate the opportunity to review our work submitted to the journal. We would like to thank the reviewers and the editor for assessing our work and for their valuable feedback and suggestions that have improved our manuscript. Please find below a point-by-point reply that addresses each of the reviewers' comments. We have also incorporated these suggestions in the revised manuscript as noted below.

COMMENTS TO THE AUTHORS

Reviewer #3

Dear Reviewer, please note that, following the suggestions of Reviewer No. 4, we have modified the research approach and methodology. However, you will find our responses to your comments in the following lines.

This study has evaluated the causal relationships between the Human Footprint Index and the excess cases of three vector-borne diseases in Colombia: dengue, malaria, and visceral leishmaniasis. Dengue showed the highest positive association, followed by malaria, while no significant association was observed for visceral leishmaniasis.

The combination of DAGs with machine learning makes this a methodologically robust study. This approach also has the potential to be replicated for other environmentally-sensitive infectious diseases and regions, taking into account the local epidemiological context. The authors have clearly addressed the main comments from a previous round of reviews (which I was not part of), and the manuscript is now clearer and close to publication quality.

That said, I missed a deeper explanation of the local and regional eco-epidemiology of visceral leishmaniasis (VL). While the manuscript provides species-level detail for malaria vectors and parasites (Anopheles and Plasmodium), the same level of taxonomic and ecological detail is missing for Leishmania spp. and their sandfly vectors (Lutzomyia spp.). Colombia has a wide diversity of Lutzomyia and Leishmania species, each with distinct ecological preferences: some more adapted to rural environments, others to peri-urban or domestic habitats. VL has been closely linked to urbanization throughout Latin America,

primarily due to the peri-urban distribution and specific habitat and feeding preferences of its principal vector, Lutzomyia longipalpis.

The comment is accepted. Species-level details are provided for the parasites and vectors of visceral leishmaniasis in Colombia, as well as their ecoepidemiology (lines 87-95 in the marked document).

Furthermore, the methods section should clarify how leishmaniasis data from SIVIGILA were processed. Unlike the clear description provided for malaria (e.g., aggregation across Plasmodium species), it is not specified whether SIVIGILA distinguishes between cutaneous and visceral leishmaniasis, and whether only confirmed VL cases were analyzed. If both forms were included by chance, even if that is a limitation of the main data source, there is a risk that the causal signals are diluted, thus reducing the statistical significance of the results.

The comment has been accepted; we have added a new sentence to clarify this point in the Methods section (lines 135-138 in the marked document)

Including these methodological details and improving the discussion of how case reporting and difference between VL and CL may influence the relationship with HFP would add depth to the study and improve the interpretability of the results.

The comment is accepted. As mentioned in the previous comments, a new sentence has been included to clarify the difference between VL and CL (lines 135-138 in the marked document). In addition, clarifications have been made in the Discussion section on lines 484-496 in the marked document.

Reviewer #4:

Dear Reviewer, please note that, following your suggestions, we have modified the research approach and methodology. In particular, we have included rainfall and temperature as effect modifiers, and we have re-estimated the ATEs and CATEs for this new scope of our study. This new scope implies that we modified several parts of the Methods and Results sections. However, you will find our responses to your comments in the following lines.

Anthropogenic Landscapes and Vector-Borne Disease Dynamics: Unveiling the Complex Interplay between Human Footprint and Disease Transmission in Colombia

Summary: I really enjoyed reading this manuscript and found the use of a double machine learning approach for causal inference exciting. I have two major critiques:

1. Methods: The methods are not explained thoroughly enough for readers who are unfamiliar with them. I noted several places where the explanation could be elaborated. Since PLoS is not restrictive with word count, adding more detail should not be an issue.

We thank the reviewer for this point, and we have changed the Methods section to clarify our procedures. You can see the details in the answer to the respective comments below.

2. Methods: VL cases are often very zero inflated. How is this accounted for in the modeling framework?

We thank the reviewer for this important point. Visceral leishmaniasis (VL) notifications are indeed sparse in many municipalities and can produce a strongly zero-inflated distribution of raw case counts; we considered this carefully when building our causal framework, and we report below how the current analysis addresses sparse/zero counts, and the remaining limitations.

- How zero counts were handled in the present analysis (and rationale).
    - Our primary outcome is municipal excess cases defined from the standardized incidence ratio (SIR): we dichotomized the outcome as excess = 1 if SIR > 1, and 0 otherwise. Using a binary excess-case outcome shifts the inference target from predicting raw counts to estimating the causal effect of HFP on the probability of observing higher-than-expected incidence; this reduces sensitivity of the causal estimator to raw count zero-inflation and aligns with our inferential goal of isolating an exposure effect on the occurrence (excess) rather than on point prediction of counts. (see Supplementary Methods and main Methods).
    - We implemented a sparse Double Machine Learning (DML) approach with flexible nuisance models (extreme gradient boosting and polynomial featurization) so that the first-stage models for treatment and outcome can flexibly capture complex patterns in the confounders and reduce model misspecification that could otherwise amplify biases arising from sparse outcomes. This modelling choice was made specifically to prioritize valid causal effect estimation (ATE and CATE) under potentially complex, non-linear confounding structures.
- Limitations that remain / evidence of potential bias.
    - The surveillance dataset contains only 294 confirmed VL cases for 2007–2019 at the municipal level. This small number of events reduces statistical power and increases sampling variability; we explicitly acknowledge this limitation in the manuscript (see Discussion section).

- Our refutation checks indicated some remaining bias under data perturbations (Table 2: replacing a random subset produced statistically different estimates), which suggests the VL result should be interpreted cautiously (see Discussion section).
-

3. Ecology of visceral leishmaniasis: The ecology of visceral leishmaniasis is inaccurately described throughout the study, which affects the choice of confounders and the interpretation of results. VL is transmitted primarily by a single sandfly species, uses dogs as reservoir hosts, and is caused by a single Leishmania species. It is typically not associated with forest environments.

The comment is accepted. Species-level details are provided for the parasites and vectors of visceral leishmaniasis in Colombia, along with their ecoepidemiology, as shown in lines 87-95 in the marked document.

Intro:

• Pg 3, p1: Are there certain key malaria vectors that are more important to the ecology discussed in the manuscript than others?

The comment is accepted. The text in lines 68-79, mentions the key malaria vectors. Additionally, emphasis is placed on the ecoepidemiology of the vector in the discussion section, lines 401-412 in the marked document.

Pg 3, p1: Not all sandfly vectors are nocturnal, and not all are adapted to peridomestic environments (see comment below).

Comment accepted, we modified the sentence on lines 85-87. Additionally, we included a new paragraph in the Discussion section emphasizing the vector behavior (lines 428-437 in the marked document).

Pg 3, p1: Visceral leishmaniasis is caused by one specific Leishmania species (Leishmania infantum) and has primarily one sandfly vector (Lu. longipalpis). The ecology of VL is very different from that of CL and MCL, so they should not be grouped together when discussing the ecology and transmission cycles of the diseases.

The comment is accepted. Species-level details are provided for the parasites and vectors of visceral leishmaniasis in Colombia, as well as their ecoepidemiology in lines 87-95 in the marked document.

• Overall:

I think it is important to include more background on the three diseases as it applies to their specific relationships with different landscape types and human pressures, making sure to adequately cite background literature. For instance, there is a lot of literature discussing the nuances of malaria and deforestation that might be critical for understanding the results and for better contextualizing the manuscript's novel contribution to our understanding of malaria.

The comment is accepted. More information about the three diseases and their relationship to the human footprint is provided in the Discussion section, lines 401-437 in the marked document.

Methods:

• Table 1: I really like Table 1; it does a great job of describing the variables used.

• Page 6, p3: I don't think visceral leishmaniasis is typically classified as "urban and rural"; it is usually described as "urban and peri-urban." Please make sure you are not confusing the VL transmission cycle with those of CL and MCL.

Comment accepted. This sentence was modified in lines 187-190 in the marked document.

• Pg 5. Causal inference and machine learning technique: Did you test variables for multicollinearity? Even though gradient boosting models (GBMs) can handle higher multicollinearity than classical statistical models, it can still be problematic. Strong correlations ($\rho > 0.7$) can increase variance, reduce interpretability, and contribute to overfitting if redundant predictors are included.

We thank the reviewer for this important remark about multicollinearity. We fully agree that, even when using flexible learners such as gradient boosting machines, strong predictor correlations (e.g., $\rho > 0.7$) may increase variance, reduce interpretability, and complicate inference if handled improperly. We therefore took a principled, causal-inference–focused approach rather than applying ad-hoc variable exclusion. Specifically:

- Adjustment set determined by causal reasoning (DAGs): Following practice in epidemiologic causal inference, our adjustment sets were selected from Directed Acyclic Graphs (DAGs) that encode domain knowledge about the relationships among HFP, the outcome, and potential confounders. We used DAGs to identify the appropriate adjustment set variables for each disease, and we avoided removing variables from those sets solely because of pairwise correlation, since doing so can induce confounding bias (e.g., by leaving a backdoor path unblocked) (see Methods

and Supplementary Materials). In that sense, from a causal inference perspective, the unique case that justifies removing a covariate is when the ρ value = 1. This is because the goal of causal inference is to estimate the effect of the exposure on the outcome by addressing different sources of bias, including confounding bias, rather than predicting the response variable from independent variables, typically using regression models.

- Estimator choice to reduce bias while handling many correlated covariates: We estimated effects with a sparse Double Machine Learning (DML) framework that separates nuisance estimation from treatment-effect estimation. The nuisance (regression and propensity) models used gradient-boosting learners with polynomial featurization and a sparse-DML architecture, which (a) reduces bias from model mis-specification and (b) stabilizes estimation in high-dimensional settings where predictors may be correlated. We also standardized and, where noted, binarized covariates to aid model convergence and numerical stability. These design choices were selected so that we do not need to drop causally important covariates merely because they are correlated (see Machine learning implementation and Data Transformation sections in the Supplementary Materials).
- On variable exclusion and causal bias: As the reviewer correctly notes, removing confounders from an adjustment set because they correlate highly with other covariates can open biasing paths or leave residual confounding. In line with this, we did not use correlation-based deletion rules to decide the adjustment sets. Instead, causal necessity (per the DAGs) determined inclusion; statistical regularization in the nuisance models (sparse DML, tree-based learners, polynomial featurizer) controlled for redundancy and overfitting in the nuisance estimation stage.
-

• Pg 6, p3: How is U estimated? Is it based on spatio-temporal autocorrelation? Is it more like a random intercept matrix?

We thank the reviewer for this significant point, unusual in scientific literature. Actually, U cannot be estimated because the U node in the DAGs is a theoretical construct used to transparently communicate potential limitations due to unmeasured confounding. Our statistical approach operationalizes this by performing refutation tests to quantify the robustness of our conclusions to unobserved confounders (see Methods and Supplementary Materials: Refutation analysis). We appreciate the opportunity to clarify this distinction.

• Pg 6, p4: Did you train a model for each fold, or for three combinations of two folds (with the third as the hold-out)? Was this step used to test out-of-sample predictive performance,

or to identify variation in model outcomes depending on the partition of data? Why did you choose a learning rate of 0.01 and not tune this hyperparameter? Please clarify in the text.

Thank you for your question regarding our cross-validation approach. We appreciate the opportunity to clarify this methodological detail.

First, note that we have modified the code to answer the new comments of the reviewers. In that sense, for our analysis, we employed standard k-fold cross-validation (with k=5 folds) as implemented in the EconML package's DML estimator. Specifically, we set cv=5 in the model initialization parameters, which instructs the algorithm to:

- Train separate models for each fold: The estimator automatically creates 5 non-overlapping folds of the data and trains 5 distinct models (one for each fold). For each fold, the model is trained on 4/5 of the data and validated on the remaining 1/5.
- Purpose of cross-validation: This approach was used primarily to improve causal estimation robustness rather than for out-of-sample predictive performance evaluation. In the context of double machine learning (DML) frameworks like EconML:
    - The cross-validation helps prevent overfitting when estimating the nuisance functions (models for outcome and treatment assignment)
    - It ensures that the causal effect estimates are not overly dependent on any particular data partition
    - The final ATE (Average Treatment Effect) is computed as an average across all folds, providing more stable and less biased estimates
- Not for predictive performance: While our models incorporate machine learning components, our primary goal was causal inference, not prediction. The cross-validation was not used to evaluate predictive accuracy but rather as a built-in mechanism of the DML framework to obtain consistent causal estimates by addressing the "double robustness" property.

This is a standard implementation pattern in the EconML library for causal inference, where cross-validation serves to separate the estimation of nuisance parameters from the causal effect estimation, thereby reducing overfitting bias. The documentation of EconML recommends this approach specifically for obtaining reliable causal estimates.

Additionally, we have included a new paragraph in the Discussion section to recognize the limitations of our research with respect to the optimization of hyperparameters (lines 568-583 in the marked document).

• Pg 6, p4: Did you use spatio-temporal cross-validation? If so, please explain. If not, please justify why spatio-temporal cross-validation does not need to be used for this methodology, given the expectation of spatial and temporal autocorrelation.

We appreciate the reviewer's insightful comment regarding spatio-temporal cross-validation. In our study, we employed a DML framework with XGBoost, using a five-fold cross-validation procedure to ensure the robustness of causal effect estimation. However, we acknowledge that our cross-validation strategy did not explicitly incorporate spatio-temporal blocking to account for spatial and temporal autocorrelation.

This choice was primarily motivated by the design of our analysis. Our unit of inference was the municipality-year, and the goal of the DML framework was to obtain unbiased causal estimates of the effect of the HFP on vector-borne disease outcomes, rather than to maximize predictive performance. While spatio-temporal cross-validation can be valuable in prediction-focused studies, by avoiding overly optimistic estimates of predictive accuracy due to spatial and temporal dependence, our emphasis on causal estimation, supported by directed acyclic graphs (DAGs) and robustness checks, reduced the need for such an approach.

Nevertheless, we recognize that spatio-temporal dependence can still influence model estimation. By not employing spatio-temporal blocking, there remains the possibility that some residual autocorrelation may have affected our standard error estimates or contributed to remaining bias. As we discussed in the manuscript, this is a limitation of our study (lines 568-583). Incorporating spatio-temporal fixed effects and explicitly adopting spatio-temporal cross-validation schemes would likely further strengthen the robustness of causal inference, especially in contexts where unobserved spatial and temporal heterogeneity plays a significant role.

• Pg 7, Robustness Analysis: Please explain what each of these tests are. Additionally, clarify what you mean by "latent bias in the estimate."

We accepted the comment, and we have included lines 231-270 in the marked document to explain this point. Additionally, note that for clarity of the reader, we changed latent bias to remaining bias.

• Pg 8, p1: Why was HFP binarized? What do you mean by this? For readers not familiar with the analysis, it sounds as though the variable was transformed into 0 vs 1, which would lose a lot of information and defeat the purpose of using HFP as a variable.

As mentioned above, we have modified the code to address the reviewers' new comments. The current version of our study treats the HFP as a continuous variable, but in standard

deviation units to facilitate the convergence of the machine learning model (see the Methods and Results sections).

Results

• Pg 8, p3: How do the ATE estimates translate into excess disease cases?

We appreciate the reviewer's request for clarification on the interpretation of our ATE estimates. We recognize this is a critical point for understanding the public health implications of our findings.

Our ATE estimates represent the average change in the probability of observing excess disease cases (defined as SIR > 1) associated with a one standard deviation (sd) increase in the HFP index.

Interpretation of ATE values:

- Dengue (ATE = 0.035, 95% CI: 0.017–0.054): One sd increase in HFP (equivalent to 3.23 HFP units) is associated with a 3.5 percentage point increase in the probability that a municipality will experience excess dengue cases. For example, if a municipality initially has a 30% probability of excess cases, this increase in HFP would raise that probability to 33.5%.
- Malaria (ATE = -0.099, 95% CI: -0.155 to -0.083): One sd increase in HFP (3.33 units) is associated with a 9.9 percentage point decrease in the probability of excess malaria cases, reflecting probably the rural-adapted ecology of *Anopheles* vectors.
- Visceral leishmaniasis (ATE = 0.007, 95% CI: 0.000–0.014): One sd increase in HFP (3.24 units) is associated with a 0.7 percentage point increase in the probability of excess cases, a modest but statistically significant effect.

Translation to population-level impact:

To contextualize these probability changes at the population level, note that Colombia has 1,122 municipalities. An ATE of 0.035 for dengue suggests that approximately 39 additional municipalities (1,122 × 0.035 ~ 39) would be expected to transition from non-excess to excess dengue burden with each sd increase in HFP, assuming uniform distribution of baseline risk.

We have added clarifying text in the Results section (lines 298-303) to make this interpretation explicit and have expanded the Discussion to address the public health significance of these probability changes in the context of Colombia's disease burden and municipal heterogeneity.

• Pg 8, p3: VL transmission is typically stochastic and sparse. Could this influence the model's ability to detect an HFP signal? How is zero-inflation/overdispersion accounted for in the model?

As we mentioned in a previous answer, VL notifications are indeed sparse in many municipalities and can produce a strongly zero-inflated distribution of raw case counts. We considered this carefully when building our causal framework, and we noted in the previous answer: (1) how the current analysis addresses sparse/zero counts, and (2) the remaining limitations.

• Pg 8, p2–3: I think these qualitative results could serve as the opening of the Results section to better contextualize the system. Please place the maps of HFP and case distributions in the main text so they are easier to reference.

We accepted the comment, and we moved these qualitative results to the Results section.

• Pg 8–9: The ATE estimates for dengue have very wide confidence intervals. Is this expected for this type of analysis? What are the implications of such wide CIs for interpreting the results?

We accepted the comment and have included a new paragraph in the Results (lines 322-325 in the marked document) explaining that the variables rural misery, urban misery, and rainfall correspond to right-skewed distributions, characterized by a low number of observations at the upper tail. This results in very wide confidence intervals on the right side of the CATE estimations.

• Pg 8–9 (bottom and top paragraphs): I am not sure what the numbers in parentheses after "municipalities with HFP larger than the median" refer to.

Please note that in the current version of the study, the HFP is a continuous variable, but transformed to standard deviation units to facilitate the convergence of the machine learning models. We now estimate the effect of an increase of one standard deviation unit of HFP. In this regard, we have modified the Results section (lines 290-303 in the marked document).

• Pg 9, p2: I really like the CATE analysis. Have you examined it for climate? Perhaps dengue is more sensitive to HFP in areas at the edge of climate suitability, where HFP might help create small pockets of more favorable transmission conditions in regions that would otherwise be too cool or dry.

In the current version of the study, we estimated the CATE conditioned on rainfall and temperature (see the Results section). Additionally, note that to estimate the CATE for a specific effect modifier, we held the other effect modifier variables constant at their respective mean values, with the goal of isolating the marginal effect of each effect modifier variable, as we mentioned in lines 304-325 in the marked document.

• Pg 9, p3: I am not sure what this means. Please elaborate on how this was estimated and why it is important.

We have included a set of new paragraphs to explain how to interpret the results of refutation tests and their implications for our study (lines 326-351 in the marked document).

• Overall: Please redefine ATE and CATE in the Discussion so the reader doesn't need to search back through the paper to recall their definitions.

We accepted the comment, and we included a paragraph to restate the key causal estimands (i.e., ATE and CATE) (lines 358-371 in the marked document).

Discusión:

• Pg 10: "In fact, in a global perspective, countries that were not considered endemic for certain diseases a few years ago now report infections caused by vectors locally": please provide a citation.

Comment accepted. The following quote is added: Chala, B., & Hamde, F. (2021). Emerging and Re-emerging Vector-Borne Infectious Diseases and the Challenges for Control: A Review. *Frontiers in public health*, *9*, 715759. https://doi.org/10.3389/fpubh.2021.715759

• Pg 10, p3: If you do not statistically investigate how climate might influence these relationships and the resulting lack of statistical significance, it would be interesting to include a discussion on how incorporating a climate interaction could mediate the HFP × misery interaction.

As mentioned above, the new version of the study includes rainfall and temperature as variables that modify the effect of HFP on excess cases of the diseases. We also estimated the CATE conditioned on both variables.

Pg 10, p4 – Pg 11, p1: This is a lot of discussion for a result that was not significant. Consider moving this paragraph above the prior paragraph (Pg 10, p3) to first set the reader up with why misery should be a significant interactor, then present your result, and finally explain why the relationship was not significant.

In the current version of the study, we obtained different CATE results conditioned on misery, and we revised the writing of the Discussion section regarding this point (lines 438-449 in the marked document).

• Pg 11, p3: This information is not relevant for visceral leishmaniasis, which is primarily caused by a single species, transmitted by one or two vector species, and typically maintained by domestic or built-environment–adapted reservoir hosts.

The comment has been accepted, and we have removed this information in the new version.

• Pg 12, p3: While VL occurs in rural and peri-urban fringes, both the sandflies and reservoir hosts are not forest-associated. Deforestation and logging should not directly influence VL; instead, they may influence transmission indirectly, on a time lag, as cleared land is converted to other land uses more closely related to VL ecology.

The comment is accepted. The paragraph was modified in lines 385-390 in the marked document.

• Pg 13, p3: The Skinner paper also investigates each of these diseases individually. Therefore, the sentence "our research narrows its focus concentrating on dengue, malaria and visceral leishmaniasis" does not make sense in relation to how it builds on Skinner. The exciting addition of this manuscript is that it uses a causal inference approach and includes socio-economic factors.

Comment accepted; the clarification about the Skinner paper was introduced in lines 513-524 in the marked document.

NOTE: The manuscript did not include line numbers. Please include them in the future to make it easier to give feedback.

The comment is accepted. Line numbers are included in the manuscript.
