# Supplementary material for: Anthropogenic landscapes and vector-borne disease dynamics: Unveiling the complex interplay between Human Footprint and disease transmission in Colombia
Source: PLOS Glob Public Health. 2026 Jul 9;6(7):e0006801. doi: 10.1371/journal.pgph.0006801 (PMC13349180; doi:10.1371/journal.pgph.0006801)
Supplement: Attachment — Submitted filename: Response to Reviewers.pdf [file pgph.0006801.s010.pdf]

Anthropogenic Landscapes and Vector-Borne Disease Dynamics: Unveiling the Complex Interplay between Human Footprint and Disease Transmission in Colombia

Juan D. Gutiérrez, Wendy L. Quintero-García, Yanyu Xiao,
F. DeWolfe Miller, Diego F. Cuadros

RESPONSE TO REVIEWERS' COMMENTS

We appreciate the opportunity to review our work submitted to the journal. We would like to thank the reviewers and the editor for assessing our work and for their valuable feedback and suggestions that have improved our manuscript. Please find below a point-by-point reply that addresses each of the reviewers' comments. We have also incorporated these suggestions in the revised manuscript as noted below.

COMMENTS TO THE AUTHORS

Reviewer #2:

I think nearly all comments were very thoroughly and adequately addressed, there is only one thing that could be clarified a bit more prior to publication- specifically, whether the size and direction of the treatment effect stay basically the same, and whether turning HFP into a binary variable changes interpretation or understanding of the results.

R: We appreciate the comment and we have included the results of turning HFP into a binary variable (lines 427 - 433 and supplementary material 3).

Reviewer #6:

This study uses machine learning models to investigate the effect of the human footprint (HFP) on the transmission of vector-borne diseases (VBDs) in Colombia. They start proposing causal models (DAGs) for the relationship between HFP and excess VBD, considering climate and sociodemographic variables as confounders. Overall, the study is relevant and innovative as it applies modern machine learning approaches to estimate causal effects. My comments are mainly related to improve the clarity of the model and the scope of the discussion: see the uploaded file.

1. Outcome. The outcome of the study is defined "excess disease cases". To compute it, the authors calculated the annual age- Standardized Incidence Ratio (SIR) and Excess cases were defined "as a binary variable (1 when SIR > 1, 0 otherwise)". I am not convinced this is an adequate definition of excess cases. These diseases have very different levels of incidence, and can be very low in some places and small numbers can distort ratios. Should you calculate confidence intervals and only assign as excess if they do not include SIR =1?

R: We appreciate the comment and agree with it. In this regard, we have added new paragraphs to the Discussion section to acknowledge this point (lines 634 – 656).

2. Line 170. the sentence "using the backdoor path," it is not very precise; my sugestion is **"using the Backdoor Criterion to identify and block backdoor paths."**

R: We accept the comment and we change the sentence (Supplementary material 1, Pg. 3 p. 3)

3. DAGs. Line 182 reads "For each disease, we constructed a Directed Acyclic Graph (DAG) to illustrate our existing knowledge regarding the interrelations among variables in the causal model and to clearly outline each causal assumption (Fig. 1)." In general lines, the DAGs seem adequate, but the authors do not provide any justification for the chosen structure and variables. **DAGs were** built based on previous **knowledge** and I would expect much more information on the evidence for each element of the model, including references. I recommed to include a table (supplement) for each disease indicating the rationale for each variable and connection, including references.

In Fig 1, it is not clear what means the boxes – we know that deforestation and misery and fire are all causal linked. This means that an acyclic graph is a simplification. Also, since these are infectious diseases, previous incidence affects current incidence. I mean, the current DAG is OK, but has limitations (as all models), and these limitations need to be identified and acknowledged.

R: We appreciate the comment and have modified Figure 1 to display all causal links in the DAGs. Additionally, in Supplementary Material 1, we have included supporting evidence from the scientific literature for each element of the DAGs.

In line 226, what you mean by "validated DAGs"? Where is the validation?

R: We accept the comment and we removed this sentence.

Line 229. "Comprehensive understanding" is overstated. No single study provides a "comprehensive" understanding of complex systems like disease dynamics.

R: We accept the comment and we removed this sentence.

4. DML. Line 198. While the paragraph is good, DML is a magic bullet, as it still relies on assumptions: DML assumes that all relevant confounders are measured and included in the dataset. 1) If there is a hidden confounder (e.g., a cultural factor affecting both HFP and disease that was not measured), DML cannot fix that bias. 2) There must be enough variation in the data. For example, you need some areas with high HFP and some with low HFP that look similar in other ways. So we need to have some description of this cross-variability. If HFP is always high in poor areas and always low in rich areas, DML cannot separate the effect of HFP from the effect of poverty. 3) DML cannot fix bias from variables that were never measured (unobserved confounding).

R: We appreciate the comment and agree with it. In this regard, we have added a new paragraph to the Methods section to explicitly enumerate the assumptions in our causal estimation (lines

232 – 241). Additionally, we have included several sentences in the Discussion section highlighting the possible violation to these assumptions (lines 434 – 676).

These XGBoost parameter choices are atypical. Were 2,500 trees and 0.0001 chosen via cross-validation, or were they fixed arbitrarily?

R: We appreciate the comment and we implemented a procedure to systematically identify the best-performing configuration for each of the three diseases analyzed (lines 173 – 205, and supplementary material 1 and 2).

Line 208. Usually, model selection (choosing hyperparameters) is based on minimizing the prediction error (e.g., Mean Squared Error) of the nuisance models (the XGBoost parts). Why did you choose CI? What is the consequence of using CI instead of MSE?

R: See previous answer.

5. Results and Discussion. Line 385 “In contrast to dengue, the transmission dynamics of malaria and visceral leishmaniasis are more related to non-urban socio-economic conditions. For instance, the spread of malaria and visceral leishmaniasis is often associated with rural or peri-urban activities, including land-use changes such as deforestation and logging”. These conclusions are derived from the embedded structure of the DAGs, that differed between diseases exactly by the incusion of rural or urban variables. To make the above claims, the authors should have used the same DAG for all the diseases and checked if the fitted models differed and indicated.

R: We appreciate the comment and have completely revised the Discussion section to address each disease separately, thereby improving its clarity and strengthening its focus on the debate surrounding the signal observed in the average and heterogeneous effect of HFP. We have also included a discussion of the limitations of HFP as a valid exposure variable in the context of causal estimation (lines 434 – 676).

This is a overall fragility of the discussion. Since the models are different for the three diseases, the best practice would be to discuss them separately.

R: See previous answer.

Line 400. “Our results of the ATE suggest a preference of malaria vector species for natural environments, land when these conditions are transformed by anthropogenic activities, it leads to a reduction in the probability of observing excess malaria cases in Colombian municipalities. However, the dynamics of malaria in sylvatic regions… “ This sentence and many other sentences in the discussion can be improved. The model is not suggesting that Anopheles prefer natural environments, this was already known and expected (that’s why this link is in the DAG). I suggest to focus on the estimated effects of covariates on excess cases, the nonlinear shapes of the funcional relationships, the thresholds when a variable shifts from protection to risk. This is the rich aspect of the study.

R: Please, see previous answer.

Moreover. I think the discussion can be much improved and strong if it focus on the identification of the most vulnerable conditions for each disease and how HFP can be used to measure it. For example, areas that combine excess cases and high socio-environm vulnerability and (low? High?) HFP could be highlighted as priority areas for surveillance and health assistance.

R: Please, see previous answer.
