# Supplementary material for: Anthropogenic landscapes and vector-borne disease dynamics: Unveiling the complex interplay between Human Footprint and disease transmission in Colombia
Source: PLOS Glob Public Health. 2026 Jul 9;6(7):e0006801. doi: 10.1371/journal.pgph.0006801 (PMC13349180; doi:10.1371/journal.pgph.0006801)
Supplement: Attachment — Submitted filename: Anthropogenic Landscapes and Vector.docx [file pgph.0006801.s006.docx]

*Anthropogenic Landscapes and Vector-Borne Disease Dynamics: Unveiling the Complex Interplay between Human Footprint and Disease Transmission in Colombia*

Summary: I really enjoyed reading this manuscript and found the use of a double machine learning approach for causal inference exciting. I have two major critiques:

1. **Methods:** The methods are not explained thoroughly enough for readers who are unfamiliar with them. I noted several places where the explanation could be elaborated. Since PLoS is not restrictive with word count, adding more detail should not be an issue.
2. **Methods:** VL cases are often very zero inflated. How is this accounted for in the modeling framework?
3. **Ecology of visceral leishmaniasis:** The ecology of visceral leishmaniasis is inaccurately described throughout the study, which affects the choice of confounders and the interpretation of results. VL is transmitted primarily by a single sandfly species, uses dogs as reservoir hosts, and is caused by a single Leishmania species. It is typically not associated with forest environments.

Intro:

- **Pg 3, p1:** Are there certain key malaria vectors that are more important to the ecology discussed in the manuscript than others?
- **Pg 3, p1:** Not all sandfly vectors are nocturnal, and not all are adapted to peridomestic environments (see comment below).
- **Pg 3, p1:** Visceral leishmaniasis is caused by one specific Leishmania species (Leishmania infantum) and has primarily one sandfly vector (Lu. longipalpis). The ecology of VL is very different from that of CL and MCL, so they should not be grouped together when discussing the ecology and transmission cycles of the diseases.
- **Overall:**
  I think it is important to include more background on the three diseases as it applies to their specific relationships with different landscape types and human pressures, making sure to adequately cite background literature. For instance, there is a lot of literature discussing the nuances of malaria and deforestation that might be critical for understanding the results and for better contextualizing the manuscript’s novel contribution to our understanding of malaria.

**Methods:**

- **Table 1:** I really like Table 1; it does a great job of describing the variables used.
- **Page 6, p3:** I don’t think visceral leishmaniasis is typically classified as “urban and rural”; it is usually described as “urban and peri-urban.” Please make sure you are not confusing the VL transmission cycle with those of CL and MCL.
- **Pg 5, Causal inference and machine learning technique:** Did you test variables for multicollinearity? Even though gradient boosting models (GBMs) can handle higher multicollinearity than classical statistical models, it can still be problematic. Strong correlations (ρ > 0.7) can increase variance, reduce interpretability, and contribute to overfitting if redundant predictors are included.
- **Pg 6, p3:** How is U estimated? Is it based on spatio-temporal autocorrelation? Is it more like a random intercept matrix?
- **Pg 6, p4:** Did you train a model for each fold, or for three combinations of two folds (with the third as the hold-out)? Was this step used to test out-of-sample predictive performance, or to identify variation in model outcomes depending on the partition of data? Why did you choose a learning rate of 0.01 and not tune this hyperparameter? Please clarify in the text.
- **Pg 6, p4:** Did you use spatio-temporal cross-validation? If so, please explain. If not, please justify why spatio-temporal cross-validation does not need to be used for this methodology, given the expectation of spatial and temporal autocorrelation.
- **Pg 7, Robustness Analysis:** Please explain what each of these tests are. Additionally, clarify what you mean by “latent bias in the estimate.”
- **Pg 8, p1:** Why was HFP binarized? What do you mean by this? For readers not familiar with the analysis, it sounds as though the variable was transformed into 0 vs 1, which would lose a lot of information and defeat the purpose of using HFP as a variable.

**Results**

- **Pg 8, p3:** How do the ATE estimates translate into excess disease cases?
- **Pg 8, p3:** VL transmission is typically stochastic and sparse. Could this influence the model’s ability to detect an HFP signal? How is zero-inflation/overdispersion accounted for in the model?
- **Pg 8, p2–3:** I think these qualitative results could serve as the opening of the Results section to better contextualize the system. Please place the maps of HFP and case distributions in the main text so they are easier to reference.
- **Pg 8–9:** The ATE estimates for dengue have very wide confidence intervals. Is this expected for this type of analysis? What are the implications of such wide CIs for interpreting the results?
- **Pg 8–9 (bottom and top paragraphs):** I am not sure what the numbers in parentheses after “municipalities with HFP larger than the median” refer to.
- **Pg 9, p2:** I really like the CATE analysis. Have you examined it for climate? Perhaps dengue is more sensitive to HFP in areas at the edge of climate suitability, where HFP might help create small pockets of more favorable transmission conditions in regions that would otherwise be too cool or dry.
- **Pg 9, p3:** I am not sure what this means. Please elaborate on how this was estimated and why it is important.
- **Overall:** Please redefine ATE and CATE in the Discussion so the reader doesn’t need to search back through the paper to recall their definitions.

**Discussion:**

- **Pg 10:** “In fact, in a global perspective, countries that were not considered endemic for certain diseases a few years ago now report infections caused by vectors locally”: please provide a citation.
- **Pg 10, p3:** If you do not statistically investigate how climate might influence these relationships and the resulting lack of statistical significance, it would be interesting to include a discussion on how incorporating a climate interaction could mediate the HFP × misery interaction.
- **Pg 10, p4 – Pg 11, p1:** This is a lot of discussion for a result that was not significant. Consider moving this paragraph above the prior paragraph (Pg 10, p3) to first set the reader up with why misery should be a significant interactor, then present your result, and finally explain why the relationship was not significant.
- **Pg 11, p3:** This information is not relevant for visceral leishmaniasis, which is primarily caused by a single species, transmitted by one or two vector species, and typically maintained by domestic or built-environment–adapted reservoir hosts.
- **Pg 12, p3:** While VL occurs in rural and peri-urban fringes, both the sandflies and reservoir hosts are not forest-associated. Deforestation and logging should not directly influence VL; instead, they may influence transmission indirectly, on a time lag, as cleared land is converted to other land uses more closely related to VL ecology.
- **Pg 13, p3:** The Skinner paper also investigates each of these diseases individually. Therefore, the sentence “our research narrows its focus concentrating on dengue, malaria and visceral leishmaniasis” does not make sense in relation to how it builds on Skinner. The exciting addition of this manuscript is that it uses a causal inference approach and includes socio-economic factors.
