# Supplementary material for: Anthropogenic landscapes and vector-borne disease dynamics: Unveiling the complex interplay between Human Footprint and disease transmission in Colombia
Source: PLOS Glob Public Health. 2026 Jul 9;6(7):e0006801. doi: 10.1371/journal.pgph.0006801 (PMC13349180; doi:10.1371/journal.pgph.0006801)
Supplement: Attachment — Submitted filename: PLos.odt [file pgph.0006801.s009.odt]

Anthropogenic Landscapes and Vector-Borne Disease Dynamics: Unveiling the
Complex Interplay between Human Footprint and Disease Transmission in Colombia


This study uses machine learning models to investigate the effect of the human footprint (HFP) on the transmission of vector-borne diseases (VBDs) in Colombia. They start proposing causal models (DAGs) for the relationship between HFP and excess VBD, considering climate and sociodemograíc variables as confounders. Overall, the study is relevant and innovative as it applies modern machine learning approaches to estimate causal effects. My comments are mainly related to improve the clarity of the model and the scope of the discussion:

1. Outcome. The outcome of the study is defined “excess disease cases”.  To compute it, the authors calculated the annual age- Standardized Incidence Ratio (SIR)  and Excess cases were defined “as a binary variable (1 when SIR > 1, 0 otherwise)”. I am not convinced this is an adequate definition of excess cases. These diseases have very different levels of incidence, and can be very low in some places and small numbers can distort ratios. Should you calculate confidence intervals and only assign as excess if they do not include SIR =1?
 
2. Line 170.  the sentence "using the backdoor path," it is not very precise; my sugestion is "using the Backdoor Criterion to identify and block backdoor paths."

3. DAGs.  Line 182 reads “For each disease, we constructed a Directed Acyclic Graph (DAG) to illustrate our existing knowledge regarding the interrelations among variables in the causal model and to clearly 184outline each causal assumption (Fig. 1).” In general lines, the DAGs seem adequate, but the authors do not provide any justification for the chosen structure and variables. DAGs were built based on previous knowledge and I would expect much more information on the evidence for each element of the model, including references. I recommed to include a table (supplement) for each disease indicating the rationale for each variable and connection, including references.
In Fig 1, it is not clear what means the boxes – we know that deforestation and misery and fire are all causal linked. This means that an acyclic graph is a simplification. Also, since these are infectious diseases, previous incidence affects current incidence. I mean, the current DAG is OK, but has limitations (as all models), and these limitations need to be identified and acknowledged.  

In line 226, what you mean by “validated DAGs”? Where is the validation? 

Line 229. "Comprehensive understanding" is overstated. No single study provides a "comprehensive" understanding of complex systems like disease dynamics. 

4. DML. Line 198. While the paragraph is good, DML is a magic bullet, as it still relies on assumptions:  DML assumes that all relevant confounders are measured and included in the dataset. 1) If there is a hidden confounder (e.g., a cultural factor affecting both HFP and disease that was not measured), DML cannot fix that bias. 2) There must be enough variation in the data. For example, you need some areas with high HFP and some with low HFP that look similar in other ways. So we need to have some description of this cross-variability. If HFP is always high in poor areas and always low in rich areas, DML cannot separate the effect of HFP from the effect of poverty. 3) DML cannot fix bias from variables that were never measured (unobserved confounding).  

These XGBoost parameter choices are atypical. Were 2,500 trees and 0.0001 chosen via cross-validation, or were they fixed arbitrarily? 

Line 208. Usually, model selection (choosing hyperparameters) is based on minimizing the prediction error (e.g., Mean Squared Error) of the nuisance models (the XGBoost parts).  Why did you choose CI? What is the consequence of using CI instead of MSE?


5. Results and Discussion

Line 385 “In contrast to dengue, the transmission dynamics of malaria and visceral leishmaniasis are more related to non-urban socio-economic conditions. For instance, the spread of malaria and visceral leishmaniasis is often associated with rural or peri-urban activities, including land-use changes such as deforestation and logging”. These conclusions are derived from the embedded structure of  the DAGs, that differed between diseases exactly by the incusion of rural or urban variables. To make the above claims, the authors should have used the same DAG for all the diseases and checked if the fitted models differed and indicated.

This is a overall fragility of the discussion. Since the models are different for the three diseases, the best practice would be to discuss them separately. 


Line 400. “Our results of the ATE suggest a preference of malaria vector species for natural environments, land when these conditions are transformed by anthropogenic activities, it leads to a reduction in4 the probability of observing excess malaria cases in Colombian municipalities. However, the dynamics of malaria in sylvatic regions… “  This sentence and many other sentences in the discussion can be improved. The model is not suggesting that Anopheles prefer natural environments, this was already known and expected (that’s why this link is in the DAG). I suggest to focus on the estimated effects of covariates on excess cases, the nonlinear shapes of the funcional relationships, the thresholds when a variable shifts from protection to risk. This is the rich aspect of the study. 

Moreover. I think the discussion can be much improved and strong if it focus on the identification of the most vulnerable conditions for each disease and how HFP can be used to measure it. For example, areas that combine excess cases and high socio-environm vulnerability and (low? High?) HFP could be highlighted as priority areas for surveillance and health assistance.
