# Supplementary material for: Anthropogenic landscapes and vector-borne disease dynamics: Unveiling the complex interplay between Human Footprint and disease transmission in Colombia
Source: PLOS Glob Public Health. 2026 Jul 9;6(7):e0006801. doi: 10.1371/journal.pgph.0006801 (PMC13349180; doi:10.1371/journal.pgph.0006801)
Supplement: Peer review history

# Peer review: Decision Letter 0

**DOI:** 10.1371/journal.pgph.0006801.r001

**Authors:** Mathieu Nacher

18 Feb 2025

PGPH-D-24-02579

Anthropogenic Landscapes and Vector-Borne Disease Dynamics: Unveiling the Complex Interplay between Human Footprint and Disease Transmission in Colombia

PLOS Global Public Health

Dear Dr. Cuadros,

Thank you for submitting your manuscript to PLOS Global Public Health. After careful consideration, we feel that it has merit but does not fully meet PLOS Global Public Health’s publication criteria as it currently stands. Therefore, we invite you to submit a revised version of the manuscript that addresses the points raised during the review process.

Please submit your revised manuscript by Apr 04 2025 11:59PM. If you will need more time than this to complete your revisions, please reply to this message or contact the journal office at globalpubhealth@plos.org. When you're ready to submit your revision, log on to https://www.editorialmanager.com/pgph/ and select the 'Submissions Needing Revision' folder to locate your manuscript file.

Please include the following items when submitting your revised manuscript:

- A rebuttal letter that responds to each point raised by the editor and reviewer(s). You should upload this letter as a separate file labeled 'Response to Reviewers'.
- A marked-up copy of your manuscript that highlights changes made to the original version. You should upload this as a separate file labeled 'Revised Manuscript with Track Changes'.
- An unmarked version of your revised paper without tracked changes. You should upload this as a separate file labeled 'Manuscript'.

Guidelines for resubmitting your figure files are available below the reviewer comments at the end of this letter.

We look forward to receiving your revised manuscript.

Kind regards,

Mathieu Nacher

Academic Editor

PLOS Global Public Health

Journal Requirements:

1. Please note that PLOS Global Public Health has specific guidelines on code sharing for submissions in which author-generated code underpins the findings in the manuscript. In these cases, all author-generated code must be made available without restrictions upon publication of the work. Please review our guidelines at https://journals.plos.org/globalpublichealth/s/materials-and-software-sharing#loc-sharing-code and ensure that your code is shared in a way that follows best practice and facilitates reproducibility and reuse.

Additional Editor Comments (if provided):

[Note: HTML markup is below. Please do not edit.]

Reviewers' comments:

Reviewer's Responses to Questions

**Comments to the Author**

1. Does this manuscript meet PLOS Global Public Health’s publication criteria? Is the manuscript technically sound, and do the data support the conclusions? The manuscript must describe methodologically and ethically rigorous research with conclusions that are appropriately drawn based on the data presented.

Reviewer #1: Partly

Reviewer #2: Yes

**********

2. Has the statistical analysis been performed appropriately and rigorously?

Reviewer #1: Yes

Reviewer #2: Yes

**********

3. Have the authors made all data underlying the findings in their manuscript fully available (please refer to the Data Availability Statement at the start of the manuscript PDF file)?

The PLOS Data policy requires authors to make all data underlying the findings described in their manuscript fully available without restriction, with rare exception. The data should be provided as part of the manuscript or its supporting information, or deposited to a public repository. For example, in addition to summary statistics, the data points behind means, medians and variance measures should be available. If there are restrictions on publicly sharing data—e.g. participant privacy or use of data from a third party—those must be specified.

Reviewer #1: Yes

Reviewer #2: No

**********

4. Is the manuscript presented in an intelligible fashion and written in standard English?

PLOS Global Public Health does not copyedit accepted manuscripts, so the language in submitted articles must be clear, correct, and unambiguous. Any typographical or grammatical errors should be corrected at revision, so please note any specific errors here.

Reviewer #1: Yes

Reviewer #2: Yes

**********

5. Review Comments to the Author

Please use the space provided to explain your answers to the questions above. You may also include additional comments for the author, including concerns about dual publication, research ethics, or publication ethics. (Please upload your review as an attachment if it exceeds 20,000 characters)

Reviewer #1: The article investigates the impact of the Human footprint (HFP) on vector-disease (malaria, dengue and leishmaniasis) transmission in Colombia, using HFP data (Mu et al., 2022) and annual standardized incidence ratio (SIR) for each disease, computed using the data provided by the epidemiological national surveillance system of Colombia (SIVIGILA), at the municipality level. Several variables (forest coverage, deforestation, illegal mining, urban and rural misery, …) are considered as confounders in the study. For each disease, Directed Acyclic Graphs (DAGs) were used to consider and represent the prior knowledge of causal relationships between HFP, confounders and SIR. Data analysis were performed using a Double machine learning (DML) algorithm. DML was combined with robustness analysis and average and conditional average treatment effects in order to assess the unbiaised impact of the HFP. Results revealed a significant

positive association between HFP and dengue incidence. Conversely, the associations with malaria and visceral leishmaniasis were less pronounced. Results highlight the interplay between environmental and social factors in disease dynamics, especially for dengue disease. The authors conclude on the need for integrated public health interventions that address both ecological and socio-economic dimensions of VBDs, especially in rapidly urbanizing regions.

General considerations

Assessing the capacity of the HFP to predict VBD transmission risk appears relevant and promising. Globally, the article is well structured and clear, with a relatively clear defined methodology. Results are clearly exposed. However, I have some comments about the actual scope of the article and I consider that some details should be given on the context (disease/vector ecology) and the method (SIVIGILA variables, HFP interpretation and use).

Abstract (and discussion)

The statement related to the "resilience of malaria and leishmaniasis vectors to human driven habitat modifications" is questionable. For example for malaria, the impacts of such modifications change over time and depend on the intensity and of the permanent or non-permanent character of the modifications: favoring transmission at the time of deforestation and preventing it when urbanization ocurre (https://doi.org/10.1073/pnas.0510576103, http://www.malariajournal.com/content/12/1/192)

Introduction

Diseases considered in the study have very different ecology and distribution areas (at least for malaria and dengue) in the study region. This is already well known and should be explicitly mentioned since the beginning. In my opinion, demonstrating these differences can not define the objective of the study, and this can not be considered as the main result of the study as well. However, we can question the fact that the HFP could be a common indicator for these diseases, as it integrates both rural and urban human driven modifications of the ecosystems. Insisting on this objective and the conclusion on the usefulness of the HFP would be more relevant to me.

More insights have to be given on the vector ecology. For Anopheles for example, species are not mentioned and the unique bibliographic reference on Anopheles in related to Ghana in Africa. The literature on malaria vectors, especially An. darlingi, in South America is rich and should be cited.

Method

Study design and data sources

More detailed information should be given on data and variables. The reader should be able to understand and discuss the work without necessarily read the supplementary materials (which is not the case with the current version of the article).

Several questions remain unanswered (even after reading the Supplementary file):

On malaria data, have plasmodium species been considered jointly or separately?

Have vivax relaps been taken into account, knowing that SIVIGILA system includes a field (nuevo) permitting to detect possible treatment failures and relaps?

What case localization criterion was used (residence, notification or infection municipality)? Infection municipality should be the good choice as the objective is to study the ecology of transmission.

More generally, for reproducibility reason, it would be very useful to add what SIVIGILA fields were considered (at least in the supplementary file).

In the same spirit, some details on HFP, given in the supplementary file (and that do not appear in the Table 1), should appear in the main document (at least the 8 variables entering in the HFP computation). This would greatly facilitate the comprehension of the article.

Data processing

The last sentence (“Environmental data, ...”) should be moved to the previous section (Study design and data sources).

Machine Learning Techniques and the Implementation of Double Machine Learning

I would have liked more details on the way DAGs were produced. Maybe presenting DAGs before talking about DML (as in the Supplementary file) could help better understand the whole methodology.

Considering socioeconomic variables as confounders in the analysis seems pertinent and consistent as they do not enter in the HFP score calculation. However, can we consider that forest coverage and deforestation are indirectly considered in the HFP score through croplands, pastures, roads and railways? If yes, how this can influence data analysis results? (see also comments on Discussion section).

Results

To my knowledge/understanding there is no reference, in the results and discussion sections, to the confounding effects and CATE related to forest coverage and deforestation for malaria and leishmaniasis. Does that mean that there is no confounding effect? This should be stated more clearly.

Discussion

It is stated that HFP is built with “urban-focused metrics”. In fact, HFP actually considers both urban and rural human driven modifications. However, it aggregates them in a unique score, and applies higher weights to urban area-related modifications (?). To make it better explain/predict malaria transmission risk, a solution could be to dis-aggregate the score and to only consider rural HFP (subset of the 8 variables of the HFP). Would it be relevant to construct a “rural HFP” (as done in https://hal.science/hal-01425541v1)?

“[…] agriculture and deforestation, which alter rural landscapes but may not correlate with the urban-focused HFP metrics.”. This does not seem trivial to me i) that agriculture does not correlate with HFP, as croplands and pastures are part of the HFP score ; ii) that deforestation does not correlate with HFP, as deforestation should be correlated with land use changes associated with human settlements and activities.

“These differences underscore the importance of tailoring public health interventions to the unique ecological and socio-economic contexts of each disease.”: in fact, public health surely already take into account ecological and socio-economic contexts of the diseases. In fact, as previously mentioned above, to my knowledge the differences in the ecology of the considered diseases are already known. The article should better specify in what way (and if possible in what extent) the results of the study could improve such context-specific approach, by shortly describe current public health functioning/actions and the difficulties it encounters in order to efficiently implement them.

“SIVIGILA faces challenges such as potential under-reporting and variability in data quality.”. Yes, and this can change over time (due to changes in public policies, professional training, …). Were there significant changes of such biases during the 2007-2019 period?

“[...] the variation in disease response to HFP highlights the necessity for differential policy approaches. While dengue requires aggressive urban-focused interventions, diseases like malaria and visceral leishmaniasis call for strategies that address rural and peri-urban settings, where ecological and socio-economic factors play a more significant role in disease transmission.”: This is quite obvious to me, and as previously said, public policies should already know that and consider that in its public health interventions.

Reviewer #2: This is an interesting paper employing statistical methods in machine learning and causal inference to evaluate the impacts of multi-factorial environmental change (human footprint) on the incidence of vector-borne diseases in Colombia, mining data from public health repositories on malaria, dengue, and visceral leishmaniasis. This combination of methodologies could prove to be a useful framework to evaluating how anthropogenic environmental change influences particular sets of vector-borne and other pathogens. I think the conclusions of the types of human footprint activities that impact visceral leishmaniasis, malarial, and dengue are pretty well characterized already in the literature, therefore the study results are not surprising. Nonetheless, the authors' methodology provides a transferable framework to explore lesser-known emerging pathogens in future studies.

Minor comments:

Introduction:

I suggest deleting the 'advertisement' words in the manuscript-the manuscript is robust and does not need that, as these methods have been used in ecology and epidemiology. For instance:

a) no need to say 'breaks new ground' just mention the objective of the study

b) delete the word 'novel' before 'analytical framework' and also anywhere in the manuscript if repeated.

Methods:

delete 'providing a holistic view of how anthropogenic changes impact public health' as this is clear in the approach.

replace 'intricate dynamics' with 'socioecological dynamics'

please explain in detail what the 'comprehensive ecological analysis' entailed- Can you explain what that means in terms of methodology so it could be replicated if someone decides to replicate this approach? or is the ecological analysis what you explain below- if so please summarize it after 'comprehensive ecological analysis.

delete 'In this study'

Table 1 provides a good summary of the variables used in the study. Why did you only use 2018 data for home deprivation, population density, and urban and rural misery. I still am not clear of the index calculated for urban and rural indices -how this is calculated or measured exactly. Is there a misery index range of scales, or just binary (yes-misery no-no misery?).

Can you explicitly explain here in the methods not just the summary table how you quantified the HFP (human footprint?).

Discussion:

The study limitations were very well described.

Authors state: "While dengue requires aggressive urban-focused interventions, diseases like malaria and visceral leishmaniasis call for strategies that address rural and peri-urban settings, where ecological and socio-economic factors play a more significant role in disease transmission." Aren't these facts already well-known in vector-borne disease control?

Conclusion:

For the sentence that begins 'in contrast', change 'moderated' to 'moderate'

delete 'innovative'

**********

6. PLOS authors have the option to publish the peer review history of their article (what does this mean?). If published, this will include your full peer review and any attached files.

**Do you want your identity to be public for this peer review?**  If you choose “no”, your identity will remain anonymous but your review may still be made public.

For information about this choice, including consent withdrawal, please see our Privacy Policy.

Reviewer #1: No

Reviewer #2: No

**********

[NOTE: If reviewer comments were submitted as an attachment file, they will be attached to this email and accessible via the submission site. Please log into your account, locate the manuscript record, and check for the action link "View Attachments". If this link does not appear, there are no attachment files.]

While revising your submission, please upload your figure files to the Preflight Analysis and Conversion Engine (PACE) digital diagnostic tool, https://pacev2.apexcovantage.com/. PACE helps ensure that figures meet PLOS requirements. To use PACE, you must first register as a user. Registration is free. Then, login and navigate to the UPLOAD tab, where you will find detailed instructions on how to use the tool. If you encounter any issues or have any questions when using PACE, please email PLOS at figures@plos.org. Please note that Supporting Information files do not need this step.

# Author comment: Author response to Decision Letter 1

**DOI:** 10.1371/journal.pgph.0006801.r002

18 Apr 2025

Attachment Submitted filename: Response_Reviewers_041825.pdf

# Peer review: Decision Letter 1

**DOI:** 10.1371/journal.pgph.0006801.r003

**Authors:** Srinivasa Rao Mutheneni

2 Sep 2025

PGPH-D-24-02579R1

Anthropogenic Landscapes and Vector-Borne Disease Dynamics: Unveiling the Complex Interplay between Human Footprint and Disease Transmission in Colombia

PLOS Global Public Health

Dear Dr. Diego F Cuadros,

Thank you for submitting your manuscript to PLOS Global Public Health. After careful consideration, we feel that it has merit but does not fully meet PLOS Global Public Health’s publication criteria as it currently stands. Therefore, we invite you to submit a revised version of the manuscript that addresses the points raised during the review process.

Please submit your revised manuscript by 03.10.2025. If you will need more time than this to complete your revisions, please reply to this message or contact the journal office at globalpubhealth@plos.org. When you're ready to submit your revision, log on to https://www.editorialmanager.com/pgph/ and select the 'Submissions Needing Revision' folder to locate your manuscript file.

Please include the following items when submitting your revised manuscript:

- A rebuttal letter that responds to each point raised by the editor and reviewer(s). You should upload this letter as a separate file labeled 'Response to Reviewers'.
- A marked-up copy of your manuscript that highlights changes made to the original version. You should upload this as a separate file labeled 'Revised Manuscript with Track Changes'.
- An unmarked version of your revised paper without tracked changes. You should upload this as a separate file labeled 'Manuscript'.

Guidelines for resubmitting your figure files are available below the reviewer comments at the end of this letter.

We look forward to receiving your revised manuscript.

Kind regards,

Srinivasa Rao Mutheneni, PhD

Academic Editor

PLOS Global Public Health

Journal Requirements:

If the reviewer comments include a recommendation to cite specific previously published works, please review and evaluate these publications to determine whether they are relevant and should be cited. There is no requirement to cite these works unless the editor has indicated otherwise.

Additional Editor Comments (if provided):

Reviewer #3:

Reviewer #4:

[Note: HTML markup is below. Please do not edit.]

Reviewers' comments:

Reviewer's Responses to Questions

**Comments to the Author**

1. If the authors have adequately addressed your comments raised in a previous round of review and you feel that this manuscript is now acceptable for publication, you may indicate that here to bypass the “Comments to the Author” section, enter your conflict of interest statement in the “Confidential to Editor” section, and submit your "Accept" recommendation.

Reviewer #3: (No Response)

Reviewer #4: (No Response)

**********

2. Does this manuscript meet PLOS Global Public Health’s publication criteria? Is the manuscript technically sound, and do the data support the conclusions? The manuscript must describe methodologically and ethically rigorous research with conclusions that are appropriately drawn based on the data presented.

Reviewer #3: Yes

Reviewer #4: Partly

**********

3. Has the statistical analysis been performed appropriately and rigorously?

Reviewer #3: Yes

Reviewer #4: Yes

**********

4. Have the authors made all data underlying the findings in their manuscript fully available (please refer to the Data Availability Statement at the start of the manuscript PDF file)?

The PLOS Data policy requires authors to make all data underlying the findings described in their manuscript fully available without restriction, with rare exception. The data should be provided as part of the manuscript or its supporting information, or deposited to a public repository. For example, in addition to summary statistics, the data points behind means, medians and variance measures should be available. If there are restrictions on publicly sharing data—e.g. participant privacy or use of data from a third party—those must be specified.

Reviewer #3: Yes

Reviewer #4: Yes

**********

5. Is the manuscript presented in an intelligible fashion and written in standard English?

PLOS Global Public Health does not copyedit accepted manuscripts, so the language in submitted articles must be clear, correct, and unambiguous. Any typographical or grammatical errors should be corrected at revision, so please note any specific errors here.

Reviewer #3: Yes

Reviewer #4: Yes

**********

6. Review Comments to the Author

Please use the space provided to explain your answers to the questions above. You may also include additional comments for the author, including concerns about dual publication, research ethics, or publication ethics. (Please upload your review as an attachment if it exceeds 20,000 characters)

Reviewer #3: This study has evaluated the causal relationships between the Human Footprint Index and the excess cases of three vector-borne diseases in Colombia: dengue, malaria, and visceral leishmaniasis. Dengue showed the highest positive association, followed by malaria, while no significant association was observed for visceral leishmaniasis.

The combination of DAGs with machine learning makes this a methodologically robust study. This approach also has the potential to be replicated for other environmentally-sensitive infectious diseases and regions, taking into account the local epidemiological context. The authors have clearly addressed the main comments from a previous round of reviews (which I was not part of), and the manuscript is now clearer and close to publication quality.

That said, I missed a deeper explanation of the local and regional eco-epidemiology of visceral leishmaniasis (VL). While the manuscript provides species-level detail for malaria vectors and parasites (Anopheles and Plasmodium), the same level of taxonomic and ecological detail is missing for Leishmania spp. and their sandfly vectors (Lutzomyia spp.). Colombia has a wide diversity of Lutzomyia and Leishmania species, each with distinct ecological preferences: some more adapted to rural environments, others to peri-urban or domestic habitats. VL has been closely linked to urbanization throughout Latin America, primarily due to the peri-urban distribution and specific habitat and feeding preferences of its principal vector, Lutzomyia longipalpis.

Furthermore, the methods section should clarify how leishmaniasis data from SIVIGILA were processed. Unlike the clear description provided for malaria (e.g., aggregation across Plasmodium species), it is not specified whether SIVIGILA distinguishes between cutaneous and visceral leishmaniasis, and whether only confirmedd VL cases were analyzed. If both forms were included by chance, even if that is a limitation of the main data source, there is a risk that the causal signals are diluted, thus reducing the statistical significance of the results.

Including these methodological details and improving the discussion of how case reporting and difference between VL and CL may influence the relationship with HFP would add depth to the study and improve the interpretability of the results.

Reviewer #4: Anthropogenic Landscapes and Vector-Borne Disease Dynamics: Unveiling the Complex Interplay between Human Footprint and Disease Transmission in Colombia

Summary: I really enjoyed reading this manuscript and found the use of a double machine learning approach for causal inference exciting. I have two major critiques:

1. Methods: The methods are not explained thoroughly enough for readers who are unfamiliar with them. I noted several places where the explanation could be elaborated. Since PLoS is not restrictive with word count, adding more detail should not be an issue.

2. Methods: VL cases are often very zero inflated. How is this accounted for in the modeling framework?

3. Ecology of visceral leishmaniasis: The ecology of visceral leishmaniasis is inaccurately described throughout the study, which affects the choice of confounders and the interpretation of results. VL is transmitted primarily by a single sandfly species, uses dogs as reservoir hosts, and is caused by a single Leishmania species. It is typically not associated with forest environments.

Intro:

• Pg 3, p1: Are there certain key malaria vectors that are more important to the ecology discussed in the manuscript than others?

• Pg 3, p1: Not all sandfly vectors are nocturnal, and not all are adapted to peridomestic environments (see comment below).

• Pg 3, p1: Visceral leishmaniasis is caused by one specific Leishmania species (Leishmania infantum) and has primarily one sandfly vector (Lu. longipalpis). The ecology of VL is very different from that of CL and MCL, so they should not be grouped together when discussing the ecology and transmission cycles of the diseases.

• Overall:

I think it is important to include more background on the three diseases as it applies to their specific relationships with different landscape types and human pressures, making sure to adequately cite background literature. For instance, there is a lot of literature discussing the nuances of malaria and deforestation that might be critical for understanding the results and for better contextualizing the manuscript’s novel contribution to our understanding of malaria.

Methods:

• Table 1: I really like Table 1; it does a great job of describing the variables used.

• Page 6, p3: I don’t think visceral leishmaniasis is typically classified as “urban and rural”; it is usually described as “urban and peri-urban.” Please make sure you are not confusing the VL transmission cycle with those of CL and MCL.

• Pg 5, Causal inference and machine learning technique: Did you test variables for multicollinearity? Even though gradient boosting models (GBMs) can handle higher multicollinearity than classical statistical models, it can still be problematic. Strong correlations (ρ > 0.7) can increase variance, reduce interpretability, and contribute to overfitting if redundant predictors are included.

• Pg 6, p3: How is U estimated? Is it based on spatio-temporal autocorrelation? Is it more like a random intercept matrix?

• Pg 6, p4: Did you train a model for each fold, or for three combinations of two folds (with the third as the hold-out)? Was this step used to test out-of-sample predictive performance, or to identify variation in model outcomes depending on the partition of data? Why did you choose a learning rate of 0.01 and not tune this hyperparameter? Please clarify in the text.

• Pg 6, p4: Did you use spatio-temporal cross-validation? If so, please explain. If not, please justify why spatio-temporal cross-validation does not need to be used for this methodology, given the expectation of spatial and temporal autocorrelation.

• Pg 7, Robustness Analysis: Please explain what each of these tests are. Additionally, clarify what you mean by “latent bias in the estimate.”

• Pg 8, p1: Why was HFP binarized? What do you mean by this? For readers not familiar with the analysis, it sounds as though the variable was transformed into 0 vs 1, which would lose a lot of information and defeat the purpose of using HFP as a variable.

Results

• Pg 8, p3: How do the ATE estimates translate into excess disease cases?

• Pg 8, p3: VL transmission is typically stochastic and sparse. Could this influence the model’s ability to detect an HFP signal? How is zero-inflation/overdispersion accounted for in the model?

• Pg 8, p2–3: I think these qualitative results could serve as the opening of the Results section to better contextualize the system. Please place the maps of HFP and case distributions in the main text so they are easier to reference.

• Pg 8–9: The ATE estimates for dengue have very wide confidence intervals. Is this expected for this type of analysis? What are the implications of such wide CIs for interpreting the results?

• Pg 8–9 (bottom and top paragraphs): I am not sure what the numbers in parentheses after “municipalities with HFP larger than the median” refer to.

• Pg 9, p2: I really like the CATE analysis. Have you examined it for climate? Perhaps dengue is more sensitive to HFP in areas at the edge of climate suitability, where HFP might help create small pockets of more favorable transmission conditions in regions that would otherwise be too cool or dry.

• Pg 9, p3: I am not sure what this means. Please elaborate on how this was estimated and why it is important.

• Overall: Please redefine ATE and CATE in the Discussion so the reader doesn’t need to search back through the paper to recall their definitions.

Discussion:

• Pg 10: “In fact, in a global perspective, countries that were not considered endemic for certain diseases a few years ago now report infections caused by vectors locally”: please provide a citation.

• Pg 10, p3: If you do not statistically investigate how climate might influence these relationships and the resulting lack of statistical significance, it would be interesting to include a discussion on how incorporating a climate interaction could mediate the HFP × misery interaction.

• Pg 10, p4 – Pg 11, p1: This is a lot of discussion for a result that was not significant. Consider moving this paragraph above the prior paragraph (Pg 10, p3) to first set the reader up with why misery should be a significant interactor, then present your result, and finally explain why the relationship was not significant.

• Pg 11, p3: This information is not relevant for visceral leishmaniasis, which is primarily caused by a single species, transmitted by one or two vector species, and typically maintained by domestic or built-environment–adapted reservoir hosts.

• Pg 12, p3: While VL occurs in rural and peri-urban fringes, both the sandflies and reservoir hosts are not forest-associated. Deforestation and logging should not directly influence VL; instead, they may influence transmission indirectly, on a time lag, as cleared land is converted to other land uses more closely related to VL ecology.

• Pg 13, p3: The Skinner paper also investigates each of these diseases individually. Therefore, the sentence “our research narrows its focus concentrating on dengue, malaria and visceral leishmaniasis” does not make sense in relation to how it builds on Skinner. The exciting addition of this manuscript is that it uses a causal inference approach and includes socio-economic factors.

NOTE: The manuscript did not include line numbers. Please include them in the future to make it easier to give feedback.

**********

7. PLOS authors have the option to publish the peer review history of their article (what does this mean?). If published, this will include your full peer review and any attached files.

**Do you want your identity to be public for this peer review?**  If you choose “no”, your identity will remain anonymous but your review may still be made public.

For information about this choice, including consent withdrawal, please see our Privacy Policy.

Reviewer #3: No

Reviewer #4: No

**********

[NOTE: If reviewer comments were submitted as an attachment file, they will be attached to this email and accessible via the submission site. Please log into your account, locate the manuscript record, and check for the action link "View Attachments". If this link does not appear, there are no attachment files.]

While revising your submission, please upload your figure files to the Preflight Analysis and Conversion Engine (PACE) digital diagnostic tool, https://pacev2.apexcovantage.com/. PACE helps ensure that figures meet PLOS requirements. To use PACE, you must first register as a user. Registration is free. Then, login and navigate to the UPLOAD tab, where you will find detailed instructions on how to use the tool. If you encounter any issues or have any questions when using PACE, please email PLOS at figures@plos.org. Please note that Supporting Information files do not need this step.

Attachment Submitted filename: Anthropogenic Landscapes and Vector.docx

# Author comment: Author response to Decision Letter 2

**DOI:** 10.1371/journal.pgph.0006801.r004

3 Nov 2025

Attachment Submitted filename: Response_reviewers_110325.pdf

# Peer review: Decision Letter 2

**DOI:** 10.1371/journal.pgph.0006801.r005

**Authors:** Jennifer Tucker

22 Dec 2025

PGPH-D-24-02579R2

Anthropogenic Landscapes and Vector-Borne Disease Dynamics: Unveiling the Complex Interplay between Human Footprint and Disease Transmission in Colombia

PLOS Global Public Health

Dear Dr. Cuadros,

Thank you for submitting your manuscript to PLOS Global Public Health. After careful consideration, we feel that it has merit but does not fully meet PLOS Global Public Health’s publication criteria as it currently stands. Therefore, we invite you to submit a revised version of the manuscript that addresses the points raised during the review process.

Review has found additional revisions which are required before your manuscript can be considered for publication, particularly in relation to the methodology and some study limitations which have not been fully addressed in the current article.

Please submit your revised manuscript by Feb 02 2026 11:59PM. If you will need more time than this to complete your revisions, please reply to this message or contact the journal office at globalpubhealth@plos.org. When you're ready to submit your revision, log on to https://www.editorialmanager.com/pgph/ and select the 'Submissions Needing Revision' folder to locate your manuscript file.

Please include the following items when submitting your revised manuscript:

- A letter that responds to each point raised by the editor and reviewer(s). You should upload this letter as a separate file labeled 'Response to Reviewers'.
- A marked-up copy of your manuscript that highlights changes made to the original version. You should upload this as a separate file labeled 'Revised Manuscript with Track Changes'.
- An unmarked version of your revised paper without tracked changes. You should upload this as a separate file labeled 'Manuscript'.

Guidelines for resubmitting your figure files are available below the reviewer comments at the end of this letter.

We look forward to receiving your revised manuscript.

Kind regards,

Jennifer Tucker, PhD

Staff Editor

PLOS Global Public Health

Journal Requirements:

1. Please note that PLOS Global Public Health has specific guidelines on code sharing for submissions in which author-generated code underpins the findings in the manuscript. In these cases, all author-generated code must be made available without restrictions upon publication of the work. Please review our guidelines at https://journals.plos.org/globalpublichealth/s/materials-and-software-sharing#loc-sharing-code and ensure that your code is shared in a way that follows best practice and facilitates reproducibility and reuse.

If the reviewer comments include a recommendation to cite specific previously published works, please review and evaluate these publications to determine whether they are relevant and should be cited. There is no requirement to cite these works unless the editor has indicated otherwise.

Additional Editor Comments (if provided):

[Note: HTML markup is below. Please do not edit.]

Reviewers' comments:

Reviewer's Responses to Questions

**Comments to the Author**

1. If the authors have adequately addressed your comments raised in a previous round of review and you feel that this manuscript is now acceptable for publication, you may indicate that here to bypass the “Comments to the Author” section, enter your conflict of interest statement in the “Confidential to Editor” section, and submit your "Accept" recommendation.

Reviewer #5: All comments have been addressed

**********

2. Does this manuscript meet PLOS Global Public Health’s publication criteria? Is the manuscript technically sound, and do the data support the conclusions? The manuscript must describe methodologically and ethically rigorous research with conclusions that are appropriately drawn based on the data presented.

Reviewer #5: Partly

**********

3. Has the statistical analysis been performed appropriately and rigorously?

Reviewer #5: No

**********

4. Have the authors made all data underlying the findings in their manuscript fully available (please refer to the Data Availability Statement at the start of the manuscript PDF file)?

The PLOS Data policy requires authors to make all data underlying the findings described in their manuscript fully available without restriction, with rare exception. The data should be provided as part of the manuscript or its supporting information, or deposited to a public repository. For example, in addition to summary statistics, the data points behind means, medians and variance measures should be available. If there are restrictions on publicly sharing data—e.g. participant privacy or use of data from a third party—those must be specified.

Reviewer #5: No

**********

5. Is the manuscript presented in an intelligible fashion and written in standard English?

PLOS Global Public Health does not copyedit accepted manuscripts, so the language in submitted articles must be clear, correct, and unambiguous. Any typographical or grammatical errors should be corrected at revision, so please note any specific errors here.

Reviewer #5: No

**********

6. Review Comments to the Author

Please use the space provided to explain your answers to the questions above. You may also include additional comments for the author, including concerns about dual publication, research ethics, or publication ethics. (Please upload your review as an attachment if it exceeds 20,000 characters)

Reviewer #5: The manuscript presents an ambitious and timely attempt to explore the relationship between the Human Footprint Index and the risk of dengue, malaria, and visceral leishmaniasis across municipalities in Colombia using modern causal inference approaches such as Double Machine Learning (DML) and EconML. The topic is undeniably important and has the potential to advance understanding of how human activities and environmental transformations influence vector-borne disease dynamics. However, despite its relevance and the innovative framework employed, the manuscript still suffers from substantial conceptual, methodological, and interpretational weaknesses that must be addressed before it can be considered for publication.

One of the most critical concerns lies in the presence of residual bias detected by the refutation tests. The authors themselves report that replacing a random 10% subset of data produced significantly different estimates of the average treatment effect, indicating that the models still contain unaccounted bias. This directly undermines the causal interpretation of the findings. The refutation results should be clearly reported in the main text and discussed in detail, rather than being briefly summarized in a table. The authors should conduct further robustness analyses, including alternative thresholds for defining high versus low human footprint, spatial and temporal block cross-validation to account for dependency, and sensitivity analyses such as E-values or Rosenbaum bounds to assess the influence of unmeasured confounding. Without these additional steps, the study’s causal conclusions remain tentative.

Another major issue is the lack of explicit control for spatial and temporal autocorrelation. Vector-borne disease data are inherently spatially and temporally correlated, and failing to model this structure can lead to underestimated uncertainty and inflated statistical significance. The authors acknowledge this limitation but provide no quantitative diagnostics or model corrections. The use of municipality and year fixed effects, or alternatively a spatio-temporal blocking approach within the cross-validation framework, would substantially improve the robustness of the causal estimates. Including diagnostics such as Moran’s I or semivariograms of residuals would also demonstrate whether spatial dependence has been adequately accounted for.

The outcome definition also requires reconsideration. The binary variable of “excess cases” (standardized incidence ratio > 1) oversimplifies the data and changes the interpretation from incidence rate to the probability of surpassing an arbitrary threshold. This transformation may obscure important variations in disease risk and reduce statistical power. The authors should perform sensitivity analyses using continuous or count-based outcomes such as incidence rates, log-transformed standardized incidence ratios, or zero-inflated count models to verify that their results are not an artifact of dichotomization. Such analyses would help clarify the magnitude and meaning of the reported causal effects.

Temporal alignment between exposure, covariates, and outcome is another key weakness. The Human Footprint dataset extends only to 2018, while the disease data run through 2019, and the manuscript does not explain how this discrepancy was addressed. It is also unclear whether the exposure in year t was used to predict disease in the same year or the following year. Without an explicit lag structure, there is a risk of reverse causation, where disease occurrence might influence local human activities or data recording. The authors should clarify their temporal design and perform sensitivity analyses using lagged exposures to test for robustness. Properly aligning the data would make the causal claims much more defensible.

The nuisance models used in the DML framework are also under-specified. The authors report using XGBoost with fixed parameters but admit that no systematic hyperparameter tuning was performed. Given that DML heavily relies on the quality of the nuisance models for valid causal estimation, lack of tuning can seriously affect bias correction and variance reduction. The authors should conduct cross-validated hyperparameter optimization, report model diagnostics such as feature importance or partial dependence plots, and provide reproducible details about the learning rate, tree depth, and regularization parameters. These details are essential for transparency and reproducibility.

The selection and justification of confounders also need improvement. The paper refers to directed acyclic graphs (DAGs) and an unmeasured confounder U but does not show the DAGs or provide a full explanation of the role of each variable in the model. The inclusion of static covariates such as urbanization measures from 2015 and 2018 without accounting for temporal variability may introduce measurement bias. The authors should include their DAGs in the supplementary materials and clearly categorize each covariate as a confounder, mediator, or collider. Additionally, testing models with and without potential mediators (e.g., deforestation or land-use variables) would reveal how much these variables influence the estimated treatment effects.

The component of the analysis related to visceral leishmaniasis is particularly fragile due to the extremely low number of cases. The small sample size yields wide confidence intervals and unstable conditional treatment effects. The authors acknowledge this limitation but still draw relatively strong interpretations from these results. Given the low statistical power, the visceral leishmaniasis results should be described as exploratory and interpreted with caution rather than as evidence of robust causal relationships.

Moreover, potential temporal bias arising from surveillance system changes has not been adequately addressed. The SIVIGILA surveillance system underwent methodological changes during the study period, and such modifications can distort temporal disease trends. The authors should include year fixed effects or perform analyses stratified by pre- and post-surveillance-change periods to confirm that the main findings are not artifacts of data collection inconsistencies.

While the authors’ approach is methodologically sophisticated, the presentation of results lacks clarity and consistency. Refutation tests, conditional treatment effects, and uncertainty intervals are sometimes reported without sufficient explanation. The figures should include density indications or histograms to illustrate where the data are concentrated, preventing overinterpretation of tails with low data density. The causal interpretations should be tempered throughout the manuscript, replacing overly deterministic language such as “HFP causes an increase in dengue” with more accurate phrasing like “HFP is associated with a higher probability of excess dengue incidence under the study’s assumptions.” This more cautious language would better reflect the statistical and causal limitations acknowledged by the authors themselves.

Despite these limitations, the study has significant potential. It integrates multiple datasets, applies advanced causal inference tools, and addresses an important topic for public health and environmental epidemiology. With additional methodological rigor, better documentation of analytical choices, and refined interpretation of results, the paper could become a valuable contribution to the literature on the environmental determinants of vector-borne diseases.

The authors are also encouraged to cite the following recent and highly relevant studies to strengthen the background, discussion, and contextualization of their findings, as these works are closely aligned with their research objectives and methodologies. Abbasi (2025) “The impact of climate change on Aedes aegypti distribution and dengue fever prevalence in semi-arid regions” in Environmental Research provides direct empirical evidence linking climatic shifts and vector ecology, which complements the current study’s focus on dengue risk. Abbasi (2025) “The impact of climate change on travel-related vector-borne diseases” in Travel Medicine and Infectious Disease expands the discussion to human mobility and global disease spread, reinforcing the human footprint theme. Abbasi (2025) “Global expansion of Aedes mosquitoes and their role in the transboundary spread of emerging arboviral diseases” in IJID One Health offers global-scale confirmation of urbanization-driven mosquito proliferation, supporting the Colombian findings. Abbasi (2025) “Emerging and transboundary arboviral diseases: the role of insect vectors and key drivers such as climate change and urbanization” in International Journal of Tropical Insect Science should also be cited to connect the results to global patterns of arboviral emergence. Abbasi (2025) “Climate change and vector-borne disease transmission: the role of insect behavioral and physiological adaptations” in Integrative Organismal Biology provides mechanistic insight into how vectors adapt to anthropogenic and climatic pressures, strengthening the biological interpretation of the observed trends. Abbasi (2025) “Global Trends in the Burden of Malaria” aligns with the malaria component of the study, while Abbasi (2025) “Advancing Insights into Visceral Leishmaniasis” provides an up-to-date overview of leishmaniasis dynamics and management, enhancing the discussion of that disease. Additionally, Abbasi (2025) “Application of remote sensing and geospatial technologies in predicting vector-borne disease outbreaks” in Royal Society Open Science is methodologically relevant, as it parallels the current manuscript’s use of spatial modeling and remote-sensing data. Finally, Abbasi (2025) “Ecotoxicological implications of environmental contaminants on disease vectors” in Environmental Toxicology and Chemistry and Abbasi (2025) “Insecticide Resistance and Arboviral Disease Transmission” in The Journal of Advances in Parasitology together strengthen the conceptual framework connecting human environmental impact, chemical exposure, and vector adaptation. Incorporating these references would not only update the literature review but also situate the study within the rapidly evolving global discourse on climate, human activity, and vector-borne disease epidemiology.

**********

7. PLOS authors have the option to publish the peer review history of their article (what does this mean?). If published, this will include your full peer review and any attached files.

**Do you want your identity to be public for this peer review?**  If you choose “no”, your identity will remain anonymous but your review may still be made public.

For information about this choice, including consent withdrawal, please see our Privacy Policy.

Reviewer #5: **Yes:**  Ebrahim Abbasi

**********

[NOTE: If reviewer comments were submitted as an attachment file, they will be attached to this email and accessible via the submission site. Please log into your account, locate the manuscript record, and check for the action link "View Attachments". If this link does not appear, there are no attachment files.]

-->Figure Resubmissions:

While revising your submission, we strongly recommend that you use PLOS’s NAAS tool (https://ngplosjournals.pagemajik.ai/artanalysis) to test your figure files. NAAS can convert your figure files to the TIFF file type and meet basic requirements (such as print size, resolution), or provide you with a report on issues that do not meet our requirements and that NAAS cannot fix.

After uploading your figures to PLOS’s NAAS tool - https://ngplosjournals.pagemajik.ai/artanalysis, NAAS will process the files provided and display the results in the "Uploaded Files" section of the page as the processing is complete. If the uploaded figures meet our requirements (or NAAS is able to fix the files to meet our requirements), the figure will be marked as "fixed" above. If NAAS is unable to fix the files, a red "failed" label will appear above. When NAAS has confirmed that the figure files meet our requirements, please download the file via the download option, and include these NAAS processed figure files when submitting your revised manuscript.

# Author comment: Author response to Decision Letter 3

**DOI:** 10.1371/journal.pgph.0006801.r006

29 Jan 2026

Attachment Submitted filename: Response_reviewers_110325_auresp_3.pdf

# Peer review: Decision Letter 3

**DOI:** 10.1371/journal.pgph.0006801.r007

**Authors:** Jennifer Tucker

12 Mar 2026

PGPH-D-24-02579R3

Anthropogenic Landscapes and Vector-Borne Disease Dynamics: Unveiling the Complex Interplay between Human Footprint and Disease Transmission in Colombia

PLOS Global Public Health

Dear Dr. Cuadros,

Thank you for submitting your manuscript to PLOS Global Public Health. After careful consideration, we feel that it has merit but does not fully meet PLOS Global Public Health’s publication criteria as it currently stands. Therefore, we invite you to submit a revised version of the manuscript that addresses the points raised during the review process.

Reviewers 2 and 6 request some final minor revisions to provide clarity regarding aspects of the models design and validation, and completeness in discussion of the study limitations. Please disregard reviewer 6's comments, as the citation requests are not aligned with expected reviewer conduct.

I look forward to seeing the revisions complete and proceeding with supporting your manuscript to publication.

Please submit your revised manuscript by Apr 10 2026 11:59PM. If you will need more time than this to complete your revisions, please reply to this message or contact the journal office at globalpubhealth@plos.org. When you're ready to submit your revision, log on to https://www.editorialmanager.com/pgph/ and select the 'Submissions Needing Revision' folder to locate your manuscript file.

Please include the following items when submitting your revised manuscript:

- A letter that responds to each point raised by the editor and reviewer(s). You should upload this letter as a separate file labeled 'Response to Reviewers'.
- A marked-up copy of your manuscript that highlights changes made to the original version. You should upload this as a separate file labeled 'Revised Manuscript with Track Changes'.
- An unmarked version of your revised paper without tracked changes. You should upload this as a separate file labeled 'Manuscript'.

Guidelines for resubmitting your figure files are available below the reviewer comments at the end of this letter.

We look forward to receiving your revised manuscript.

Kind regards,

Jennifer Tucker, PhD

Staff Editor

PLOS Global Public Health

Journal Requirements:

If the reviewer comments include a recommendation to cite specific previously published works, please review and evaluate these publications to determine whether they are relevant and should be cited. There is no requirement to cite these works unless the editor has indicated otherwise.

Please review your reference list to ensure that it is complete and correct. If you have cited papers that have been retracted, please include the rationale for doing so in the manuscript text, or remove these references and replace them with relevant current references. Any changes to the reference list should be mentioned in the rebuttal letter that accompanies your revised manuscript. If you need to cite a retracted article, indicate the article’s retracted status in the References list and also include a citation and full reference for the retraction notice.

Additional Editor Comments (if provided):

[Note: HTML markup is below. Please do not edit.]

Reviewers' comments:

Reviewer's Responses to Questions

**Comments to the Author**

1. If the authors have adequately addressed your comments raised in a previous round of review and you feel that this manuscript is now acceptable for publication, you may indicate that here to bypass the “Comments to the Author” section, enter your conflict of interest statement in the “Confidential to Editor” section, and submit your "Accept" recommendation.

Reviewer #2: All comments have been addressed

Reviewer #4: All comments have been addressed

Reviewer #5: All comments have been addressed

Reviewer #6: (No Response)

**********

2. Does this manuscript meet PLOS Global Public Health’s publication criteria? Is the manuscript technically sound, and do the data support the conclusions? The manuscript must describe methodologically and ethically rigorous research with conclusions that are appropriately drawn based on the data presented.

Reviewer #2: (No Response)

Reviewer #4: Yes

Reviewer #5: Partly

Reviewer #6: Partly

**********

3. Has the statistical analysis been performed appropriately and rigorously?

Reviewer #2: Yes

Reviewer #4: Yes

Reviewer #5: No

Reviewer #6: Yes

**********

4. Have the authors made all data underlying the findings in their manuscript fully available (please refer to the Data Availability Statement at the start of the manuscript PDF file)?

The PLOS Data policy requires authors to make all data underlying the findings described in their manuscript fully available without restriction, with rare exception. The data should be provided as part of the manuscript or its supporting information, or deposited to a public repository. For example, in addition to summary statistics, the data points behind means, medians and variance measures should be available. If there are restrictions on publicly sharing data—e.g. participant privacy or use of data from a third party—those must be specified.

Reviewer #2: Yes

Reviewer #4: Yes

Reviewer #5: No

Reviewer #6: Yes

**********

5. Is the manuscript presented in an intelligible fashion and written in standard English?

PLOS Global Public Health does not copyedit accepted manuscripts, so the language in submitted articles must be clear, correct, and unambiguous. Any typographical or grammatical errors should be corrected at revision, so please note any specific errors here.

Reviewer #2: Yes

Reviewer #4: Yes

Reviewer #5: No

Reviewer #6: Yes

**********

6. Review Comments to the Author

Please use the space provided to explain your answers to the questions above. You may also include additional comments for the author, including concerns about dual publication, research ethics, or publication ethics. (Please upload your review as an attachment if it exceeds 20,000 characters)

Reviewer #2: I think nearly all comments were very thoroughly and adequately addressed, there is only one thing that could be clarified a bit more prior to publication- specifically, whether the size and direction of the treatment effect stay basically the same, and whether turning HFP into a binary variable changes interpretation or understanding of the results.

Reviewer #4: The authors carefully addressed all comments. I am excited about the methodological innovation and scientific contribution this manuscript will add to the field of VBD ecology and public health.

Reviewer #5: With respect, the study was statistically and content-reviewed. The authors recommend that the following corrections be made to ensure success.

The manuscript presents an ambitious attempt to quantify the relationship between human environmental pressure and the risk of multiple vector-borne diseases using a causal machine-learning framework. However, the study repeatedly overstates its capacity for causal inference. Despite frequent references to “causal effects,” “causal links,” and “causal machine learning,” the underlying design remains ecological and observational, relying on aggregated municipal-level data. The exposure and outcome variables are measured at the same temporal resolution, with limited attention to temporal ordering or lag structures that would be necessary to support causal interpretations. Key identification assumptions are asserted rather than justified, and the language used throughout the manuscript gives the impression of causal certainty that is not warranted by the data or study design.

A major conceptual weakness concerns the treatment of the human footprint index as a single, unified exposure. Human footprint is a composite indicator that aggregates heterogeneous dimensions of anthropogenic activity, including urbanization, infrastructure, population density, and land-use change. These components are likely to influence different vectors and diseases through distinct, and sometimes opposing, mechanisms. By collapsing them into a single treatment variable, the analysis obscures biological and ecological pathways and limits interpretability. This limitation becomes particularly evident when the same index is used to explain increased dengue risk, decreased malaria risk, and weak or unstable effects for visceral leishmaniasis, largely through post hoc ecological narratives rather than explicitly tested hypotheses.

The ecological nature of the data is not sufficiently respected in the interpretation. Colombian municipalities vary widely in size, internal heterogeneity, and ecological composition, yet they are treated as homogeneous analytical units. The discussion frequently invokes household- or individual-level mechanisms, such as housing conditions, domestic animal reservoirs, or human–vector contact, without corresponding data at those levels. This creates a substantial risk of ecological fallacy, as associations observed at the municipal scale are implicitly translated into explanations operating at finer scales that cannot be supported by the analysis.

The operationalization of disease outcomes as binary indicators of “excess cases” based on standardized incidence ratios greater than one is another concern. This approach discards important information on disease burden and outbreak intensity, treating municipalities with marginally elevated incidence the same as those experiencing severe transmission. The binary transformation introduces sensitivity to arbitrary thresholds, reduces statistical power, and complicates interpretation of the estimated effects, which are expressed as changes in the probability of exceeding a threshold rather than changes in disease burden.

Although the dataset spans more than a decade, the temporal dimension is underexploited. The analysis does not incorporate explicit modeling of temporal trends, lagged environmental effects, or year-specific shocks, nor does it include time fixed effects to account for changes in surveillance systems, intervention strategies, or national-level climatic anomalies. Several covariates, particularly socio-economic indicators, are drawn from a single census year and implicitly assumed to be constant over the entire study period, creating temporal misalignment between exposure, confounders, and outcomes that undermines causal interpretation.

The causal framework and modeling strategy raise additional concerns. Directed acyclic graphs are referenced but not fully articulated or defended, and the acknowledgment of unmeasured confounding remains largely symbolic. While double machine learning is an appropriate tool for handling high-dimensional covariates and nonlinear relationships, it cannot compensate for unmeasured confounding, measurement error, or weak identification. The manuscript risks presenting methodological sophistication as a substitute for strong causal assumptions, which may mislead readers about the robustness of the findings.

Statistical choices are not always sufficiently justified. Hyperparameter settings for the machine-learning models appear arbitrary, with no tuning strategy or sensitivity analysis reported. Spatial and temporal autocorrelation—well-known features of infectious disease and environmental data are not explicitly modeled, raising concerns about biased standard errors and overconfident inference. The conditional average treatment effect analyses, while visually appealing, rely on holding other modifiers at their mean values, which may lack ecological meaning, and trends are interpreted despite wide confidence intervals in key regions of the covariate space.

Finally, the refutation analyses reveal internal inconsistencies. At least one refutation test indicates remaining bias, yet this finding does not meaningfully temper the manuscript’s conclusions. The continued emphasis on robustness and causal interpretation sits uneasily with evidence suggesting sensitivity to data perturbation. Policy recommendations regarding urban planning, surveillance prioritization, and resource allocation extend beyond what can be supported by the presented evidence and should be substantially softened.

With respect to citations, only a limited number of the listed articles are genuinely consistent with the content, scale, and conceptual framing of the study and should be cited.

The authors should cite “The impact of climate change on travel-related vector-borne diseases: a case study on dengue virus transmission” (Abbasi, 2025, Travel Medicine and Infectious Disease). This article is consistent with the manuscript’s focus on large-scale environmental and climatic drivers of dengue transmission and provides relevant contextual background on how environmental change influences dengue risk. The citation would help situate the study within the broader literature on climate-sensitive arboviral diseases without implying methodological equivalence.

They should also cite “Global expansion of Aedes mosquitoes and their role in the transboundary spread of emerging arboviral diseases: a comprehensive review” (Abbasi, 2025, IJID One Health). This review directly supports the manuscript’s interpretation of dengue results by documenting how urbanization, human mobility, and anthropogenic landscape change facilitate the expansion of Aedes vectors. It strengthens the biological plausibility of the observed association between human footprint and dengue excess risk.

Another appropriate citation is “The impact of climate change on Aedes aegypti distribution and dengue fever prevalence in semi-arid regions” (Abbasi, 2025, Environmental Research). This study aligns with the manuscript’s ecological perspective by linking environmental gradients and climate variability to dengue prevalence. Citing it would reinforce the argument that macro-environmental conditions are important determinants of dengue transmission, while clearly framing it as contextual evidence rather than causal validation.

Finally, the authors may cite “Emerging and transboundary arboviral diseases: the role of insect vectors and key drivers such as climate change and urbanization” (Abbasi, 2025, International Journal of Tropical Insect Science). This article provides a conceptual synthesis of how urbanization and environmental change drive arboviral emergence, which is consistent with the manuscript’s use of the human footprint index as a proxy for anthropogenic pressure.

In contrast, the numerous articles focused on insecticide resistance mechanisms, molecular surveillance, head lice, ticks, edible insects, medical education, forensic entomology, or unrelated technological and pedagogical topics are not consistent with the manuscript’s objectives or analytical scale and should not be cited. Selective citation of conceptually aligned, macro-scale environmental and vector-borne disease literature will strengthen the manuscript, whereas broad citation of tangentially related work would dilute its focus and credibility.

Reviewer #6: This study uses machine learning models to investigate the effect of the human footprint (HFP) on the transmission of vector-borne diseases (VBDs) in Colombia. They start proposing causal models (DAGs) for the relationship between HFP and excess VBD, considering climate and sociodemograíc variables as confounders. Overall, the study is relevant and innovative as it applies modern machine learning approaches to estimate causal effects. My comments are mainly related to improve the clarity of the model and the scope of the discussion: see the uploaded file

**********

7. PLOS authors have the option to publish the peer review history of their article (what does this mean?). If published, this will include your full peer review and any attached files.

**Do you want your identity to be public for this peer review?**  If you choose “no”, your identity will remain anonymous but your review may still be made public.

For information about this choice, including consent withdrawal, please see our Privacy Policy.

Reviewer #2: No

Reviewer #4: No

Reviewer #5: **Yes:**  Ebrahim Abbasi

Reviewer #6: No

**********

[NOTE: If reviewer comments were submitted as an attachment file, they will be attached to this email and accessible via the submission site. Please log into your account, locate the manuscript record, and check for the action link "View Attachments". If this link does not appear, there are no attachment files.]

Figure Resubmissions:

While revising your submission, we strongly recommend that you use PLOS’s NAAS tool (https://ngplosjournals.pagemajik.ai/artanalysis) to test your figure files. NAAS can convert your figure files to the TIFF file type and meet basic requirements (such as print size, resolution), or provide you with a report on issues that do not meet our requirements and that NAAS cannot fix.

After uploading your figures to PLOS’s NAAS tool - https://ngplosjournals.pagemajik.ai/artanalysis, NAAS will process the files provided and display the results in the "Uploaded Files" section of the page as the processing is complete. If the uploaded figures meet our requirements (or NAAS is able to fix the files to meet our requirements), the figure will be marked as "fixed" above. If NAAS is unable to fix the files, a red "failed" label will appear above. When NAAS has confirmed that the figure files meet our requirements, please download the file via the download option, and include these NAAS processed figure files when submitting your revised manuscript.

Attachment Submitted filename: PLos.odt

# Author comment: Author response to Decision Letter 4

**DOI:** 10.1371/journal.pgph.0006801.r008

30 Apr 2026

Attachment Submitted filename: Response to Reviewers.pdf

# Peer review: Decision Letter 4

**DOI:** 10.1371/journal.pgph.0006801.r009

**Authors:** Julia Robinson

17 Jun 2026

Anthropogenic Landscapes and Vector-Borne Disease Dynamics: Unveiling the Complex Interplay between Human Footprint and Disease Transmission in Colombia

PGPH-D-24-02579R4

Dear Dr. Cuadros,

We are pleased to inform you that your manuscript 'Anthropogenic Landscapes and Vector-Borne Disease Dynamics: Unveiling the Complex Interplay between Human Footprint and Disease Transmission in Colombia' has been provisionally accepted for publication in PLOS Global Public Health.

Before your manuscript can be formally accepted you will need to complete some formatting changes, which you will receive in a follow up email. A member of our team will be in touch with a set of requests.

Please note that your manuscript will not be scheduled for publication until you have made the required changes, so a swift response is appreciated.

IMPORTANT: The editorial review process is now complete. PLOS will only permit corrections to spelling, formatting or significant scientific errors from this point onwards. Requests for major changes, or any which affect the scientific understanding of your work, will cause delays to the publication date of your manuscript.

If your institution or institutions have a press office, please notify them about your upcoming paper to help maximize its impact. If they'll be preparing press materials, please inform our press team as soon as possible -- no later than 48 hours after receiving the formal acceptance. Your manuscript will remain under strict press embargo until 2 pm Eastern Time on the date of publication. For more information, please contact globalpubhealth@plos.org.

Thank you again for supporting Open Access publishing; we are looking forward to publishing your work in PLOS Global Public Health.

Best regards,

Julia Robinson

Executive Editor

PLOS Global Public Health

***********************************************************

Reviewer Comments (if any, and for reference):

Reviewer's Responses to Questions

**Comments to the Author**

1. If the authors have adequately addressed your comments raised in a previous round of review and you feel that this manuscript is now acceptable for publication, you may indicate that here to bypass the “Comments to the Author” section, enter your conflict of interest statement in the “Confidential to Editor” section, and submit your "Accept" recommendation.

Reviewer #2: All comments have been addressed

**********

2. Does this manuscript meet PLOS Global Public Health’s publication criteria? Is the manuscript technically sound, and do the data support the conclusions? The manuscript must describe methodologically and ethically rigorous research with conclusions that are appropriately drawn based on the data presented.

Reviewer #2: Yes

**********

3. Has the statistical analysis been performed appropriately and rigorously?

Reviewer #2: Yes

**********

4. Have the authors made all data underlying the findings in their manuscript fully available (please refer to the Data Availability Statement at the start of the manuscript PDF file)?

The PLOS Data policy requires authors to make all data underlying the findings described in their manuscript fully available without restriction, with rare exception. The data should be provided as part of the manuscript or its supporting information, or deposited to a public repository. For example, in addition to summary statistics, the data points behind means, medians and variance measures should be available. If there are restrictions on publicly sharing data—e.g. participant privacy or use of data from a third party—those must be specified.

Reviewer #2: Yes

**********

5. Is the manuscript presented in an intelligible fashion and written in standard English?

PLOS Global Public Health does not copyedit accepted manuscripts, so the language in submitted articles must be clear, correct, and unambiguous. Any typographical or grammatical errors should be corrected at revision, so please note any specific errors here.

Reviewer #2: Yes

**********

6. Review Comments to the Author

Please use the space provided to explain your answers to the questions above. You may also include additional comments for the author, including concerns about dual publication, research ethics, or publication ethics. (Please upload your review as an attachment if it exceeds 20,000 characters)

Reviewer #2: All comments have been addressed and this is an interesting addition to the toolkit of socioecological analysis of infectious disease/vbds.

**********

7. PLOS authors have the option to publish the peer review history of their article (what does this mean?). If published, this will include your full peer review and any attached files.

**Do you want your identity to be public for this peer review?**  If you choose “no”, your identity will remain anonymous but your review may still be made public.

For information about this choice, including consent withdrawal, please see our Privacy Policy.

Reviewer #2: No

**********
